# Estimating the Impacts of Future Extreme Heat on Dryland Threatened Mammals: An Australian Case Study

**DOI:** 10.1111/gcb.70872

**Published:** 2026-04-20

**Authors:** Jack Bilby, William K. Cornwell, Katherine Moseby

**Affiliations:** ^1^ Centre for Ecosystem Science, School of Biological, Earth, and Environmental Sciences University of New South Wales Sydney New South Wales Australia

**Keywords:** arid zone, climate change, conservation planning, extreme heat, heat stress, systematic review, thermal refugia, translocation

## Abstract

Extreme heat is an escalating threat to biodiversity, with dryland ecosystems particularly vulnerable. Using Australia's dryland mammals as a model system, we present a framework comparing baseline and future heat envelopes for 36 threatened species to quantify future heat exposure, identify potential refugia and evaluate the suitability of translocation sites. We conducted a systematic review to assess our understanding of these species' thermal ecology. Our analyses identified eight species (22%) as high risk (three rodents, two dasyurids, one bat, one wombat and one macropod), for which their current distributions are projected to substantially exceed both current and historical heat envelopes. Rodents were overrepresented as high‐risk species while bats and dasyurids were underrepresented, whereas body size, range extent and conservation status did not predict heat risk. High‐risk species typically had narrow heat envelopes and have contracted to regions near their historical thermal maxima. High‐risk species had rarely been translocated (50% of species), with only one moved to a site projected to remain within its historical thermal maximum under all climate futures. Most moderate‐risk species (12 of 14; 86%) have been translocated, but > 60% of translocation sites are projected to exceed current thermal maxima. Our review revealed substantial knowledge gaps, with ten species (28%) entirely absent from the thermal literature, and high‐risk species (mean = 1.4 studies) being less represented than moderate‐ and low‐risk species (mean = 4.7 and 6.3 respectively). Research was biased toward behavioural and physiological responses, while critical subjects such as in situ responses, functional traits and direct evidence of heat‐related consequences were limited and are urgently needed to guide adaptive management. Conservation in drylands must prioritise protection of thermal refugia, improved land condition and integrate climate projections in long‐term planning. Our combined framework provides a globally relevant screening tool to identify heat‐vulnerable species and direct conservation management priorities.

## Graphical abstract

Label: Dryland mammals and heat risk. Description: Using Australia's threatened dryland mammals as a model system, we developed a framework comparing historical, current and future heat envelopes to quantify exposure to novel thermal conditions, identify potential thermal refugia and evaluate the suitability of conservation translocation sites. Across 36 species, eight were identified as high risk, with future distributions projected to exceed both historical and current thermal limits, and a further twelve classified as moderate risk, often persisting in cooler refugia but likely to experience novel heat within their current ranges. Heat risk was linked to thermal envelope width rather than species traits such as body size or range extent. Integration with a systematic review revealed that species facing the greatest projected heat exposure are also the least studied and least translocated. This framework provides a transferable approach for identifying heat‐vulnerable species.

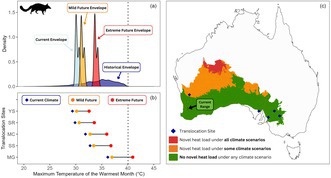

## 1 Introduction

Climate warming is among the most pervasive threats to global biodiversity, reshaping species distributions, population dynamics and ecosystem functioning (Brown et al. 2016; Pecl et al. 2017; Vardi et al. 2025). The increase in stochastic heatwaves and maximum seasonal temperatures, particularly during the warmest months, poses a growing threat to many organisms (Harris et al. 2018; IPCC 2022; Stillman 2019; Wang et al. 2021; Welbergen et al. 2008). These thermal pressures push local climates beyond the envelope in which species can persist, reducing performance, limiting activity periods and compromising overall fitness (Deutsch et al. 2008; Isotalo et al. 2022; Lee‐Yaw et al. 2016; Sinervo et al. 2010; Van de Ven et al. 2019; Wild et al. 2025). Sustained seasonal warming can impose long‐term physiological and ecological pressure, particularly on species already operating near their thermal limits (de la Fuente and Williams 2023; Harris et al. 2018; Meyer et al. 2022). In such cases, chronic exposure to rising seasonal maxima can trigger local extirpations or collapse of thermally sensitive populations, and in some cases cause persistent or irreversible ecological shifts (Harris et al. 2018; Meyer et al. 2022; Moseby et al. 2024; Sinervo et al. 2010; Vardi et al. 2025).

Arid and semi‐arid ecosystems are especially vulnerable to climate‐induced heat stress (IPCC 2022; Murali et al. 2023). These dryland regions already experience high ambient temperatures, low and unpredictable rainfall and pronounced diurnal fluctuations that are expected to intensify under future climate scenarios (IPCC 2022). Organisms inhabiting these landscapes often operate near their physiological limits, leaving little capacity to buffer further warming (Ma et al. 2023; Vale and Brito 2015; Wild et al. 2025). While multiple climatic drivers, including rainfall variability, humidity and winter temperature extremes, can influence species persistence, increases in summer temperatures are a key constraint on activity and fitness (Conradie et al. 2020; Cunningham et al. 2021; Fuller et al. 2021; Moore et al. 2026; Stiegler et al. 2023). Climate warming may reduce cold‐related constraints in winter, potentially lowering thermoregulatory costs and extending periods of activity during the cooler months (Asres and Amha 2014; Bonebrake et al. 2020; Wild et al. 2025). However, these potential benefits may be offset by increasing exposure to extreme summer temperatures and consequently any gains associated with reduced exposure to cold winter temperatures will be contingent on a species' capacity to tolerate exposure to extreme summer heat.

Dryland mammals are exposed to intense summer heat loads and have evolved a suite of behavioural and physiological strategies to cope with these conditions (Fuller et al. 2021; Mitchell et al. 2018). The pressures of summer heat loads may be exacerbated by the fact that some mammal species require daily activity to meet energetic demands, unlike other species which can remain inactive during heat extremes (Bondarenco et al. 2014; Han et al. 2024; Lundgren et al. 2024; Murali et al. 2023). Many species are nocturnal, thereby avoiding peak daytime temperatures and facilitating heat dissipation during foraging (Fuller et al. 2014; Grenot 1992; Withers et al. 2004). The suite of strategies includes thermally buffered shelter sites such as burrows or soil cracks (Dickman and Pavey 2023; Koertner and Geiser 2011; Read 1987), and physiological adaptations such as selective brain cooling (Hetem et al. 2012; Strauss et al. 2017), adaptive heterothermy (de Mel et al. 2024; Mitchell et al. 2002), use of torpor (Bondarenco et al. 2013, 2014; Nowack et al. 2017), and vasodilation and saliva spreading to improve evaporative cooling (Needham et al. 1974).

Australia, the driest inhabited continent and the country with the worst mammal extinction rate, provides a valuable case study for assessing climate vulnerability and adaptation needs in threatened mammals (Burbidge et al. 2009; Morán‐Ordóñez et al. 2018; Woinarski et al. 2015). In contrast to dryland systems globally, where many mammals and birds respond to extreme heat by migrating seasonally to cooler or wetter areas (Gibson et al. 2022; Webber and McGuire 2022), Australian mammals are not generally known to migrate during hot summers (but see Bullen and McKenzie 2005), and many remain confined to small and often isolated, refugia. Compounding this, many species have experienced severe range contractions due to habitat loss and introduced predators, with some, such as bilbies ( *Macrotis lagotis* ) and plains mice ( *Pseudomys australis* ), retreating to the hottest and most arid remnants of their former distributions (Woinarski et al. 2015, 2026). Many other species, such as numbats ( *Myrmecobius fasciatus* ) and stick‐nest rats ( *Leporillus conditor* ), persist only in mesic or island refugia, potentially losing heat‐adapted traits once present in their extirpated arid populations (Kelly and Phillips 2016; Lavergne et al. 2010; Sorte et al. 2011). Together, Australia's extreme aridity, covering approximately 70% of the continent, combined with range restriction, lack of migratory behaviour and projected intensification of heat extremes may render Australian dryland mammals particularly vulnerable.

Here, we used Australia's drylands as a model system to examine species‐level exposure to novel heat conditions and evaluate potential conservation strategies applicable to mammal assemblages globally. While we acknowledge that a complex interplay of climatic factors influences species persistence in the drylands, we focus specifically on extreme summer temperatures as this has the potential to exceed physiological limits under climate warming and provides a consistent, comparable metric for assessing exposure to novel climatic conditions. We assess future heat load relative to current and historical thermal envelopes to identify at‐risk species, locate potential refugia and examine translocation sites as a potential management tool for mitigating the effects of increasing temperatures. We also conduct a systematic literature review to evaluate the scope and focus of existing research on the thermal ecology of these threatened species. Through our combined climate analyses and literature review, we present a globally relevant framework for assessing extreme heat risk on threatened species and identifying opportunities for climate‐informed conservation actions, including translocations.

## 2 Methods

### 2.1 Framework for Comparing Current, Historical and Future Heat Envelopes

We calculated the baseline (1981–2010) heat envelopes for each species, defined as the range of maximum temperatures (BIO5: maximum temperature of the warmest month at 2‐m above ground) across both their historical and current geographic distributions. We then assessed future predictions to determine the proportion of each species' current distribution projected to remain within either its current or historical heat envelope. Both historical and current distributions were included because many species have undergone > 90% range contractions and the historical distribution may better reflect each species' climatic tolerance. We defined thermal maxima as the upper limit of each of the current and historical heat envelopes. We compared future climate conditions at existing translocation sites to these current and historical thermal maxima to assess their potential suitability under projected climate scenarios.

### 2.2 Species Selection and Distribution Mapping

The dryland boundaries used in this investigation were defined using the Köppen major climate classifications (Appendix S1; BOM 2025). We assessed all mammal species listed as threatened under the IUCN Red List or the EPBC Act and included those with 10% or more of their current or historical distribution within the dryland landscape (see Dickman and Pavey 2023). For each species, we compiled both current and historical geographic distributions to account for extensive range contractions in Australian mammals since European colonisation. Historical distributions were constructed using occurrence records and expert consultation, while current distributions were adapted from Marsh et al. (2022), with translocation sites removed and boundaries refined using recent records. Full details of data sources, spatial filtering and distribution construction are provided in Appendix S1.

This resulted in the inclusion of 34 threatened mammal species. Two additional conservation significant population units were included: the Pilbara population of the orange leaf‐nosed bat ( *Rhinonicteris aurantia* ), which is listed as a vulnerable population (EPBC Act 1999), and the central Australian populations of the brushtail possum ( *Trichosurus vulpecula* ), given the restricted extent of these populations and candidature for reintroduction back into the dryland regions (Cooper et al. 2018; McDonald et al. 2015). For these species, historical distributions encompassed the full species range, while current distributions were restricted to the focal population units. Henceforth, unless otherwise stated, conservation status refers to IUCN listings.

### 2.3 Assessing Baseline and Future Heat Envelopes

We used CHELSA v2.1 climatology data (Karger et al. 2017, 2021) to represent baseline heat exposure using a thirty‐year average from 1981 to 2010 and compared it to 15 future climate scenarios (five global circulation models × three shared socioeconomic pathways; SSP1‐2.6, SSP3‐7.0 and SSP5‐8.5) projected for 2041–2070. Although many species disappeared from parts of their historical distribution prior to the baseline heat exposure period, we used CHELSA v2.1 consistently to define both current and historical heat envelopes as it uses statistical downscaling, offering a more realistic representation of temperature variation in complex or remote terrains compared to alternatives derived from spatial interpolation, especially where weather stations are sparse (Jeffrey et al. 2001). For each species, we defined the baseline heat envelopes as the range of BIO5 values across either its current or historical geographic distribution. Future heat envelopes were defined as projected BIO5 values across each species' current distribution under each future climate scenario. Analyses were conducted in R (R Core Team 2025) using the *sf* (Pebesma 2018) and *terra* (Hijmans et al. 2022) packages.

**FIGURE 1:**
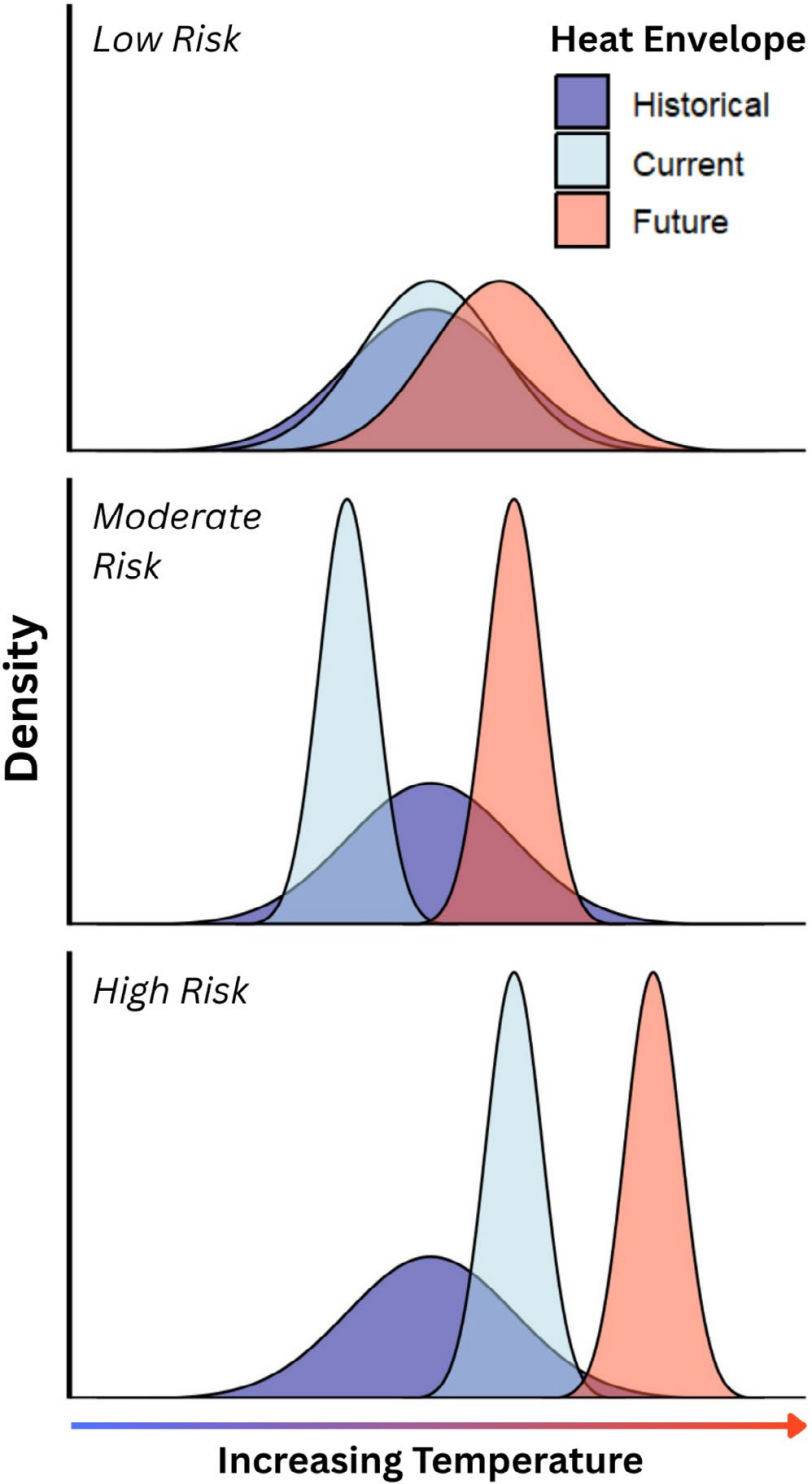
A conceptual diagram of density plots representing heat classifications. Risk category is based on overlap between both the historical (dark blue) and current (light blue) heat envelopes of a species, with the projected future (red) heat envelopes.

To assess vulnerability to future heat, we placed species into three risk categories utilising the mean across the 15 scenarios. Species were classified as low risk if > 50% of their future heat envelope remained within both their current and historical heat envelopes. Moderate‐risk species retained > 50% overlap with their historical heat envelope, but < 50% with their current envelope. High‐risk species had < 50% overlap with either envelope. To be conservative, species were assigned to a higher risk category if the standard error of the mean climate scenario overlapped with these criteria. Using this method, species facing novel heat under future warming compared to both their historical and current heat envelopes are classified as high risk; those exposed to novel heat only relative to their current envelopes are classified as moderate risk; and species with little or no novel heat exposure are classified as low risk (Figure 1).

We assessed predictors of risk by testing relations between risk categories and species‐specific traits. Categorical associations, taxonomic order and IUCN status were tested using Chi‐squared tests. For continuous variables, including range size (log‐transformed), body size (log‐transformed; Jones et al. 2009; Van Dyck et al. 2013), difference in area between historical and current range thermal maxima, historical and current heat envelope width, proportion of distribution in arid zones and percent of remnant habitat, we used Kruskal‐Wallis tests with post hoc Dunn's tests to assess differences across risk categories.

### 2.4 Evaluating Thermal Suitability of Translocation Sites

We assessed the differences in BIO5 between the historical thermal maxima and their translocation sites. Translocation sites were sourced from Legge et al. (2018) and supplemented by additional information by Woinarski et al. (2023), Crisp et al. (unpublished data) and a screening of the recent literature (Appendix S2). We excluded all failed translocations and any havens where species were remnant, rather than translocated, from analyses. In the case of the brushtail possum, we only included translocations into the dryland areas of its historic range. We treated each translocation site as a point location and assessed the BIO5 value from 1981 to 2010 and projected the mildest and most extreme future scenarios at the location to compare translocation site temperatures with thermal maxima to assess their potential suitability as thermal refugia.

### 2.5 Visualising Species‐Level Heat Exposure and Potential Refugia

**FIGURE 2:**
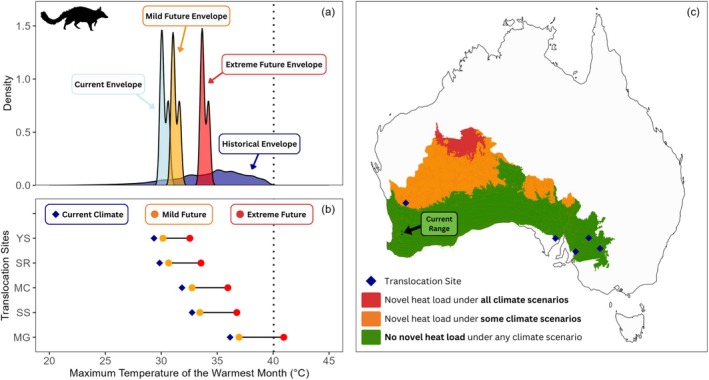
Annotated heat exposure profile for a moderate‐risk species, the numbat ( *Myrmecobius fasciatus* ). (a) Density plot of BIO5 (maximum temperature of the warmest month) across the historical (dark blue) and current (light blue) range under baseline climate (1981–2010), with the most mild (orange) and most extreme (red) future projections (2041–2070). Dotted line represents historical thermal maximum. (b) BIO5 values at each translocation site under baseline (blue diamond), mild (orange) and extreme (red) future scenarios. (c) Areas of the historical range projected to remain below the historical thermal maximum under all (green), some (orange) or no (red) climate scenarios by 2041–2070. The current range is marked with a black arrow and translocation site locations are shown by blue diamonds.

We produced species‐specific heat exposure plots for all threatened dryland mammals in Australia using *ggplot2* (Wickham 2016). These plots present the historical and current heat envelopes of the species and compare these to the mildest and most extreme future climate scenarios. We then compared these heat envelopes to all the current conservation translocation sites for the species, under current, mild and extreme future conditions. We generated species‐specific maps to visualise projected heat exposure across their distributions. For each species, we used the historical thermal maximum as a benchmark to identify areas in their historical range projected to exceed it under all, some or no future scenarios. These maps can be used to identify potential thermal refugia and priority areas for conservation or translocation. An annotated example of a heat exposure plot is presented in Figure 2 for the endangered numbat.

### 2.6 Systematic Review of Thermal Biology

We conducted a systematic review for all 36 species to assess the literature directly relevant to their thermal ecology. We compiled common names, binomial names and recent synonyms as listed by Baker and Gynther (2023). A Boolean search string was constructed where species names were combined with primary search terms, such as ‘heat’, ‘warming’ and ‘climate change’ (Appendix S3). We conducted literature searches using Web of Science and Scopus in June 2025 and adopted a PRISMA reporting procedure to screen the literature (see Appendix S3; Page et al. 2021). We obtained additional resources by applying a snowball technique using the reference section of publications as a source for other relevant papers (Greenhalgh and Peacock 2005; Rubenstein et al. 2023). Review papers were excluded from the final step but were used to find additional papers from their cited references.

We included any study that described a species' response to temperature, functional traits or processes related to heat exposure or thermoregulation. A Kruskal–Wallis test was then used to investigate biases in the average number of papers per species across the heat risk categories described previously. Papers were grouped into three broad themes: (1) individual responses, the observed behavioural or physiological adaptations of an individual animal in response to an increase in temperature and/or consequences of these events, (2) population responses, the population level responses of a species to an increase in temperature, observed or modelled and finally (3) innate traits or processes, which includes traits, behaviours or processes which will have a bearing on individual persistence and survival in extreme conditions but are not explored in this context, such as shelter site use, basal metabolic rate or pelage properties. These themes were further subdivided into nine key categories: behavioural response, physiological response, consequences, population response, morphological adaptation, climate predictions, thermal refuges, functional traits and functional processes (Table 1).

**TABLE 1:** The nine categories that papers were classified into. The three overarching themes are individual response (*I*), population response (*P*) and innate traits or processes (*In*).

Theme	Category	Definition
*I*	*Physiological response*	Recorded physiological change in response to change in temperatures (e.g., increase in evaporative water loss, metabolic rate increase, etc.)
*I*	*Behavioural response*	Recorded behavioural change in response to change in temperatures (e.g., changing foraging period, habitat selection, etc.)
*I*	*Consequences*	Consequences of extreme heat exposure (e.g., observed lethal and/or sub‐lethal effects)
*P*	*Population response*	Recorded population change in response to change in temperatures (e.g., population decline following extreme heat)
*P*	*Morphological adaptation*	Observed morphological specialisation for maintaining heat balance (e.g., intraspecific variation following Bergmann's or Scholander's rule)
*P*	*Climate predictions*	Use of empirical data to predict current and/or future climate suitability (mechanistic modelling or species distribution/MaxEnt modelling)
*In*	*Thermal refuges*	Specifics on shelter site and microclimate use in response to high temperatures (e.g., thermal buffering, change in shelter use, etc.)
*In*	*Functional trait*	Constant property of an animal which can be used in mechanistic modelling (e.g., surface heat conductance, pelage qualities)
*In*	*Functional process*	A descriptive property of an animal at one time state which may change across a temperature gradient (e.g., respiration, metabolic rate)

## 3 Results

### 3.1 Identifying Species at Risk From Future Heat Exposure

**FIGURE 3:**
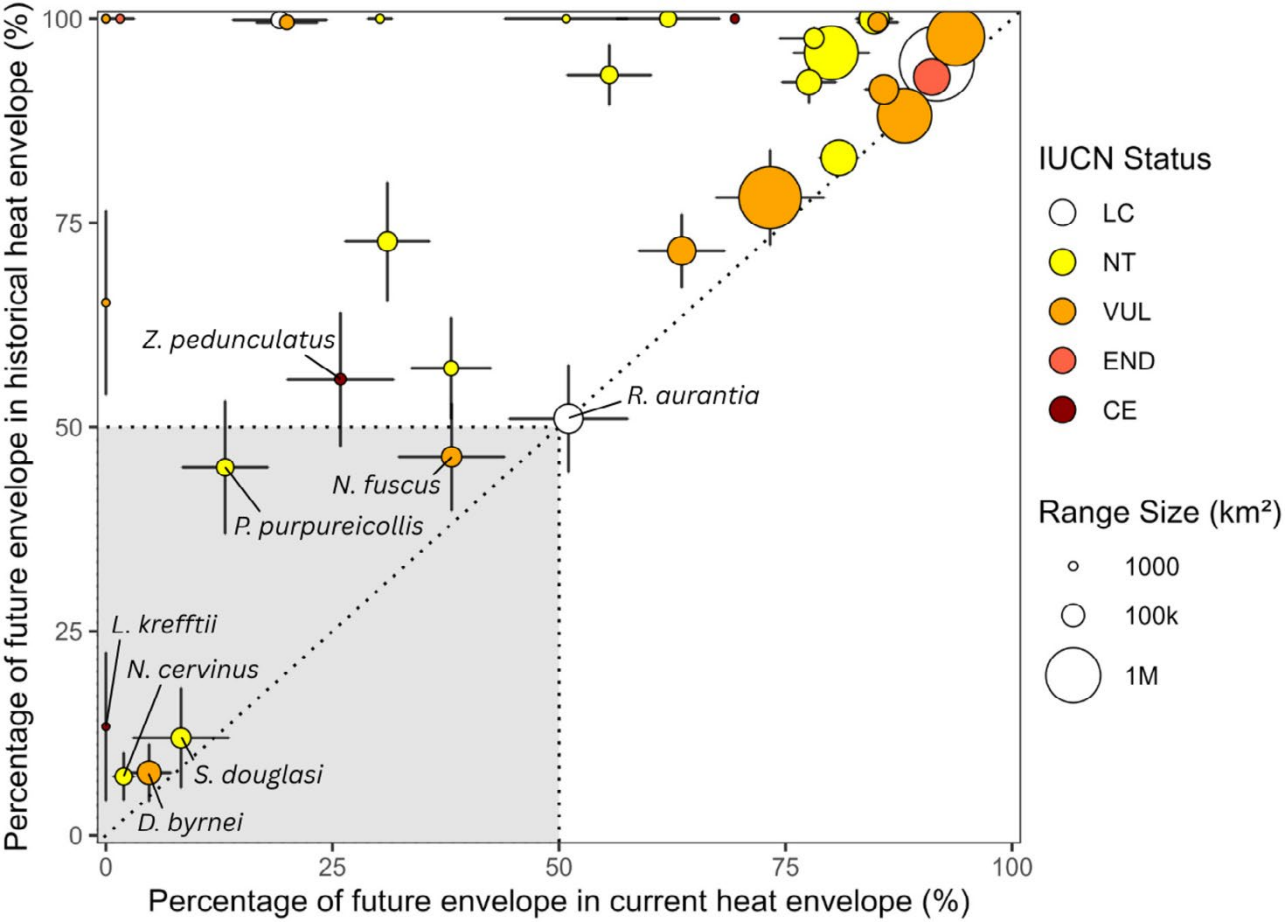
Future novel heat load across dryland threatened mammals. Each point shows the percentage of a species' future heat envelope (2041–2070) overlapping with its current versus historical heat envelope (baseline: 1981–2010); error bars reflect standard error across 15 climate scenarios. The shaded region includes six high‐risk species (< 50% overlap with both envelopes) and two additional species meeting this threshold under extreme scenarios. Species in the upper left are moderate risk (limited overlap with current but not historical envelopes); those in the upper right are low risk. Point colour indicates IUCN status; point size reflects current range area (log scale). See Appendix S4 for all species labels and climate projections.

**FIGURE 4:**
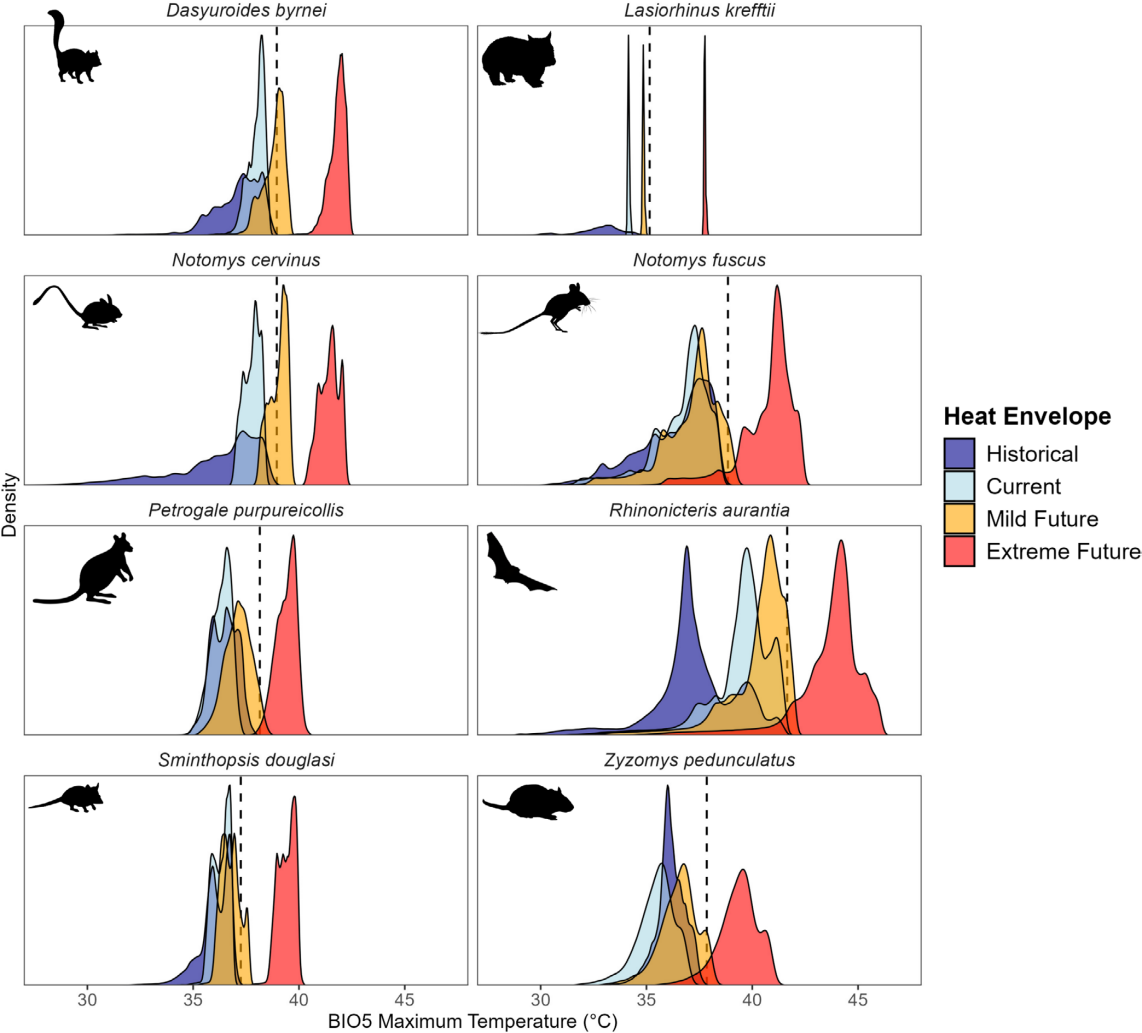
Density plots of BIO5 (maximum temperature of the warmest month) for eight high‐risk threatened species identified. These species are characterised by narrow heat envelopes near their historical thermal maxima. Envelopes represent historical (dark blue) and current (light blue) ranges under baseline climate (1981–2010), with mild (orange) and extreme (red) future heat envelopes (2041–2070) for the current range. Dashed line represents historical thermal maximum.

Climate projections indicate that a subset of threatened dryland mammals will face substantial exposure to conditions exceeding both their current and historical heat envelopes under future climate scenarios. Of the 36 dryland mammals assessed (Table 2), we identified six species (16.7%) at high risk, defined as having less than 50% of their future heat envelopes within both their current and historical heat envelopes under most future climate scenarios (Figure 3; with a fully annotated version provided in Appendix S4). Two additional species were conservatively classified as high‐risk due to overlap between the standard error of the mean and the high‐risk threshold, bringing the total to eight species of concern (Figure 4).

**TABLE 2:** The 36 threatened dryland species included in this investigation. The taxonomic order, conservation status (IUCN), body size, total decline in distribution since European colonisation, and novel heat risk are shown.

Name	Species	Order	IUCN	Size	Decline (%)	Heat Risk
Kowari	*Dasyuroides byrnei*	Da	VUL	109 g	86.1	High
Northern hairy‐nosed wombat	*Lasiorhinus krefftii*	Di	CE	31.9 kg	99.9	High
Fawn hopping mouse	*Notomys cervinus*	Ro	NT	35 g	97.0	High
Dusky hopping mouse	*Notomys fuscus*	Ro	VUL	39 g	88.9	High
Purple‐necked rock‐wallaby	*Petrogale purpureicollis*	Di	NT	4.5 kg^a^	81.1	High
Orange leaf‐nosed bat^b^	*Rhinonicteris aurantia*	Ch	LC	9 g	—	High
Julia Creek dunnart	*Sminthopsis douglasi*	Da	NT	55 g	57.0	High
Central rock rat	*Zyzomys pedunculatus*	Ro	CE	100 g	98.2	High
Boodie	*Bettongia lesueur*	Di	VUL	1.4 kg	99.9	Moderate
Ampurta	*Dasycercus cristicauda*	Da	NT	100 g	90.0	Moderate
Rufous hare‐wallaby	*Lagorchestes hirsutus*	Di	VUL	1.4 kg	99.9	Moderate
Banded hare‐wallaby	*Lagostrophus fasciatus*	Di	VUL	1.9 kg	99.9	Moderate
Greater stick‐nest rat	*Leporillus conditor*	Ro	NT	329 g	99.9	Moderate
Numbat	*Myrmecobius fasciatus*	Da	END	511 g	99.9	Moderate
Bridled nailtail wallaby	*Onychogalea fraenata*	Di	VUL	5 kg	99.9	Moderate
Shark Bay bandicoot	*Perameles bougainville*	Pe	VUL	231 g	99.9	Moderate
Carpentarian pseudantechinus	*Pseudantechinus mimulus*	Da	NT	17 g^a^	87.1	Moderate
Shark Bay mouse	*Pseudomys gouldii*	Ro	VUL	42 g	99.9	Moderate
Sandhill dunnart	*Sminthopsis psammophila*	Da	VUL	34 g	90.1	Moderate
Brushtail possum^c^	*Trichosurus vulpecula*	Di	LC	2.7 kg	—	Moderate
Woylie	*Bettongia penicillata*	Di	CE	1.2 kg	99.9	Low
Little pied wattled bat	*Chalinolobus picatus*	Ch	NT	6 g^a^	44.5	Low
Western quoll	*Dasyurus geoffroii*	Da	NT	1.1 kg	94.6	Low
Northern quoll	*Dasyurus hallucatus*	Da	END	471 g	81.9	Low
Golden bandicoot	*Isoodon auratus*	Pe	VUL	425 g	98.4	Low
Spectacled hare‐wallaby	*Lagorchestes conspicillatus*	Di	LC	2.8 kg	33.2	Low
Southern hairy‐nosed wombat	*Lasiorhinus latifrons*	Di	NT	26.2 kg	74.4	Low
Ghost bat	*Macroderma gigas*	Ch	VUL	124 g	76.0	Low
Greater bilby	*Macrotis lagotis*	Pe	VUL	1.2 kg	75.5	Low
Black‐flanked rock‐wallaby	*Petrogale lateralis*	Di	VUL	4.6 kg	79.6	Low
Yellow‐footed rock wallaby	*Petrogale xanthopus*	Di	NT	8.5 kg	85.2	Low
Red‐tailed phascogale	*Phascogale calura*	Da	NT	43 g	98.8	Low
Koala	*Phascolarctos cinereus*	Di	VUL	6.5 kg	41.1	Low
Plains mouse	*Pseudomys australis*	Ro	VUL	53 g	82.7	Low
Western mouse	*Pseudomys occidentalis*	Ro	NT	34 g	76.5	Low
Bristle‐faced freetail bat	*Setirostris eleryi*	Ch	NT	7 g^a^	80.0	Low

Abbreviations: IUCN: LC, least concern; NT, near threatened; VUL, vulnerable; END, endangered; CE, critically endangered. Order: Ch, Chiroptera; Da, Dasyuromorphia; Di, Diprotodontia; Pe, Peramelemorphia; Ro, Rodentia.

^a^ Data absent from PanTHERIA (Jones et al. 2009), sourced from Van Dyck et al. (2013).

^b^ Pilbara population—population listed vulnerable by the EPBC.

^c^ Central Australian population.

Of these high‐risk species, two of eight, the central rock rat ( *Zyzomys pedunculatus* ) and the northern hairy‐nosed wombat ( *Lasiorhinus krefftii* ), are listed as *Critically Endangered*, while two others are listed as *Vulnerable*, the kowari ( *Dasyuroides byrnei* ) and the dusky hopping mouse ( *Notomys fuscus* ). The remaining four include the *Near Threatened* fawn hopping mouse ( *Notomys cervinus* ), Julia Creek dunnart ( *Sminthopsis douglasi* ) and purple‐necked rock‐wallaby ( *Petrogale purpureicollis* ), as well as the Pilbara population of the orange leaf‐nosed bat ( *Rhinonicteris aurantia* ), listed as *Vulnerable* under the EPBC Act.

**FIGURE 5:**
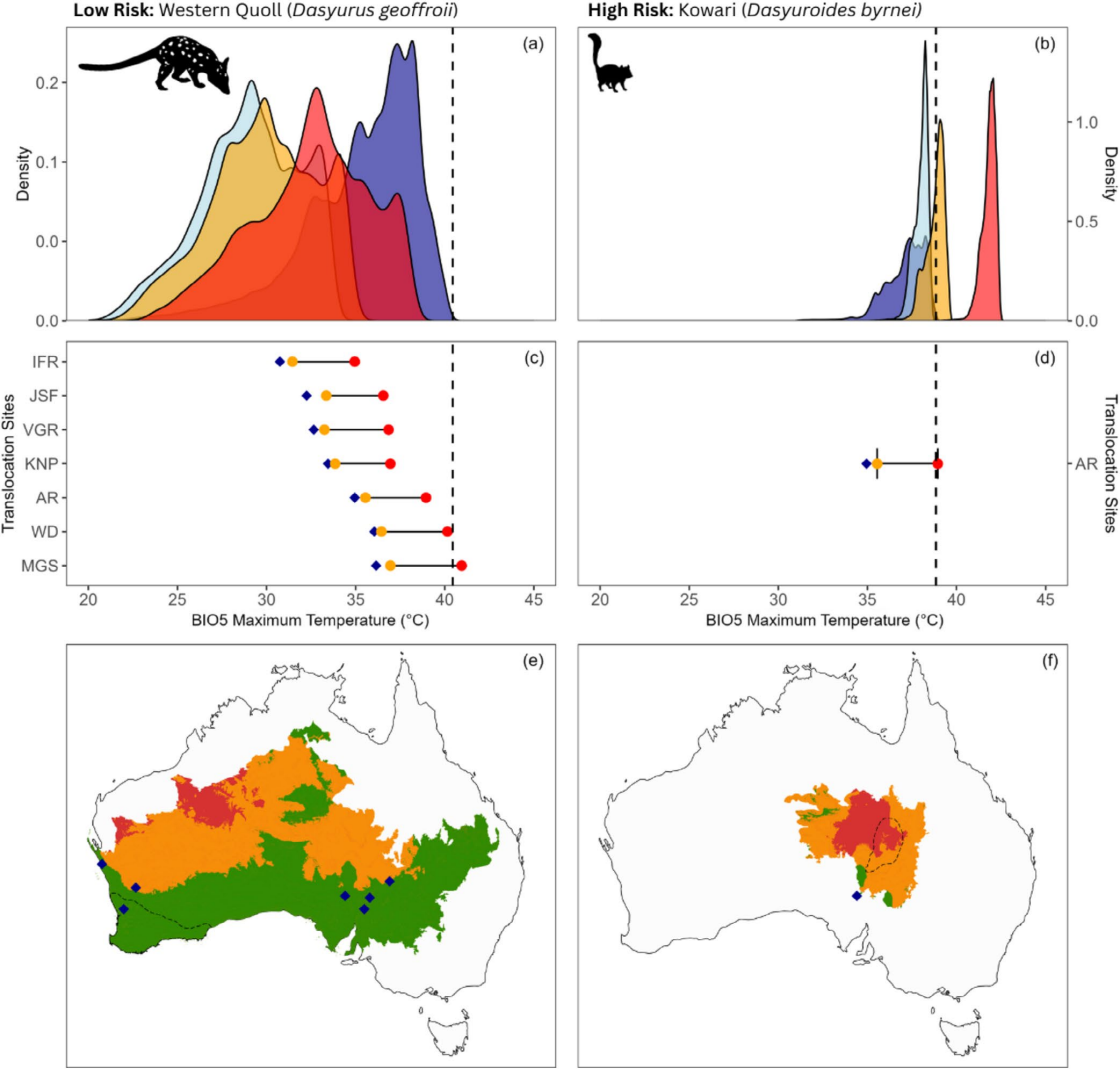
Heat profiles for a low‐risk (western quoll, *Dasyurus geoffroii* ) and high‐risk (kowari, *Dasyuroides byrnei* ) dasyurid species. (a, b) Density plots of BIO5 (maximum temperature of the warmest month) across historical (dark blue) and current (light blue) ranges under baseline climate (1981–2010), with mild (orange) and extreme (red) future heat envelopes (2041–2070) for the current range. Dashed line represents historical thermal maximum. (c, d) BIO5 projections at translocation sites under baseline (blue), mild (orange) and extreme (red) future scenarios. (e, f) Areas of the historical range projected to remain below the historical thermal maximum under all (green), some (orange) or no (red) climate scenarios by 2041–2070. Current range is shown by a dashed line; translocation sites are marked with blue diamonds.

An additional 12 threatened species (33.3%) were classified as moderate risk, with less than 50% of their future range within their current heat envelope but retaining more than 50% overlap with their historical heat envelope under at least one climate scenario. Species‐level profiles, following the structure shown in Figure 2, are presented for all 36 threatened dryland mammals in Appendix S5. The remaining 16 of 36 species were classified as low risk (44.4%). A direct comparison of a low‐risk species (the western quoll, *Dasyurus geoffroii* ) and a high‐risk species (the kowari) is provided in Figure 5 to demonstrate contrasting heat exposure under future climate conditions.

### 3.2 Traits Associated With Heat Risk

High‐risk species had both narrower historical heat envelopes and smaller differences between historical and current thermal maxima. Other traits were not significantly and consistently associated with high‐risk species (Appendix S6). We assessed risk group differences in the difference between historical and current thermal maxima, which reflects whether a species continues to occupy the hottest parts of its historical range or has retracted to cooler regions. Moderate‐risk species showed a significantly greater thermal difference (*χ* ^2^ = 14.3, *p* < 0.001; mean Δ = 9.46°C) than both low‐risk (mean Δ = 2.31°C) and high‐risk species (mean Δ = 0.48°C), suggesting retreat into cooler areas that may function as temporary refugia. No significant difference was found between low‐ and high‐risk species (*p* = 0.647).

Heat envelope width also differed among risk categories. High‐risk species had significantly narrower historical heat envelopes (mean = 9.1°C) than both low‐risk (mean = 18.3°C) and moderate‐risk species (mean = 19.2°C; *χ* ^2^ = 13.3, *p* = 0.001). Current heat envelope width was not significantly different between high‐risk (mean = 3.57°C) and moderate‐risk species (mean = 2.87°C) but was significantly greater in low‐risk species (mean = 13.0°C; *χ* ^2^ = 21.8, *p* < 0.001). High‐risk species are therefore characterised by a combination of narrow heat envelopes and small thermal differences between historical and current thermal maxima.

We found no significant associations with IUCN status (*χ* ^2^ = 5.61, *p* = 0.691) or taxonomic order (*χ* ^2^ = 5.94, *p* = 0.654). While no significant differences were observed, taxonomic patterns suggested underlying trends. Rodents had the highest proportion of high‐risk species (43%), with only 29% classified as low risk. In contrast, Peramelemorphs were predominantly low risk (67%) and had no high‐risk species. Diprotodonts and Dasyuromorphs showed more even distributions but still skewed toward moderate or low risk (Diprotodontia: 15% high, 39% moderate, 46% low; Dasyuromorphia: 22% high, 44% moderate, 33% low). Bats (Chiroptera) had the lowest proportion of high‐risk species (25%) and the highest proportion of low‐risk species (75%). Critically endangered species also showed a trend, with 67% falling into the high‐risk category, substantially higher than any other IUCN category (0%–33%).

When assessing other species characteristics, no significant differences for heat risk category were observed between body size (*χ* ^2^ = 0.848, *p* = 0.654) or the proportion of the current range or historical range occurring in arid regions (*χ* ^2^ = 3.96, *p* = 0.138 and *χ* ^2^ = 0.97, *p* = 0.616 respectively). However, total species area (*χ* ^2^ = 18.1, *p* < 0.001) and the percentage of remnant range (*χ* ^2^ = 15.3, *p* < 0.001) differed significantly among risk groups, with moderate‐risk species having smaller total ranges and lower remnant percentages than low‐risk species (*p* < 0.001). No significant differences were observed between high‐risk species and the other risk categories.

### 3.3 Thermal Suitability of Translocation Sites

**FIGURE 6:**
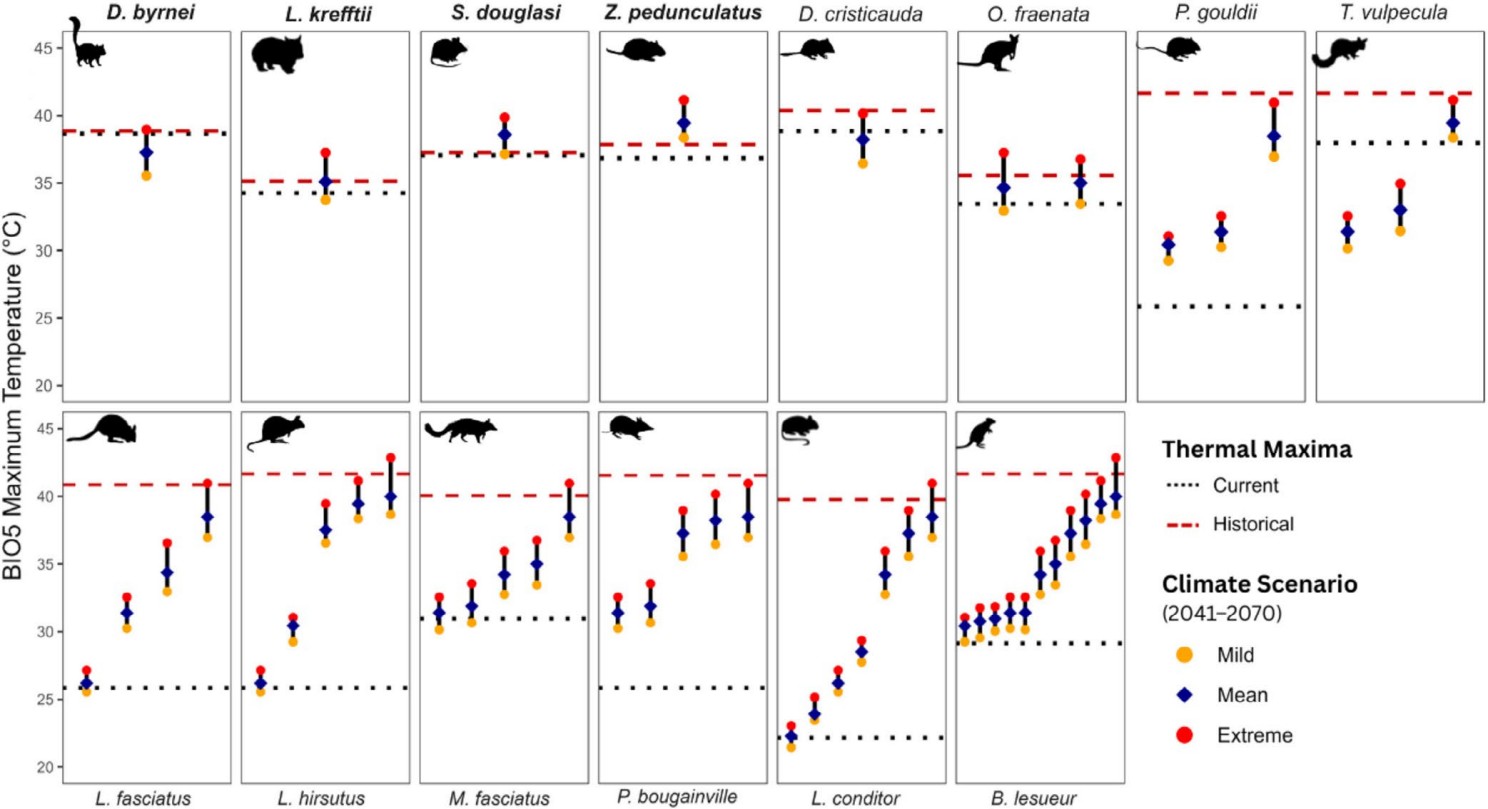
Projected heat exposure at translocation sites for four high‐risk and ten moderate‐risk species under future climate conditions (BIO5: 2041–2070). Each site shows the projected mean maximum temperature (dark blue), with error bars spanning the mildest (orange) to most extreme (red) future scenarios. Species' historical and current thermal maxima are indicated by red dashed and black dotted lines respectively. Species names in bold are classified as high risk.

Current translocation sites of the high‐risk species are unlikely to provide thermal refuge under climate change (Figure 6). Four of the eight high‐risk species have not been translocated to any additional sites, and the remaining four species have each only been translocated to one site (Table 3). Across these translocated high‐risk species, only the kowari has been released at a site that will remain below its current and historical thermal maximum under a mean future climate scenario. The northern hairy‐nosed wombat has been translocated to a site which will remain below its historical thermal maximum but will likely exceed the current thermal maximum. In contrast, the Julia Creek dunnart's translocation site is projected to exceed the historical thermal maximum under all but the mildest climate scenario, while the central rock rat's site exceeds historical maxima under all climate futures.

**TABLE 3:** Summary of thermal exposure across translocation sites for the dryland threatened species that have been translocated. For each species, we report the number of translocation sites assessed and the percentage of translocation sites projected to surpass current and historical thermal maxima (TM) under the mean future scenario (2041–2070) with the most mild and most extreme future climate scenarios shown in brackets. All eight high‐risk species are included even if the species has not been translocated in order to highlight future needs.

Species	Risk	Number of sites	Sites above current TM mean % (Mild–Extreme)	Sites above historical TM mean % (Mild–Extreme)
*Dasyuroides byrnei*	High	1	0% (0%–100%)	0% (0%–100%)
*Lasiorhinus krefftii*	High	1	100% (0%–100%)	0% (0%–100%)
*Sminthopsis douglasi*	High	1	100% (100%–100%)	100% (0%–100%)
*Zyzomys pedunculatus*	High	1	100% (100%–100%)	100% (100%–100%)
*Notomys cervinus*	High	0	—	—
*Notomys fuscus*	High	0	—	—
*Petrogale purpureicollis*	High	0	—	—
*Rhinonicteris aurantia*	High	0	—	—
*Bettongia lesueur*	Moderate	11	100% (100%–100%)	0% (0%–9.1%)
*Dasycercus cristicauda*	Moderate	1	0% (0%–100%)	0% (0%–0%)
*Lagorchestes hirsutus*	Moderate	5	100% (80%–100%)	0% (0%–20%)
*Lagostrophus fasciatus*	Moderate	4	100% (75%–100%)	0% (0%–25%)
*Leporillus conditor*	Moderate	7	100% (85.7%–100%)	0% (0%–14.3%)
*Myrmecobius fasciatus*	Moderate	5	100% (60%–100%)	0% (0%–20%)
*Onychogalea fraenata*	Moderate	2	100% (50%–100%)	0% (0%–100%)
*Perameles bougainville*	Moderate	5	100% (100%–100%)	0% (0%–0%)
*Pseudomys gouldii*	Moderate	3	100% (100%–100%)	0% (0%–0%)
*Trichosurus vulpecula*	Moderate	3	33.3% (33.3%–33.3%)	0% (0%–0%)
*Bettongia penicillata*	Low	11	63.6% (54.5%–63.6%)	0% (0%–18.2%)
*Dasyurus geoffroii*	Low	7	85.7% (42.9%–100%)	0% (0%–14.3%)
*Dasyurus hallucatus*	Low	2	0% (0%–0%)	0% (0%–0%)
*Isoodon auratus*	Low	7	14.3% (0%–57.1%)	0% (0%–14.3%)
*Lagorchestes conspicillatus*	Low	1	0% (0%–0%)	0% (0%–0%)
*Lasiorhinus latifrons*	Low	1	0% (0%–0%)	0% (0%–0%)
*Macrotis lagotis*	Low	13	0% (0%–7.7%)	0% (0%–7.7%)
*Petrogale lateralis*	Low	7	0% (0%–0%)	0% (0%–0%)
*Phascogale calura*	Low	6	66.7% (66.7%–100%)	0% (0%–0%)
*Phascolarctos cinereus*	Low	6	0% (0%–0%)	0% (0%–0%)
*Pseudomys australis*	Low	2	0% (0%–0%)	0% (0%–0%)

Most moderate‐risk species (12 out of 14; 86%) have been translocated to at least one additional site (Table 3), with only the Carpentarian pseudantechinus ( *Pseudantechinus mimulus* ) and sandhill dunnart ( *Sminthopsis psammophila* ) lacking translocations. Under a mean climate future, 61.4% of these sites are projected to exceed the species' current thermal maxima with 21.6% also exceeding historical thermal maxima (Figure 6). Among low‐risk species, 11 of 16 (68.8%) have been translocated. However, a higher proportion of their translocation sites (82.6%) are expected to exceed current thermal maxima, while only 10.4% exceed historical maxima.

### 3.4 Key Knowledge Gaps in Each Heat‐Risk Category

Research on thermal ecology in threatened dryland mammals was unevenly distributed and research focus was poorly aligned with species identified as being most vulnerable to future heat exposure. Following PRISMA‐based filtering (Appendix S3), 152 peer‐reviewed publications were identified as directly relevant to the thermal ecology or heat sensitivity of threatened dryland mammals (Appendix S7). These studies were grouped into three broad research themes: individual responses (e.g., behavioural shifts or thermoregulation), population‐level responses (e.g., distribution changes or demographic trends) and innate traits/processes (e.g., fur properties, shelters or physiological traits not directly tested under warming) with nine subcategories in total (Table 1).

**FIGURE 7:**
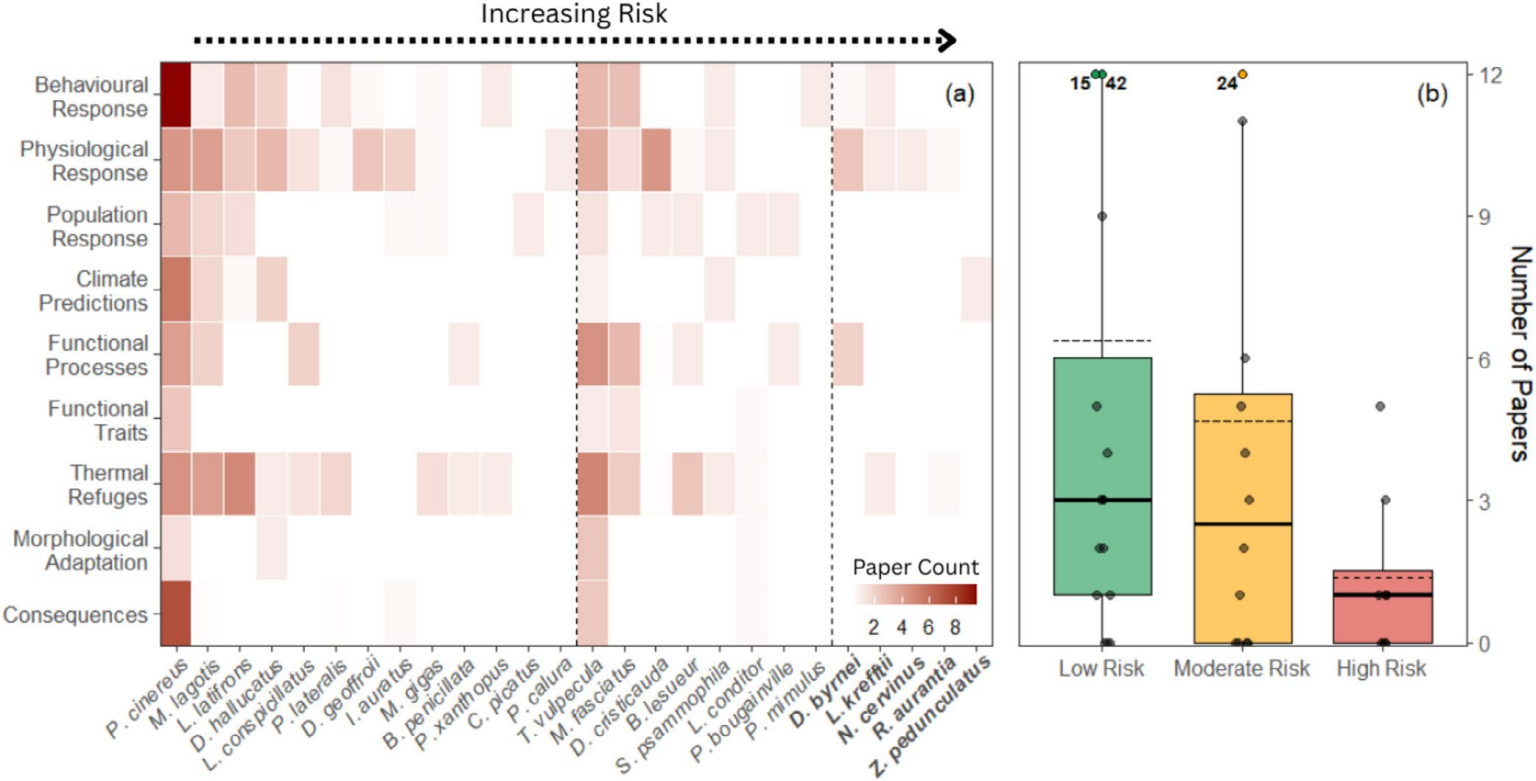
Research focus and coverage across threatened dryland mammals, grouped by heat risk category. (a) Heatmap showing the weighted number of publications per species (columns) across nine thermal research themes (rows). For multi‐focus studies, paper counts were split fractionally across relevant categories. Species are arranged from lowest to highest heat risk (left to right), with dashed lines separating low‐, moderate‐ and high‐risk groups. Only 26 of 36 species (72.2%) had any literature coverage. (b) Total number of publications per species, grouped by risk category. Boxplots indicate the distribution of study counts within each group; dashed horizontal lines show group means. Notable outliers include the koala ( *Phascolarctos cinereus* , 42 studies), brushtail possum ( *Trichosurus vulpecula* , 24) and bilby ( *Macrotis lagotis* , 15).

Strong species biases were evident in the literature, with a mean of 6.3 and median of 3.0 studies per species but with ten of 36 species being completely absent (Figure 7a). Among the eight species classified as high risk, published thermal ecology studies were available for only five species: the kowari (*n* = 5), northern hairy‐nosed wombat (*n* = 3), fawn hopping mouse (*n* = 1), orange leaf‐nosed bat (*n* = 1) and central rock rat (*n* = 1), while no studies were identified for the dusky hopping mouse, Julia Creek dunnart or purple‐necked rock‐wallaby. Overall, species classified as high risk tended to be the focus of less published research (mean = 1.4 studies) than species classified as low risk (mean = 6.3) or moderate risk (mean = 4.7), although this relationship was not significant (Figure 7b: χ^2^ = 2.72, *p* = 0.26).

Research focuses also differed systematically among risk categories. For species identified as high risk, the literature was dominated by individual‐level responses (59.1%) and innate traits/processes (31.8%), with limited coverage of population‐level responses (9.1%). Within this group, physiological responses accounted for the majority of studies (43.2%), followed by functional processes (18.2%), behavioural responses (13.6%) and thermal refuges (13.6%). Notably, no studies directly addressed heat‐related consequences, population responses or morphological adaptation for any high‐risk species. In contrast, moderate‐risk species showed a greater emphasis on innate traits and processes (43.2%), alongside individual responses (38.7%), but similarly had limited coverage of population‐level outcomes (8.6%). Low‐risk species exhibited the most balanced coverage, with both individual responses (47.9%) and innate traits (31.9%) well represented, and a higher proportion of population‐level studies (12.1%).

Only 83 of the 152 papers (54.6%) explicitly addressed exposure to extreme temperatures (defined here as > 35°C), and these were unevenly distributed across heat‐risk categories. Low‐risk species accounted for the majority of papers addressing extreme temperatures (59 papers, ten species), followed by moderate‐risk species (24 papers, six species), while high‐risk species were represented by only six papers across three species. Overall, extreme heat studies were identified for 62.5% of low‐risk species, 50.0% of moderate‐risk species and just 37.5% of high‐risk species. This imbalance was even more pronounced when limited to just field‐based evidence: in situ extreme heat studies involved nine low‐risk species (43 papers) and six moderate‐risk species (14 papers), but only a single high‐risk species, the northern hairy‐nosed wombat, represented by three papers. Together, these patterns indicate that species projected to be most vulnerable to future heat exposure are also those for which empirical evidence under extreme thermal conditions, particularly from field studies, is most limited.

### 3.5 Documented Responses and Impacts of Extreme Heat

Empirical evidence for heat sensitivity and responses in threatened dryland mammals differed markedly among heat‐risk categories and was strongly shaped by the literature biases identified above. For species classified as high risk, direct evidence of heat‐related mortality, fitness loss or population decline was limited or entirely absent. Available studies for this group were few and largely restricted to physiological experiments conducted under captive or controlled conditions. For example, laboratory studies on kowari demonstrated an inability to maintain normothermia at air temperatures of 40°C, with mortality occurring in some individuals (two of six; 33%) despite increased evaporative water loss, postural changes and increased conductance (Smith and Dawson 1984). Beyond these physiological investigations, no studies documented population responses or heat‐related consequences for most high‐risk species, and in situ evidence was available for only the northern hairy‐nosed wombat, where studies documented reduced surface activity, strong reliance on burrows and behavioural buffering during hot conditions (Evans et al. 2003; Johnson 1991; Treby et al. 2007).

Moderate‐risk species were represented by a broader, though still limited, evidence base that included physiological and behavioural responses to elevated temperatures, alongside a small number of population‐level assessments. Behavioural adjustments were frequently documented, such as reduced activity or shifts to cooler microhabitats. For example, numbats ( *Myrmecobius fasciatus* ) exhibited reductions in activity during hot conditions, consistent with behavioural thermoregulation (Christensen et al. 1984; Cooper and Withers 2024). Population‐level responses were again seldom reported for moderate‐risk species. At Arid Recovery, declines in Shark Bay bandicoots ( *Perameles bougainville* ) and stick‐nest rats were observed during periods of extreme heat, with mortality recorded in the latter, while burrowing species persisted (Bolton and Moseby 2004; Moseby et al. 2024). Despite these examples, most studies of moderate‐risk species focused on short‐term behavioural or physiological responses and did not assess cumulative heat exposure, fitness consequences or demographic rates directly.

In contrast, low‐risk species were better represented in the empirical literature across all response categories. Several low‐risk species showed population‐level responses to extreme heat, including declines following heatwaves or hot summers. For example, koalas ( *Phascolarctos cinereus* ) were reported to retreat to shaded riparian habitats during hot, dry periods, with population declines observed following heatwaves (Bird 2009; Gordon et al. 1988; Seabrook et al. 2011; Smith et al. 2013). Behavioural thermoregulation was also frequently documented in this group, including activity reductions, postural adjustments and increased use of shade or artificial water sources. Physiological heat‐tolerance experiments conducted on some low‐risk species, such as bilbies, demonstrated limits to thermoregulation under acute heat exposure, although these studies were conducted under captive conditions (Hulbert and Dawson 1974).

Across low and moderate‐risk species, associations between thermal exposure and shelter availability were commonly reported. Burrows constructed by bilbies, wombats and boodies ( *Bettongia lesueur* ) were repeatedly identified as important thermoregulatory refuges, both for the focal species and for broader vertebrate communities (Dawson et al. 2019; Hofstede and Dziminski 2017; Pike and Mitchell 2013; Read et al. 2008; Thornett et al. 2016). Other habitat features, including old‐growth spinifex, tree hollows and rocky outcrops, were also identified as important thermal refuges for a number of species, although their availability has declined due to wildfire, grazing and land clearing (Cooper and Withers 2005; Cowan et al. 2020; Dawson and Bennett 1978; Isaac et al. 2008; Riley 2020).

Finally, modelling approaches used to infer future heat vulnerability were sparse and unevenly distributed across risk categories. Correlative climate or distribution models were identified for only seven of the 36 species assessed, including the high‐risk central rock rat (McDonald et al. 2018). Mechanistic biophysical models were almost entirely absent for threatened dryland mammals, with the notable exception of the koala (Briscoe et al. 2016), a pattern that may reflect the paucity of underlying parameter data. Across all risk categories, evidence was dominated by behavioural and physiological responses, whereas key data on functional traits, morphology and functional processes were scarce. Critically, no high‐risk species had documented functional trait or morphological data, and only a single high‐risk species was represented by any functional process information.

## 4 Discussion

Climate warming is increasingly recognised as a potential driver of biodiversity loss across the world's dryland ecosystems (Gardner et al. 2022; Ma et al. 2023; Murali et al. 2023; Vale and Brito 2015), yet systematic frameworks for assessing species‐level vulnerability remain scarce and are further complicated by human‐induced, non‐climatic range contractions. Here, we present a replicable framework that combines range‐wide climate modelling, translocation sites and species traits to assess exposure to future heat extremes. Applied to threatened mammals of Australia's drylands, our analyses estimate unprecedented heat exposure within decades, with eight species facing range‐wide conditions exceeding most of their current and historical thermal envelopes. The most vulnerable species were also among the least translocated and least studied, with significant knowledge gaps on population responses, functional traits and consequences of extreme heat. As summer temperatures increase globally, this framework offers a scalable model for other regions seeking to identify vulnerable species and mitigate risk under climate warming.

### 4.1 Predicting Risk of Heat Exposure

We identified eight high‐risk species whose distribution will fall outside both their historical and current heat envelopes under most future scenarios, indicating exposure to novel heat. These species are characterised by a combination of having narrow historical and current heat envelopes and continued occupancy near their historical thermal maxima. Predictors such as body size, range size, taxonomic group, arid zone endemism and IUCN status did not predict high‐risk status, suggesting that exposure to novel heat is more closely linked to heat envelope width than to distribution extent or intrinsic species attributes.

The pattern is consistent with broader evidence that climate generalists may be more resilient to environmental change, a pattern observed across taxa globally (Boyles et al. 2011; Iknayan and Beissinger 2020; Sweeney and Jarzyna 2022). In contrast, species with narrow historical and current heat envelopes already near their thermal maxima may have little evolutionary or ecological room to manoeuvre as climate extremes intensify. Opportunities for range shifts of these high‐risk species into more suitable areas fitting their functional niche limits may be restricted due to pressures from invasive predators (Moseby et al. 2011; Woinarski et al. 2026). Furthermore, altered fire regimes and overgrazing by livestock, invasive herbivores and native macropods can alter crucial habitat structure, further exacerbating impacts of predation and heat exposure (Cunningham et al. 2019; Legge et al. 2011; McGregor et al. 2014; Mills et al. 2020; Tulloch et al. 2023). Consequently, landscape‐scale management of invasive species and land condition is likely to be a key prerequisite for enabling range expansion or assisted colonisation to climatically suitable habitat (Cullen et al. 2025; Haby and Brandle 2018; Legge et al. 2019; Mills et al. 2020; Moseby et al. 2011; Pedler et al. 2016).

A further twelve species were classified as moderate risk, retaining less than 50% overlap with their current heat envelope but remaining largely within their historical thermal niche. These species exhibited large differences between historical and current thermal maxima, consistent with range contraction to cooler parts of the historical distributions, such as islands or other mesic refugia, and are likely to experience novel heat within their current range. While these cooler refugia may buffer contemporary heat exposure, loss of access to hotter parts of their historical range may have eroded ancestral genetic and phenotypic variation linked to heat tolerance (Bellis et al. 2020; Carvalho et al. 2019; Weeks et al. 2011). If this adaptive potential is not present in contemporary populations, these species may be just as vulnerable to climate warming and extreme heat events as high‐risk species (Bellis et al. 2020; Kelly and Phillips 2016; Lee‐Yaw et al. 2016; Weeks et al. 2011).

### 4.2 Translocation as a Management Tool and Research Opportunity

Translocations, whether within historical ranges or through assisted migration, offer an actionable conservation strategy to mitigate the effects of increasing heat load. While high‐risk species had disproportionately few translocation sites, many other threatened dryland mammals have already been translocated to regions projected to remain climatically suitable (Figure 6). Translocation strategies can be broadly categorised into those moving source populations into: (1) climate‐matched sites; (2) cooler refugia; and (3) warmer areas to evaluate adaptive capacity and assess responses to novel conditions. This decision should be tailored to a species' thermal vulnerability, as assessed through current and historical heat envelopes, and reinforced with a more comprehensive modelling approach (Bellis et al. 2020; Butt et al. 2021). Species‐specific climate profiles provided in Appendix S5 provide a starting point for conservation managers to explore the viability of these options.

In Australia, translocations of threatened mammals have expanded substantially over the past decade, particularly within fenced reserves and island refuges, reflecting their central role in contemporary conservation strategies (Legge et al. 2018; Woinarski et al. 2023). Our results suggest that species in the high‐risk category should be prioritised for translocation into cooler environments, including assisted migration outside their historic range, to establish refuge populations. Four of the eight high‐risk species have no translocation sites at all, while the remaining four have each only been translocated to a single site (Figure 6). Of these, only the kowari has been moved to a location projected to remain within its current or historic thermal envelope under most future scenarios. This highlights an urgent need to identify and manage climatically suitable sites within a species' functional niche to ensure their persistence in a changing climate.

For moderate‐risk species where remnant populations only exist at the coolest edges of the historical range, reintroduction into warmer environments may be unavoidable if the aim is to restore populations across their former range. However, this presents a trade‐off: while such translocations may help assess whether adaptive traits are retained or expressed, they also expose individuals to conditions that may exceed their physiological limits. Nearly two thirds of the existing translocations for moderate‐risk species are projected to exceed the current envelope under a mean climate future, with many sites already exceeding a species' current thermal maxima, and reintroductions into these areas have produced mixed results (Bolton and Moseby 2004; Northover et al. 2023). There is a risk that remnant populations, restricted to cooler refugia, may lack the physiological and behavioural traits previously present in extirpated populations (Bellis et al. 2020, 2019; Braidwood et al. 2018).

Any consideration of translocations into warmer environments must be approached cautiously and framed as a stepwise evaluation of adaptive capacity. Careful ecological evaluation should include the use of correlative or mechanistic niche models to quantify suitability and forecast exposure to extreme heat, combined with releases into sites that incrementally increase the magnitude of temperature increase (Bellis et al. 2024; Butt et al. 2021). Monitoring of survival, behaviour, body condition and trait expression under these conditions would allow assessment of tolerance limits before progressing to more extreme environments. Targeted management interventions, such as provision of artificial shelters or supplementary resources, may help buffer short‐term extremes (Cowan et al. 2020; Watchorn et al. 2022; West et al. 2017). Together, such approaches may provide a structured pathway to evaluate whether translocations into warmer regions are viable, while minimising the risk of population failure. However, despite their widespread use, translocations are rarely designed to explicitly test climate suitability or adaptive capacity, and outcomes are not consistently monitored or reported.

Our range‐wide heat projections provide suggestions for areas with translocation potential that may improve long‐term persistence of threatened dryland mammals under climate change. However, we acknowledge that there are a range of intrinsic and extrinsic factors beyond a single heat metric that determine translocation outcomes and should thus be utilised as just the first step in release site selection. In Australia especially, the presence of introduced foxes and cats is a major factor predicting reintroduction failure (Hardman et al. 2016; Moseby et al. 2011). Furthermore, intrinsic factors such as hyperdispersal and release group size can have a major influence on translocation outcomes (Bilby and Moseby 2024; Germano and Bishop 2009; Komers and Curman 2000). Translocating species can also significantly alter the ecosystem at release sites and trigger trophic cascades, although reintroductions into areas where species have been extirpated have the potential to benefit the ecosystem through reinstating lost processes (Beschta and Ripple 2016; Gibb et al. 2021; Svenning et al. 2016).

In practice, many dryland mammals require managed sites with invasive predator control to persist, meaning suitable refugia may be inaccessible unless threats such as cats and wildfire are actively suppressed (Legge et al. 2018; Moseby et al. 2011; Tulloch et al. 2023). Assisted migration remains controversial but is increasingly advocated under high‐emissions scenarios where few suitable refugia persist in their current or historical distribution (Hoegh‐Guldberg et al. 2008; Lundgren et al. 2024; Twardek et al. 2023). For instance, the Arid Recovery reserve lies just outside the kowari's known historical distribution yet is projected to stay below its thermal maxima under all but the worst‐case scenarios. In this case, and for other high‐risk species, assisted migration may offer a viable and necessary insurance strategy.

### 4.3 Gaps in the Literature

Our systematic review revealed major gaps in knowledge of thermal ecology for threatened dryland mammals, with high‐risk species among the most understudied. This uneven research effort may hinder the development of targeted and effective management strategies. Most studies examined individual responses (e.g., physiological changes, behavioural shifts, etc.), with much of this evidence derived from laboratory studies, which may not fully capture the physiological and ecological challenges of extreme heat in dryland systems. Increased accessibility and miniaturisation of modern biologging technologies, such as GPS units, accelerometers and environmental loggers, offer opportunities to study rare and cryptic species in the field and at high resolution without the need for consistent observation and disturbance (Buchholz et al. 2019; English et al. 2024; Tomkiewicz et al. 2010; Wilmers et al. 2015). These technologies can overcome long‐standing biases in the literature that have favoured larger, more visible species such as koalas, possums and wombats. Given that high‐risk species tended to be the least studied and least translocated, these shortfalls represent a critical barrier to climate‐informed conservation planning.

There is a considerable and active global and Australia‐specific field of literature on the physiology of dryland endotherms (Fuller et al. 2026; McKechnie and Wolf 2019; Withers et al. 2016). However, in the case of the threatened species considered here, physiological assessments were relatively common before the 1990s, but have decreased in recent decades. Although earlier methods remain valuable, the lack of protocol standardisation between studies limits interspecies comparisons (Killen et al. 2021; Kingsolver and Umbanhowar 2018; Terblanche et al. 2007). Without diligent recording of variables such as acclimation time, ambient humidity and observed behaviours or posture, interpreting results in ecological context remains challenging (Kingsolver and Umbanhowar 2018; Terblanche et al. 2007). Likewise, large temperature jumps, particularly between 30°C and 40°C, and low maximum experimental temperatures can obscure the true threshold at which a species leaves its thermoneutral zone, limiting the utility of these data in mechanistic models and conservation management (Evans et al. 2015; Killen et al. 2021; Kingsolver and Umbanhowar 2018). As a result, while broader ecophysiological insights from related dryland species may provide useful context, their transferability to threatened taxa remains uncertain without targeted, standardised study and careful extrapolation. Addressing these gaps is critical for improving understanding of key fitness thresholds, refining biophysical models and informing conservation management.

Despite widespread implementation in Australia, translocations remain underutilised as structured research opportunities to investigate responses to heat. Evaluating how translocation sites can function as climate refugia for high‐risk species or to foster emergent traits in mesic source populations should be a research and management priority (Kelly and Phillips 2016; McDonald et al. 2015; Sgrò et al. 2011; Weeks et al. 2011). Evidence of intraspecific variation in heat responses, including lower metabolic rates, reduced evaporative water loss, and smaller body sizes in arid populations of marsupials like possums, koalas and stick‐nest rats, highlights the importance of selecting arid‐adapted source populations where possible (Briscoe et al. 2015; Cooper et al. 2018; Onley et al. 2022; Stobo‐Wilson et al. 2020; Yom‐Tov and Nix 1986). Yet, post‐release assessments rarely examine whether mesic‐sourced individuals can express these traits and have not examined the potential for behavioural plasticity to buffer short‐term climatic stress (Trewartha et al. 2025). In addition, monitoring is often limited in duration or scope, and unsuccessful outcomes are rarely published, restricting our ability to identify failure mechanisms and refine climate‐informed management. Reintroduction efforts to the drylands for species such as the woylie, numbat or brushtail possum have, out of necessity, released cohorts from remnant mesic populations. This provides a key opportunity to investigate the extent to which climate‐relevant traits can emerge in novel environments and to evaluate species' capacity for acclimatisation and plasticity under rising temperatures.

### 4.4 Limitations of Heat Envelopes Under Climate Extremes

The projections used here are based on maximum temperatures of the warmest month as a proxy for thermal stress, providing a useful indicator of species‐level heat exposure. However, no single climate metric can capture the full set of interacting processes that determine persistence in the drylands, including other climatic constraints, soil condition, hydrology, vegetation structure and disturbance regimes (Gemechu and Dalle 2023; Legge et al. 2019; Maestre et al. 2016). Heat stress arises from a combination of heat intensity, duration and frequency, together with the availability of resources and effectiveness of refuges that buffer exposure (Stillman 2019; Vardi et al. 2025). Species further modulate their exposure through changes to behaviour, activity and habitat use, and therefore their ability to cope with thermal stress is likely determined by cumulative thermal load, ecophysiological strategy and access to suitable microclimates rather than any single temperature metric (Scheffers et al. 2014). Mechanistic approaches, such as biophysical models, and assessment of microhabitat thermal buffering and selection are therefore needed to build on our results, particularly for the high‐risk species we identified, to provide a more holistic basis for management under climate change.

A key constraint on persistence is the presence or absence of effective microrefugia. Areas that remain within a species' heat envelope may still fail to support populations if they lack appropriate shelters, resources or structurally complex habitats that buffer the impacts of climate extremes (Harris et al. 2018; Kerr et al. 2025; Tourani et al. 2023). For example, brushtail possums and koalas persist in the drylands in rocky gorges and shaded riverine systems respectively, but are unlikely to occupy the surrounding landscape, particularly during hot summer conditions (Anderson et al. 2025; McDonald et al. 2015; Seabrook et al. 2011; Short and Turner 1994; Smith et al. 2013). Active conservation management, such as reducing grazing impacts, invasive species control and implementing appropriate fire regimes, is likely to improve availability and quality of key thermal refuges, like sand mounds and old‐growth spinifex (Burbidge and McKenzie 1989; Greenville et al. 2018; James et al. 1999; McKenzie et al. 2007; Reside et al. 2019). Enhancing habitat quality and structural complexity is therefore one of the few scalable interventions likely to mitigate heat exposure across dryland landscapes.

Species‐specific ecophysiology further mediates vulnerability to extreme heat in ways not captured by average temperature metrics. For instance, the strictly diurnal numbat, classed as a moderate‐risk species, will likely face greater heat exposure compared to nocturnal species (Cooper and Withers 2024; Northover et al. 2023). Body size also shapes thermal dynamics, with smaller animals gaining heat from their environment more rapidly due to a larger surface‐to‐volume ratio, yet they may access thermal refuges, such as subterranean burrows or soil cracks, more easily than larger species (Bonebrake et al. 2020; Fuller et al. 2021; Rezende and Bacigalupe 2015; Robinson and Morrison 1957). Together, these factors highlight the importance of ecological context and habitat utilisation in determining which species will be most vulnerable in the face of climate extremes.

Finally, even where projected average maximum temperatures remain within a species' heat envelope, the increasing intensity, duration and frequency of stochastic heatwaves may exceed physiological tolerances, particularly in landscapes lacking suitable microclimates or reliable water sources (Harris et al. 2018; Morán‐Ordóñez et al. 2018; Murali et al. 2023; Vardi et al. 2025). Notably, extreme heatwaves have played a driving role in the declines and extinctions of translocated populations of the stick‐nest rat and numbat at sites exceeding the current heat envelope but remaining below their historical thermal maxima (Moseby et al. 2024; Northover et al. 2023). In the case of stick‐nest rats, the population had persisted at the site for nearly two decades before a combination of factors, including extreme heat and drought, resulted in their decline and eventual extirpation (Moseby et al. 2024). These examples underscore the need to test how well heat envelopes predict persistence under episodic extremes and whether historical thermal exposure of source populations influences survival under such events.

## 5 Conclusions

Drylands cover over 40% of Earth's land surface and support diverse species uniquely adapted to extreme conditions, yet they are increasingly vulnerable under climate change (Maestre et al. 2016; Murali et al. 2023; Reynolds et al. 2007; Vale and Brito 2015). The framework we present, linking heat exposure profiles with thermal envelopes and translocations, offers a broadly applicable screening tool for identifying at‐risk species and prioritising climate‐informed interventions, which can then be further validated with more comprehensive modelling approaches prior to management action. Although developed using Australian dryland mammals, this framework is transferable to systems globally where species face increasing thermal extremes and provides a targeted approach for assessing heat exposure within the broader, multi‐process context of dryland conservation. Effective conservation in drylands under climate warming must prioritise thermal refugia, integrate historical climate thresholds and manage grazing and fire to enhance microclimate buffering. Translocations, including assisted migration, represent practical tools for promoting and researching persistence and adaptations under escalating heat stress (Bellis et al. 2024; Butt et al. 2021; Twardek et al. 2023). Advances in biologging and mechanistic modelling make it feasible to monitor and predict species' heat responses in their natural environments (English et al. 2024; Kearney et al. 2016; Wilmers et al. 2015) and should be a priority for threatened species research. Without urgent, climate‐informed and empirically supported action, many threatened species may be unable to keep pace with warming, and the unique ecological functions they support may be irreversibly lost.

## Author Contributions

**Jack Bilby:** conceptualization, methodology, data curation, investigation, formal analysis, visualization, writing – original draft, writing – review and editing. **William K. Cornwell:** conceptualization, methodology, supervision, writing – review and editing. **Katherine Moseby:** conceptualization, methodology, supervision, writing – original draft, writing – review and editing.

## Funding

This research was supported by ARC Future Fellowship FT210100173.

## Disclosure

Article impact statement: A globally relevant framework for identifying heat‐vulnerable dryland fauna, using Australia's threatened mammals as a case study.

## Conflicts of Interest

The authors declare no conflicts of interest.

## Supporting information


**Appendix S1:** The spatial data sources and procedures used to reconstruct historical and current geographic distributions for threatened dryland mammal species included in this study.

**Appendix S2:** Translocation sites sourced from Legge et al. (2018) and supplemented with information from Woinarski et al. (2023) and Crisp et al. (unpublished dataset).

**Appendix S3:** The PRISMA flow diagram for different phases of the systematic review. Boolean strings used to assess the literature for heat responses in the target species. Scientific name, recent synonyms and common names were chosen as stated by Baker and Gynther (2023).

**Appendix S4:** All individual climate projections and comprehensively labelled version of Figure 3. An assessment of future novel heat load of dryland threatened mammals. The percentage of a species' future range within their current climate envelope is compared to the percentage of a species' future range compared to their historic climate envelope. Colour indicates shared socioeconomic pathway in (a) and IUCN status in (b) and point size is representative of the species' current range on a logarithmic scale.

**Appendix S5:** Individual species profiles of heat load projections for all 36 threatened dryland mammals, listed alphabetically.

**Appendix S6:** Statistical associations between heat risk category and species‐level traits. Chi‐squared tests (*χ* ^2^) were performed for taxonomic order and IUCN status. Taxa: Ch, Chiroptera; Da, Dasyuromorphia; Di, Diprotodontia; Pe, Peramelemorphia; Ro, Rodentia. IUCN: CE, Critically Endangered; END, Endangered; LC, Least Concern; NT, Near Threatened; VUL, Vulnerable. Kruskal‐Wallis tests (*H*), with post hoc Dunn's tests (*z‐*statistic), were performed for numeric predictors.

**Appendix S7:** All relevant papers collated during systematic review and species attributes.

## Data Availability

All data and code supporting the findings of this study are available in the Dryad Digital Repository at https://doi.org/10.5061/dryad.8kprr4z3f. Climate data were derived from the CHELSA v2.1 dataset (Karger et al. 2017, 2021), which is available at https://doi.org/10.16904/envidat.228.
