# Supplementary material for: Estimating the Impacts of Future Extreme Heat on Dryland Threatened Mammals: An Australian Case Study
Source: Glob Chang Biol. 2026 Apr 20;32(4):e70872. doi: 10.1111/gcb.70872 (PMC13094399; doi:10.1111/gcb.70872)
Supplement: Supplementary file 1 — Appendix S1: The spatial data sources and procedures used to reconstruct historical and current geographic distributions for threatened dryland mammal species included in this study. [file GCB-32-e70872-s001.docx]

**Appendix S1:** This appendix details the spatial data sources and procedures used to reconstruct historical and current geographic distributions for threatened dryland mammal species included in this study.

Dryland regions were defined using Köppen major climate classifications, following standard delineations of arid and semi-arid environments in Australia (Appendix S1a: Bureau of Meteorology, 2025). These boundaries were used to determine whether species’ current or historical distributions met the dryland overlap threshold for inclusion in the study.


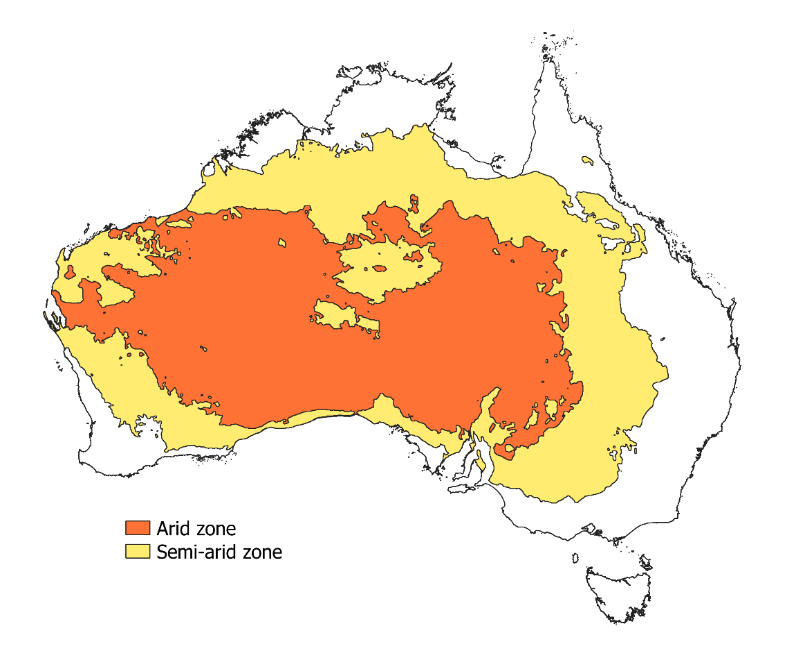


**(a)**

**Figure S1a:** We only included species with more than 10% of their historical or current distribution within the dryland regions of Australia (semi-arid and arid zone combined) as defined by the Köppen major climate classifications.

Historical species distributions were reconstructed to approximate geographic ranges at the time of European colonisation (circa 1788). Occurrence records were compiled from multiple sources, including the Atlas of Living Australia (ALA), the *Action Plan for Australian Mammals* (Woinarski et al., 2014), and consultation with multiple independent species experts. Records were screened to remove obvious spatial errors, misidentified or peripheral records inconsistent with expert knowledge, and all known translocated populations. Remaining high-confidence records were used to generate species-specific concave hull polygons using an alpha-hull approach (α = 0.4). This value was selected to balance exclusion of spurious outliers while retaining spatial coherence across discontinuous records.

To improve ecological realism and reduce artefacts from point-based range construction and low search effort in remote regions, alpha-hull polygons were intersected with Interim Biogeographic Regionalisation for Australia (IBRA) subregions. Subregions were retained if ≥20% of their area overlapped the alpha-hull polygon. In cases where IBRA subregions were spatially disjunct, only the portion intersecting the alpha-hull was retained. This approach ensured that reconstructed historical ranges reflected both known occurrence patterns and broader biogeographic structure, while avoiding over-extension into climatically or ecologically implausible areas.

Current species distributions were adapted from Marsh et al. (2022). All known conservation translocation sites were manually removed to ensure that current ranges reflected extant natural populations only. Distribution boundaries were further refined using post-2000 occurrence records to better align with recent observations and to prevent expansion beyond plausible historical limits. Adjustments were constrained by historical range boundaries to avoid incorporating areas unlikely to have supported populations prior to European colonisation.

Species were included if ≥10% of their current or historical distribution overlapped dryland regions. This criterion ensured that analyses focused on taxa with meaningful exposure and adaptation to arid or semi-arid climates. In addition to species-level assessments, two conservation-significant population units were included due to their notable conservation status or documented vulnerability to extreme heat. These were the Pilbara population of the orange leaf-nosed bat (*Rhinonicteris aurantia*), listed as vulnerable under the EPBC Act, and central Australian populations of the brushtail possum (*Trichosurus vulpecula*), which have experienced severe declines and likely local extirpation linked to extreme heat events. For these taxa, historical distributions encompassed the full species range, while current distributions were restricted to the focal population units. All spatial processing was conducted in QGIS v3.26.2 and R (RStudio Team, 2025). Geographic operations were implemented using the *sf* and *terra* packages, with all spatial layers projected to consistent coordinate reference systems prior to analysis.


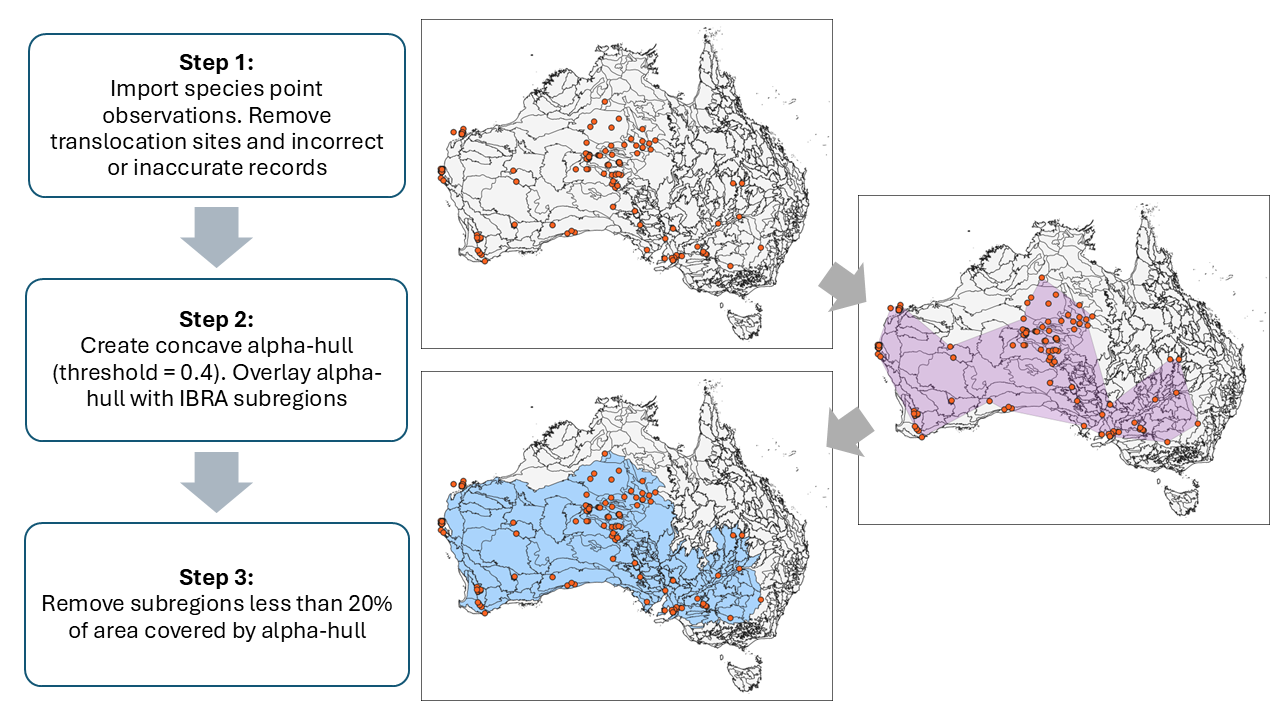


**(b)**

**Figure S1b**: An example workflow of the production of a historic distribution map using the boodie (*Bettongia lesueur*). Point observations are sourced from the Atlas of Living Australia and the Action Plan for Australian Mammals and then verified by independent experts.
