# Supplementary material for: Estimating the Impacts of Future Extreme Heat on Dryland Threatened Mammals: An Australian Case Study
Source: Glob Chang Biol. 2026 Apr 20;32(4):e70872. doi: 10.1111/gcb.70872 (PMC13094399; doi:10.1111/gcb.70872)
Supplement: Supplementary file 3 — Appendix S3: The PRISMA flow diagram for different phases of the systematic review. Boolean strings used to assess the literature for heat responses in the target species. Scientific name, recent synonyms and common names were chosen as stated by Baker and Gynther (2023). [file GCB-32-e70872-s005.docx]

**Appendix S3:** The PRISMA flow diagram for different phases of the systematic review. Boolean strings used to assess the literature for heat responses in the target species. Scientific name, recent synonyms, and common names were chosen as stated by Baker and Gynther (2023).


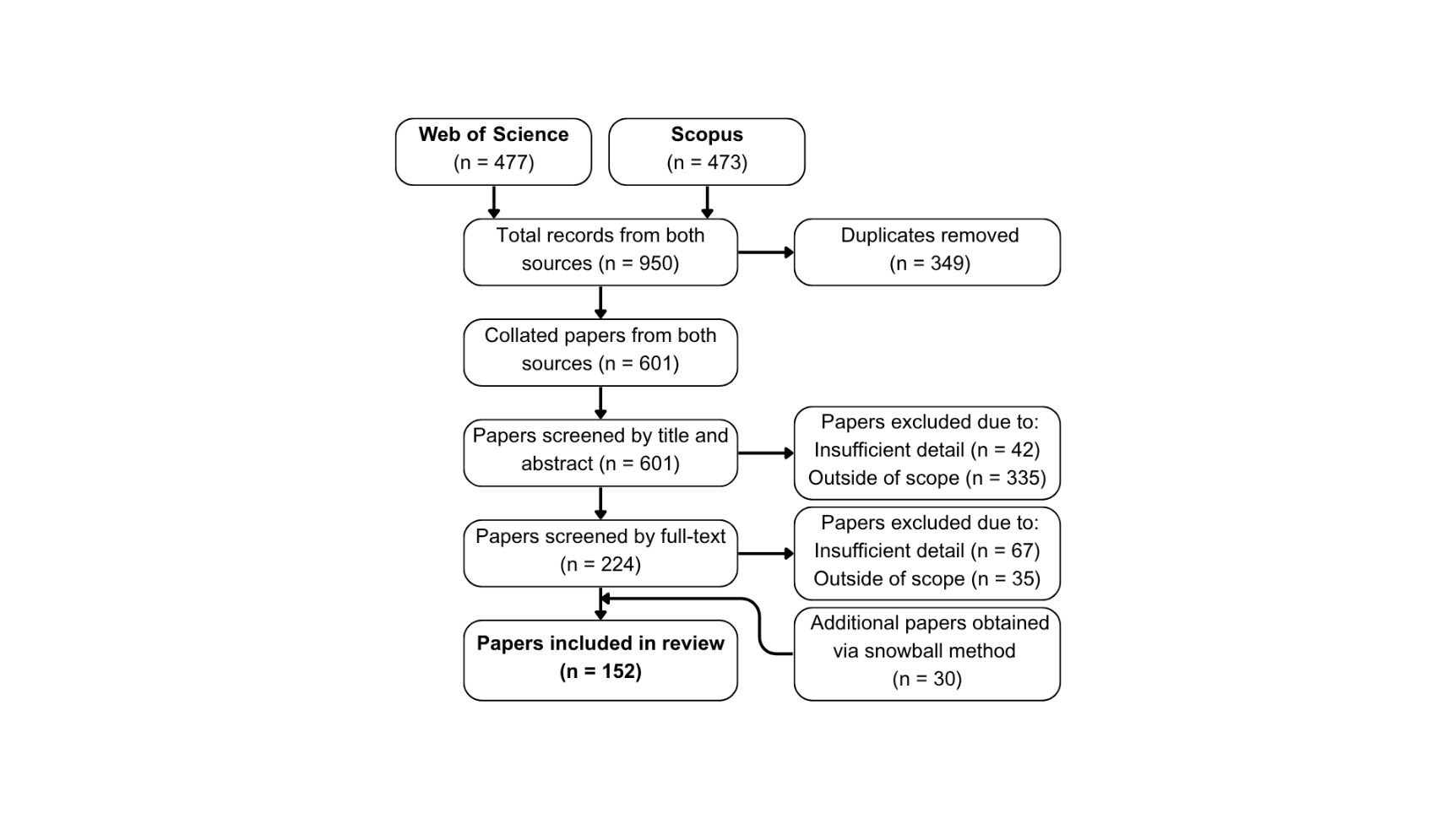


**Scopus**
TITLE-ABS-KEY("Bettongia lesueur" OR "Burrowing bettong" OR "Boodie" OR

"Bettongia penicillata" OR "Woylie" OR "Brush-tailed bettong" OR "Brush-tail bettong" OR

"Chalinolobus picatus" OR "Little pied bat" OR

"Dasycercus blythi" OR "Brush-tailed mulgara" OR “Ampurta” OR "Dasycercus byrnei" OR "Dasycercus cristicauda" OR "Dasycercus hillieri" OR

"Dasyuroides byrnei" OR "Kowari" OR

"Dasyurus geoffroii" OR "Western quoll" OR "Chuditch" OR "Dasyurinus geoffroii" OR

"Dasyurus hallucatus" OR "Northern quoll" OR "Satanellus hallucatus" OR

"Isoodon auratus" OR "Golden bandicoot" OR "Isoodon arnhemensis" OR "Isoodon barrowensis" OR

"Lagorchestes conspicillatus" OR "Spectacled hare-wallaby" OR

"Lagorchestes hirsutus" OR "Rufous hare-wallaby" OR

"Lagostrophus fasciatus" OR "Banded hare-wallaby" OR "Marnine" OR

"Lasiorhinus latifrons" OR "Southern hairy-nosed wombat" OR

"Leporillus conditor" OR "Greater stick-nest rat" OR "Leporillus jonesi" OR

"Macroderma gigas" OR "Ghost bat" OR

"Macrotis lagotis" OR "Greater bilby" OR "Thylacomys lagotis" OR

"Myrmecobius fasciatus" OR "Numbat" OR "Myrmecobius rufus" OR

"Notomys cervinus" OR "Fawn hopping mouse" OR

"Notomys fuscus" OR "Dusky hopping mouse" OR

"Onychogalea fraenata" OR "Bridled nailtail wallaby" OR "Onychogalea frenata" OR

"Perameles bougainville" OR "Western barred bandicoot" OR "Shark Bay bandicoot" OR

"Petrogale lateralis" OR "Black-flanked rock-wallaby" OR “Black-footed rock-wallaby” OR

"Petrogale purpureicollis" OR "Purple-necked rock-wallaby" OR

"Petrogale xanthopus" OR "Yellow-footed rock-wallaby" OR

"Phascogale calura" OR "Red-tailed phascogale" OR "Wambenger" OR

"Phascolarctos cinereus" OR "Koala" OR

"Pseudantechinus mimulus" OR "Carpentarian antechinus" OR

"Pseudomys australis" OR "Plains mouse" OR

"Pseudomys gouldii" OR "Gould's mouse" OR “Pseudomys fieldi” OR

"Pseudomys occidentalis" OR "Western mouse" OR "Gyomys occidentalis" OR

"Rhinonicteris aurantia" OR "Orange leaf-nosed bat" OR “Pilbara leaf-nosed bat” OR

"Setirostris eleryi" OR "Bristle-faced freetail bat" OR

"Sminthopsis douglasi" OR "Julia Creek dunnart" OR

"Sminthopsis psammophila" OR "Sandhill dunnart" OR

"Trichosurus vulpecula" OR "Common brush-tailed possum" OR "Trichosurus arnhemensis" OR "Trichosurus johnstonii" OR "Trichosurus fuliginosus" OR

"Zyzomys pedunculatus" OR "Central rock-rat")

AND ((TITLE-ABS-KEY ("heat" OR "temperature" OR "thermal" OR "climat*" OR "warming" OR “extreme” or “weather”))

**Web of Science**TS=("Bettongia lesueur" OR "Burrowing bettong" OR "Boodie" OR

"Bettongia penicillata" OR "Woylie" OR "Brush-tailed bettong" OR "Brush-tail bettong" OR

"Chalinolobus picatus" OR "Little pied bat" OR

"Dasycercus blythi" OR "Brush-tailed mulgara" OR "Dasycercus byrnei" OR "Dasycercus cristicauda" OR "Dasycercus hillieri" OR

"Dasyuroides byrnei" OR "Kowari" OR

"Dasyurus geoffroii" OR "Western quoll" OR "Chuditch" OR "Dasyurinus geoffroii" OR

"Dasyurus hallucatus" OR "Northern quoll" OR "Satanellus hallucatus" OR

"Isoodon auratus" OR "Golden bandicoot" OR "Isoodon arnhemensis" OR "Isoodon barrowensis" OR

"Lagorchestes conspicillatus" OR "Spectacled hare-wallaby" OR

"Lagorchestes hirsutus" OR "Rufous hare-wallaby" OR

"Lagostrophus fasciatus" OR "Banded hare-wallaby" OR "Marnine" OR

"Lasiorhinus latifrons" OR "Southern hairy-nosed wombat" OR

"Lasiorhinus krefftii" OR "Northern hairy-nosed wombat" OR

"Leporillus conditor" OR "Greater stick-nest rat" OR "Leporillus jonesi" OR

"Macroderma gigas" OR "Ghost bat" OR

"Macrotis lagotis" OR "Greater bilby" OR "Thylacomys lagotis" OR

"Myrmecobius fasciatus" OR "Numbat" OR "Myrmecobius rufus" OR

"Notomys cervinus" OR "Fawn hopping mouse" OR

"Notomys fuscus" OR "Dusky hopping mouse" OR

"Onychogalea fraenata" OR "Bridled nailtail wallaby" OR "Onychogalea frenata" OR

"Perameles bougainville" OR "Western barred bandicoot" OR "Shark Bay bandicoot" OR

"Petrogale lateralis" OR "Black-flanked rock-wallaby" OR “Black-footed rock-wallaby” OR

"Petrogale purpureicollis" OR "Purple-necked rock-wallaby" OR

"Petrogale xanthopus" OR "Yellow-footed rock-wallaby" OR

"Phascogale calura" OR "Red-tailed phascogale" OR "Wambenger" OR

"Phascolarctos cinereus" OR "Koala" OR

"Pseudantechinus mimulus" OR "Carpentarian antechinus" OR

"Pseudomys australis" OR "Plains mouse" OR

"Pseudomys gouldii" OR "Gould's mouse" OR “Pseudomys fieldi” OR

"Pseudomys occidentalis" OR "Western mouse" OR "Gyomys occidentalis" OR

"Rhinonicteris aurantia" OR "Orange leaf-nosed bat" OR “Pilbara leaf-nosed bat” OR

"Setirostris eleryi" OR "Bristle-faced freetail bat" OR

"Sminthopsis douglasi" OR "Julia Creek dunnart" OR

"Sminthopsis psammophila" OR "Sandhill dunnart" OR

"Trichosurus vulpecula" OR "Common brush-tailed possum" OR "Trichosurus arnhemensis" OR "Trichosurus johnstonii" OR "Trichosurus fuliginosus" OR

"Zyzomys pedunculatus" OR "Central rock-rat")
AND TS=("heat" OR "temperature" OR "thermal" OR "climat*" OR "warming" OR "extreme" OR "weather")
