# Supplementary material for: Estimating the Impacts of Future Extreme Heat on Dryland Threatened Mammals: An Australian Case Study
Source: Glob Chang Biol. 2026 Apr 20;32(4):e70872. doi: 10.1111/gcb.70872 (PMC13094399; doi:10.1111/gcb.70872)

**Appendix S4:** *All climate scenarios included in Figure* 3. The percentage of a species’ projected future climate envelope is compared with the percentage overlapping the historical climate envelope. Each species was tested across 15 future climate scenarios for 2041-2070 (five models for each of ssp126, ssp370, and ssp585, indicated by colour). Point size is proportion to the species current distribution size on a logarithmic scale. The dotted 1:1 line indicates equal overlap with current and historical climate envelopes.
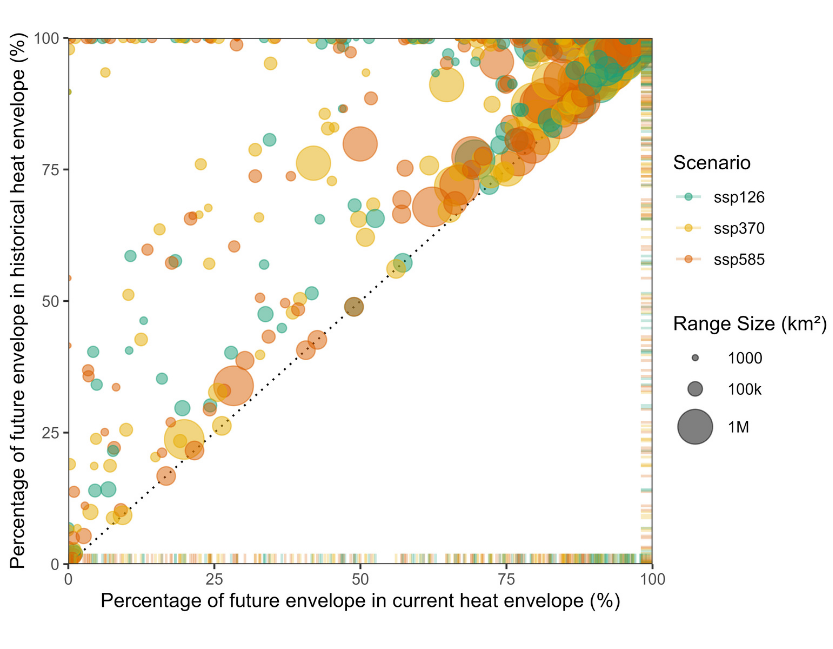


*Comprehensively labelled version of Figure 3 with all species included*. Colour indicates IUCN status and point size is representative of the species’ current range size on a logarithmic scale. Species in the upper left are moderate risk (limited overlap with current but not historical envelopes); those in the upper right are low risk.


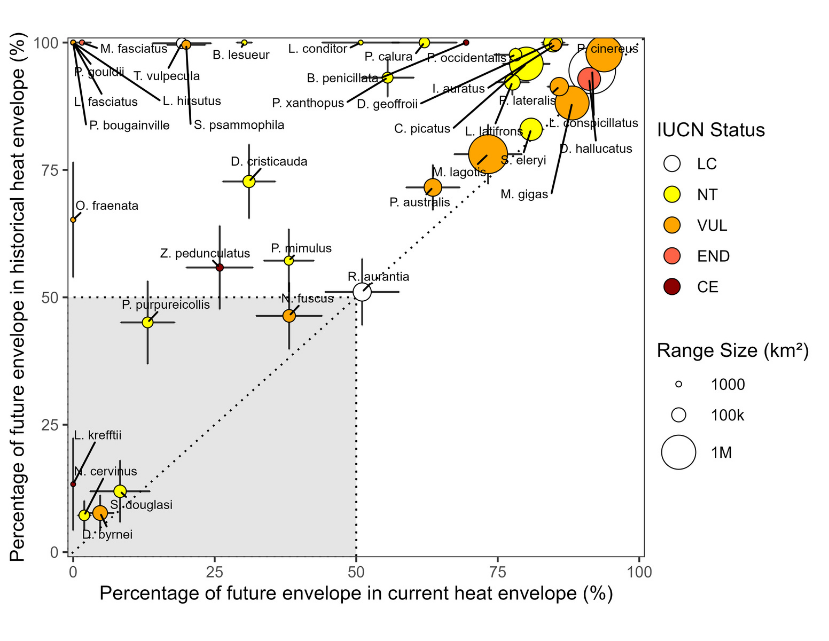

Supplement: Supplementary file 4 — Appendix S4: All individual climate projections and comprehensively labelled version of Figure 3. An assessment of future novel heat load of dryland threatened mammals. The percentage of a species' future range within their current climate envelope is compared to the percentage of a species' future range compared to their historic climate envelope. Colour indicates shared socioeconomic pathway in (a) and IUCN status in (b) and point size is representative of the species' current range on a logarithmic scale. [file GCB-32-e70872-s003.docx]
