# Supplementary material for: Estimating the Impacts of Future Extreme Heat on Dryland Threatened Mammals: An Australian Case Study
Source: Glob Chang Biol. 2026 Apr 20;32(4):e70872. doi: 10.1111/gcb.70872 (PMC13094399; doi:10.1111/gcb.70872)
Supplement: Supplementary file 5 — Appendix S5: Individual species profiles of heat load projections for all 36 threatened dryland mammals, listed alphabetically. [file GCB-32-e70872-s002.docx]

**Appendix S5:** Species Heat Profiles

Heat projections for all 36 threatened arid zone mammals. Species names and IUCN or EPBC status are given, dependent on which is higher risk. **(a)**A density plot showing the maximum temperature of the warmest month (bioclim BIO5 parameter) across a species’ historic (dark blue) and current range (light blue) under current climate (1981-2010). The most mild and most extreme future climate projections (2041-2070) are shown for the current range. **(b)** BIO5 projections at each translocation site for the species under different climate scenarios: current climate (blue diamond), most mild future projection (orange point), and most extreme future projection (red point). **(c)** The area within the species’ historic distribution where the BIO5 parameter will remain below the current highest temperature experienced in the species’ historic range under all climate scenarios (green; highly suitable), some climate scenarios (orange; potentially suitable), and no climate scenarios (red; not suitable) by 2041-2070. Areas remaining below the species historic thermal maximum (green; highly suitable) likely indicate refugia or potential reintroduction sites for the species provided suitable habitat has persisted and other climatic variables remain within the species’ preferences. The species’ current range is indicated by a dashed line or black arrow and translocation sites are indicated by a blue diamond.

**Annotated Key –** Numbat (*Myrmecobius fasciatus*)

**
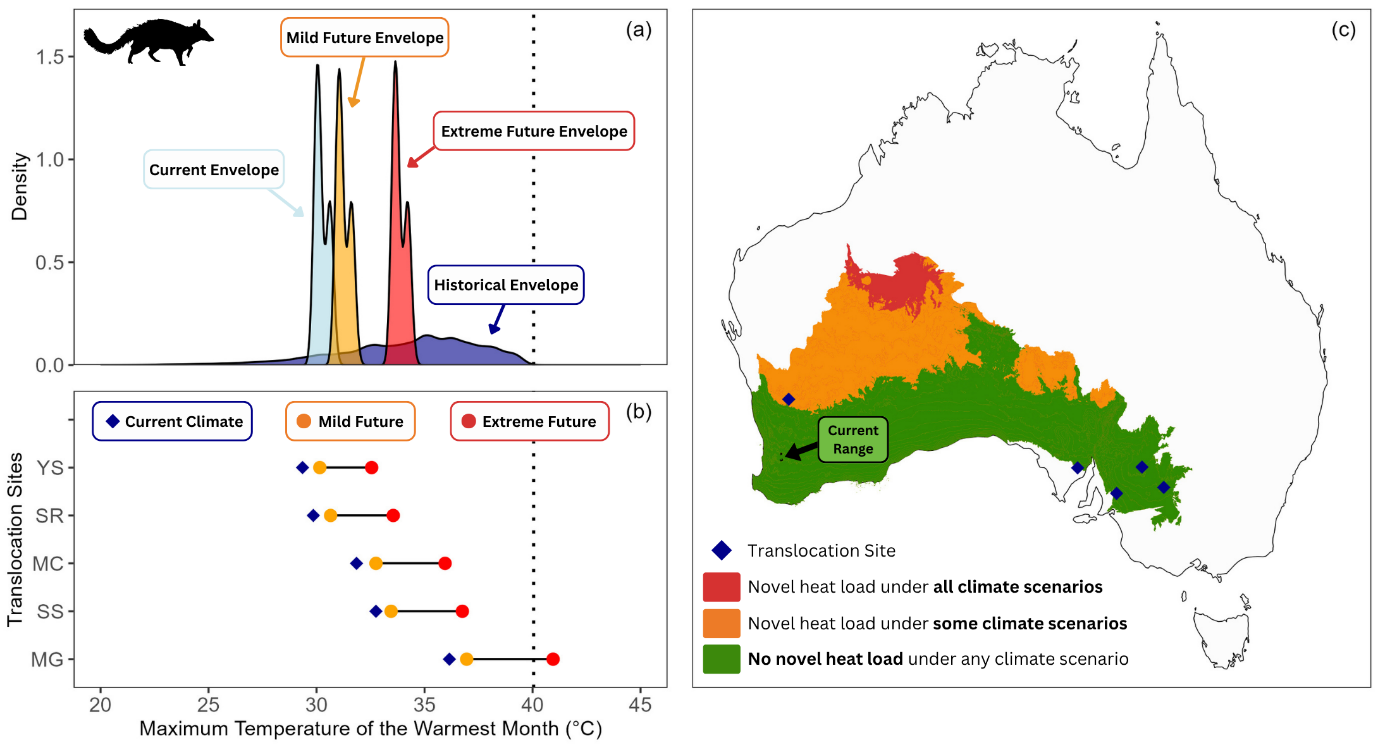
**

**Table of Contents**

| 1. *Bettongia lesueur* 2. *Bettongia penicillata* 3. *Chalinolobus picatus* 4. *Dasycercus cristicauda* 5. *Dasyuroides byrnei* 6. *Dasyurus geoffroii* 7. *Dasyurus hallucatus* 8. *Isoodon auratus* 9. *Lagorchestes conspicillatus* 10. *Lagorchestes hirsutus* 11. *Lagostrophus fasciatus* 12. *Lasiorhinus krefftii* 13. *Lasiorhinus latifrons* 14. *Leporillus conditor* 15. *Macroderma gigas* 16. *Macrotis lagotis* 17. *Myrmecobius fasciatus* 18. *Notomys cervinus* | 1. *Notomys fuscus* 2. *Onychogalea fraenata* 3. *Perameles bougainville* 4. *Petrogale lateralis* 5. *Petrogale purpureicollis* 6. *Petrogale xanthopus* 7. *Phascogale calura* 8. *Phascolarctos cinereus* 9. *Pseudantechinus mimulus* 10. *Pseudomys australis* 11. *Pseudomys gouldii* 12. *Pseudomys occidentalis* 13. *Rhinonicteris aurantia* (Pilbara form) 14. *Setirostris eleryi* 15. *Sminthopsis douglasi* 16. *Sminthopsis psammophila* 17. *Trichosurus vulpecula* (Central Aus) 18. *Zyzomys pedunculatus* |
| --- | --- |

*
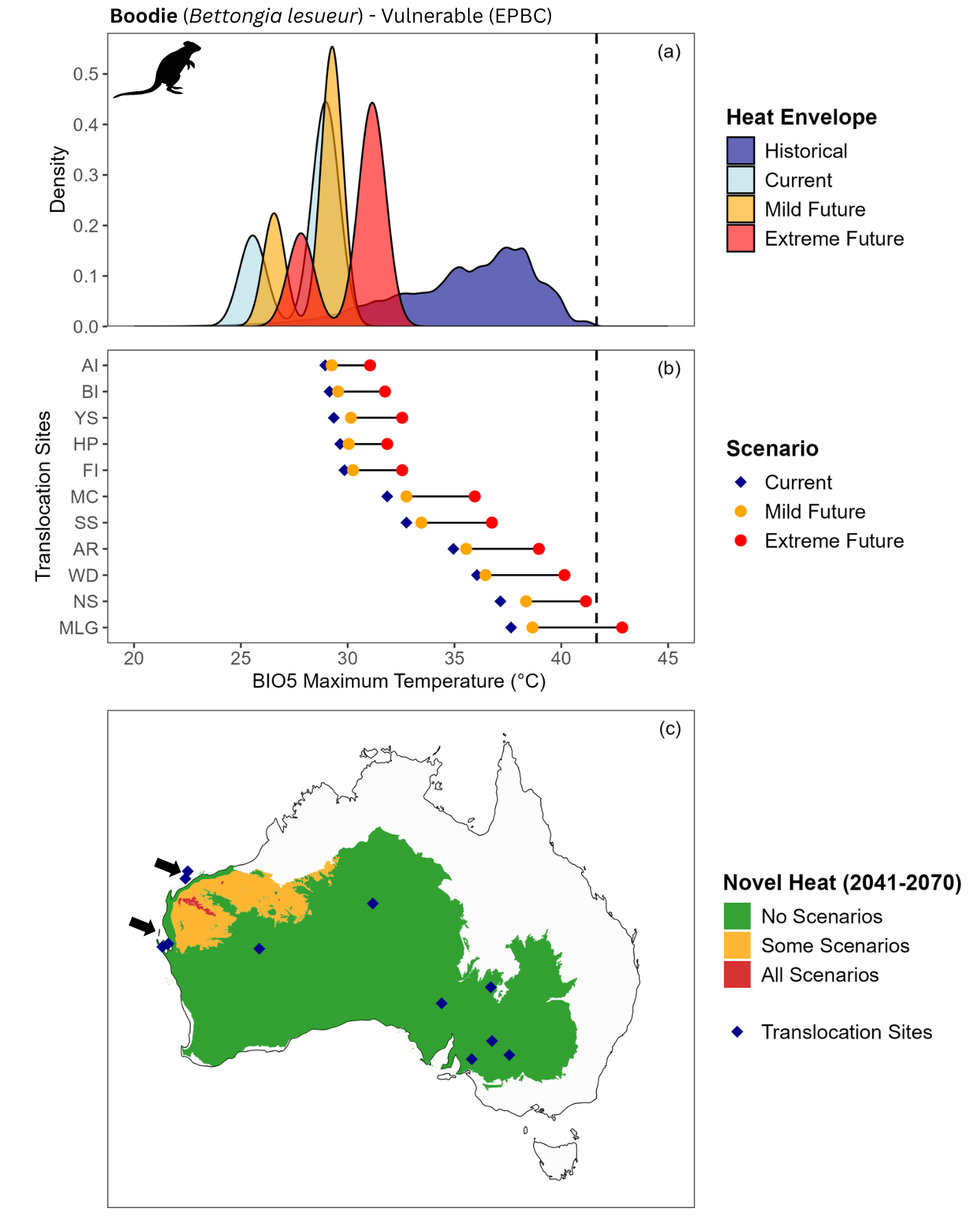

Black arrows indicate current range.*

**Translocation sites:** AI = Alpha Island, BI = Boodie Island, YS = Yookamurra Sanctuary, HP = Heirisson Prong, FI = Faure Island, MC = Mallee Cliffs NP, SS = Scotia Sanctuary, AR = Arid Recovery, WD = Wild Deserts, NS = Newhaven Sanctuary, and MLG = Matuwa (Lorna Glen).

*
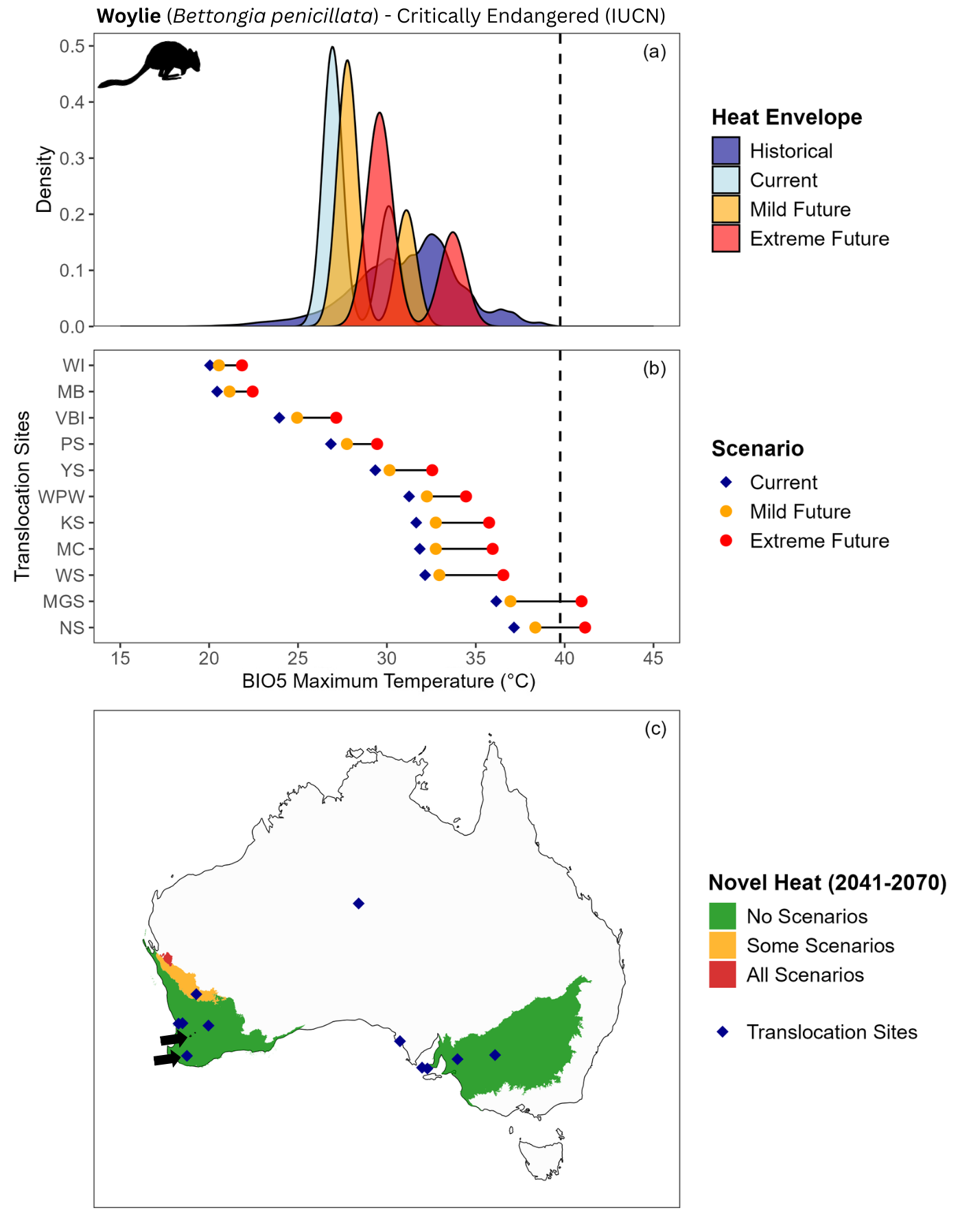
Black arrows indicate current range.*

**Translocation sites:** WI = Wedge Island, MB = Marna Bangarra, VBI = Venus Bay Island, PS = Perup Sanctuary, YS = Yookamurra Sanctuary, WPW = Whiteman Park Woodland Reserve, KS = Karakamia Sanctuary, MC = Mallee Cliffs NP, WS = Wadderin Sanctuary, MGS = Mt Gibson Sanctuary, and NS = Newhaven Sanctuary.


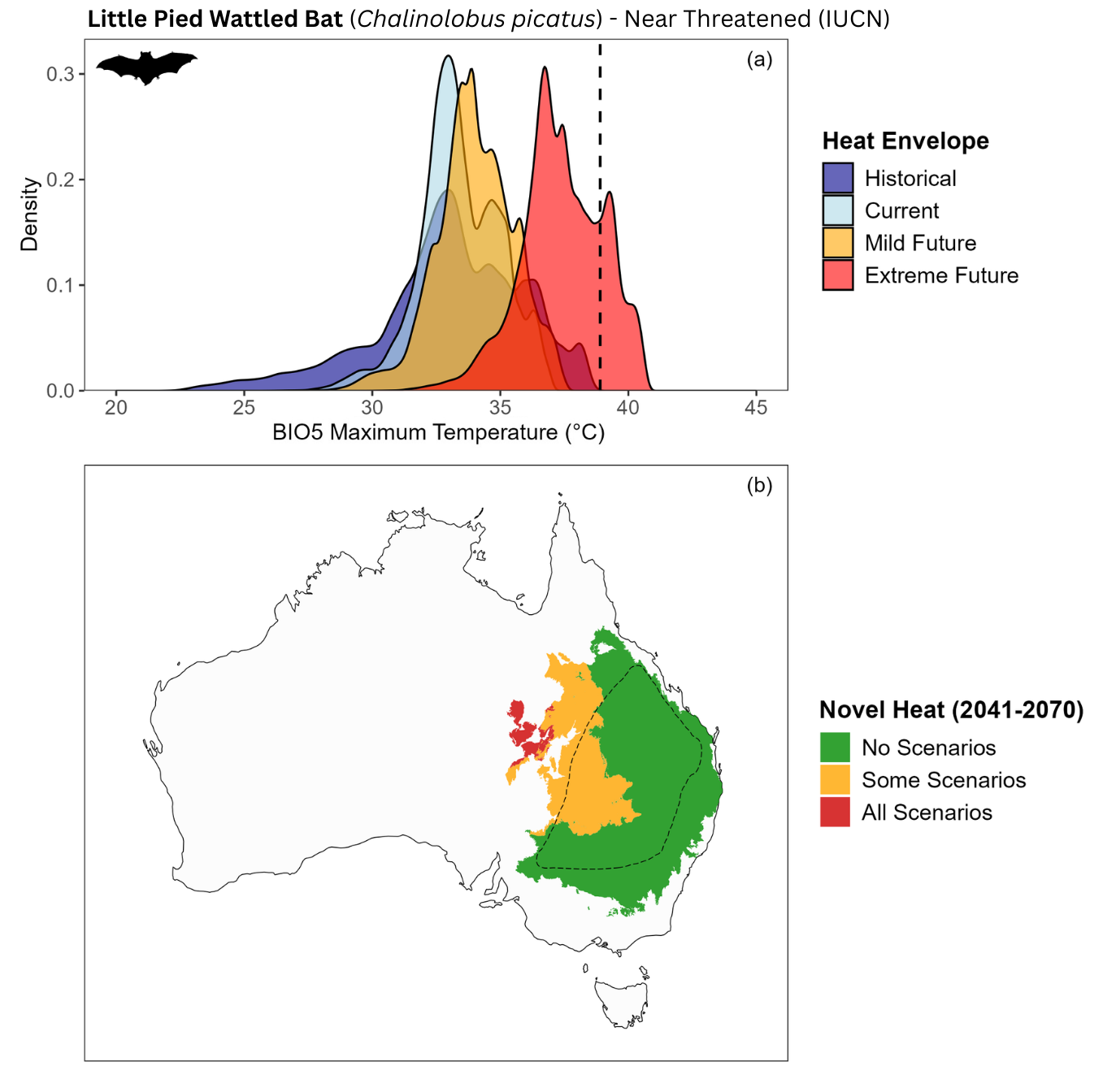

*Dotted line indicates current range.*


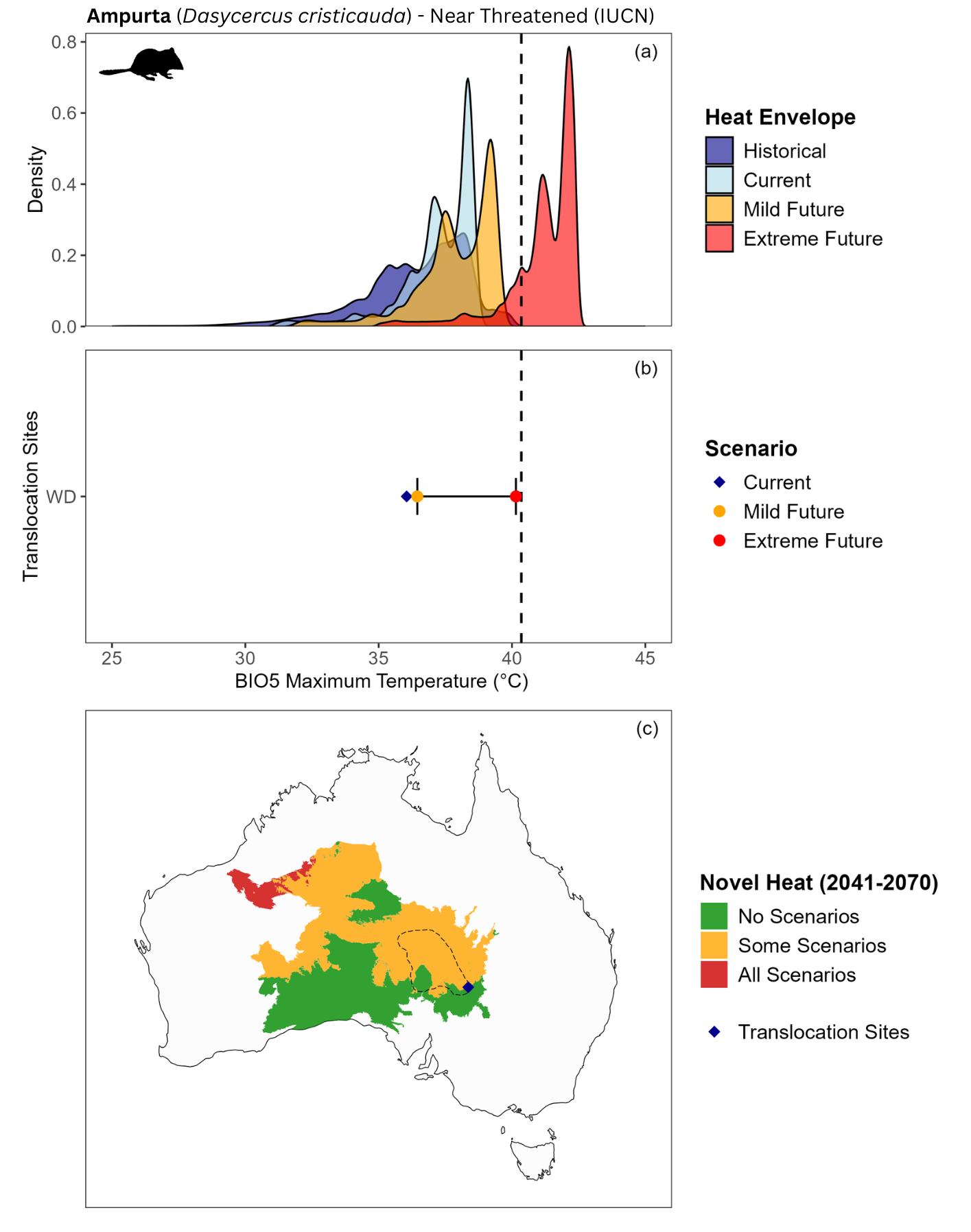

*Dotted line indicates current range.*

**Translocation sites:** WD = Wild Deserts.


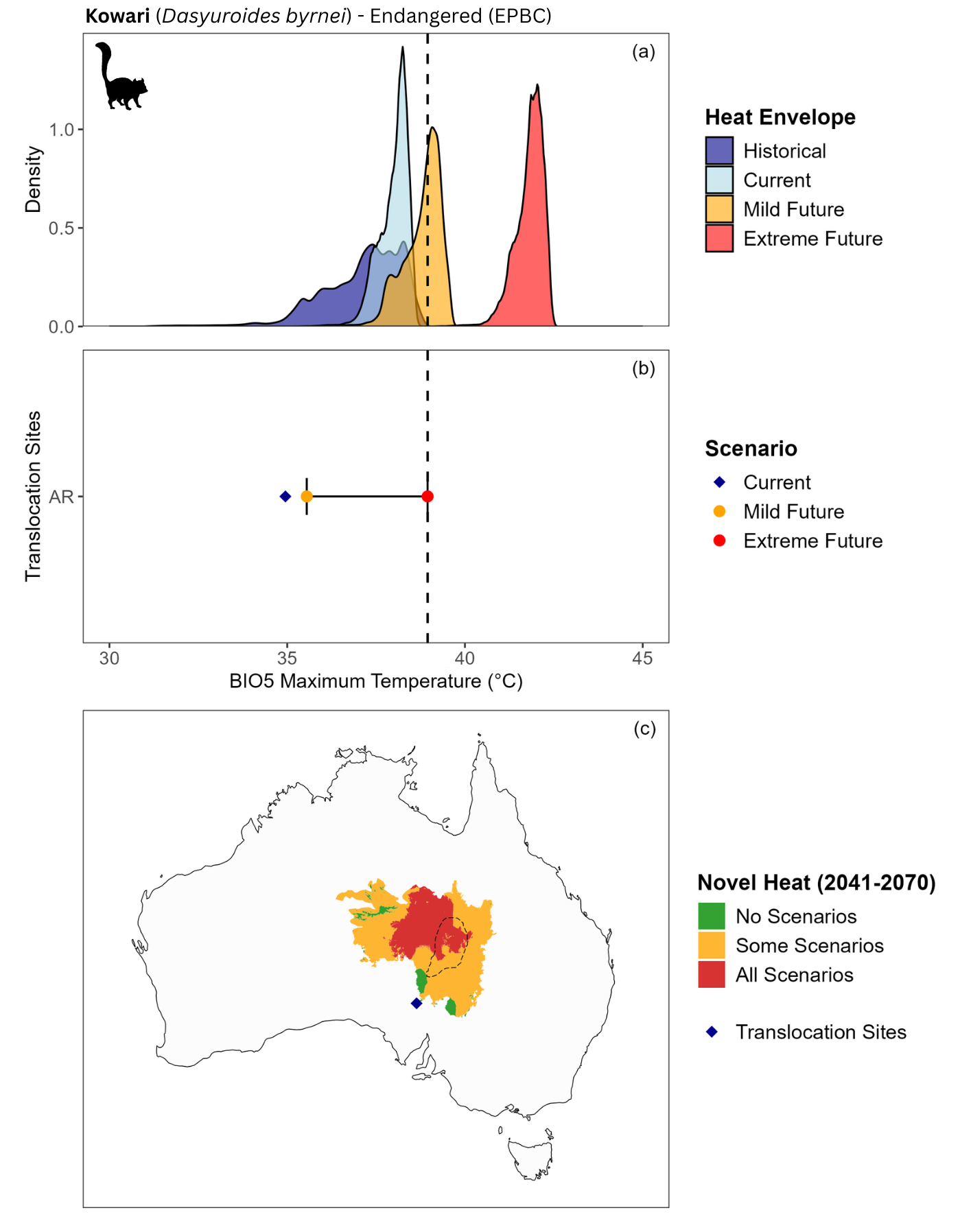

*Dotted line indicates current range.*

**Translocation sites:** AR = Arid Recovery.


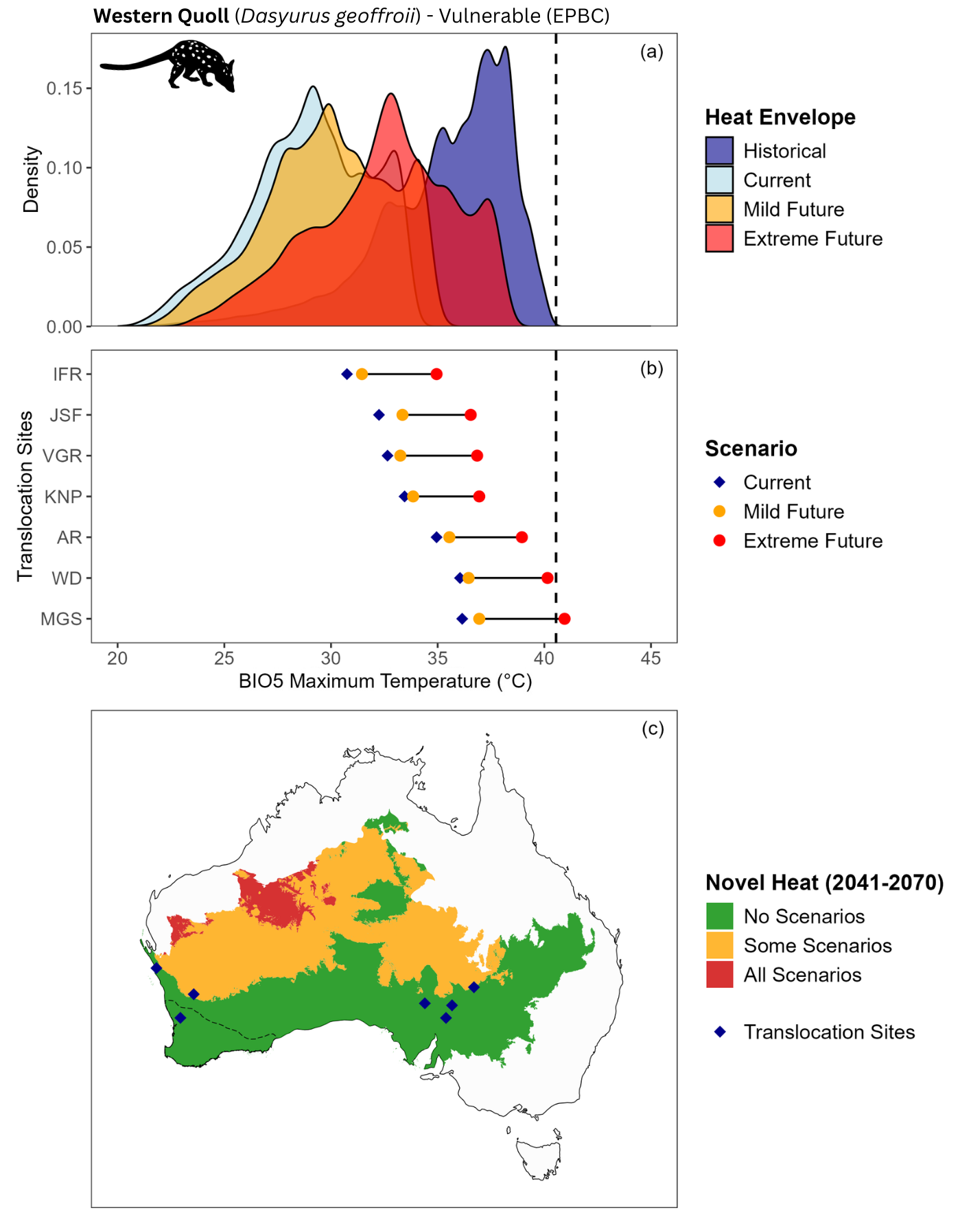

*Dotted line indicates current range.*

**Translocation sites:** IFR = Ikara-Flinders Ranges National Park, Julimar State Forest, Vulkathunha-Gammon Ranges National Park, KNP = Kalbarri National Park, AR = Arid Recovery, and WD = Wild Deserts.


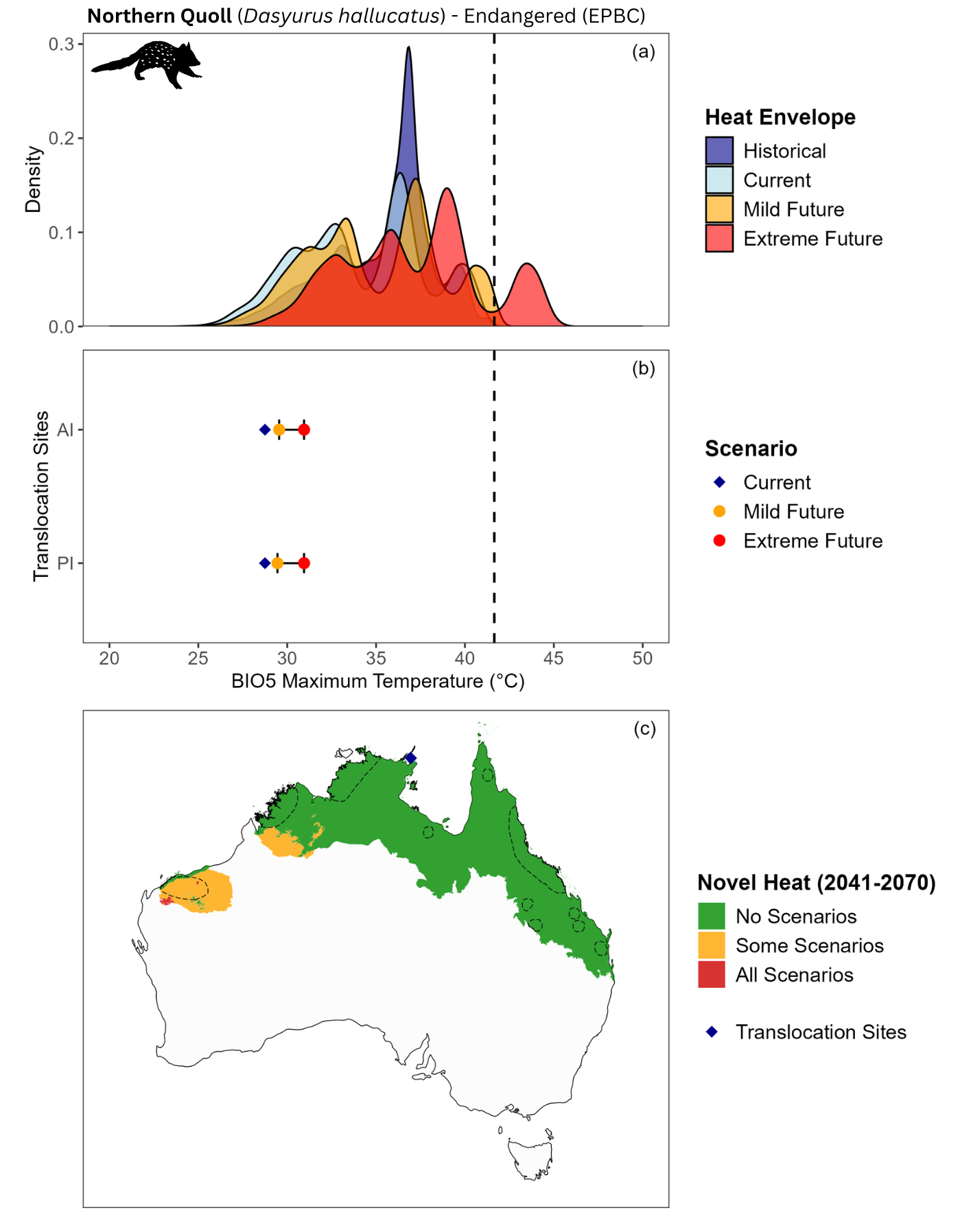

*Dotted line indicates current range.*

**Translocation sites:** AI = Astell Island and PI = Pobasso Island.


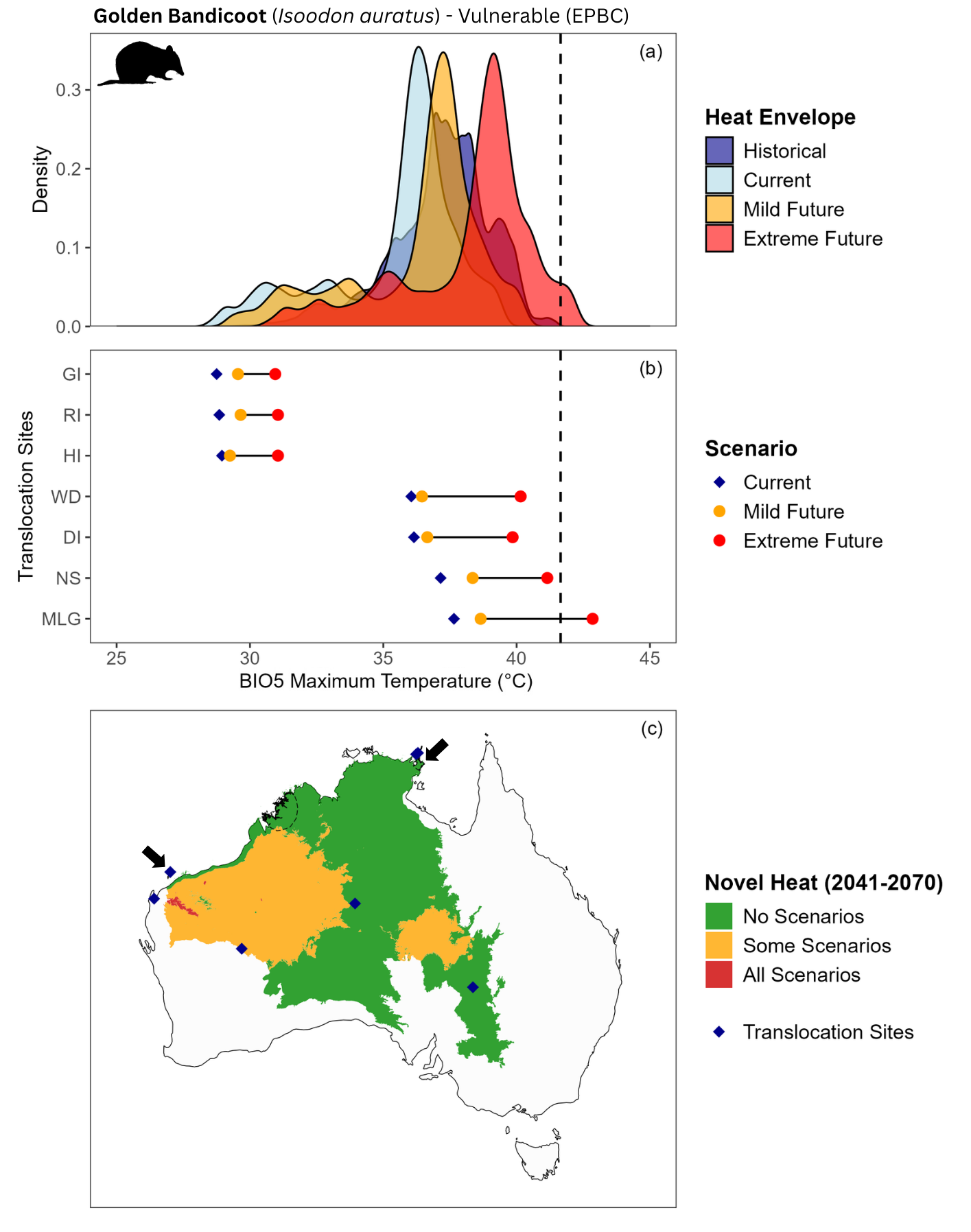

*Dotted line and black arrows indicate current range.*

**Translocation sites:** GI = Guluwuru Island, RI = Raragala Island, HI = Hermite Island, WD = Wild Deserts, DI = Doole Island, NS = Newhaven Sanctuary, and MLG = Matuwa (Lorna Glen).


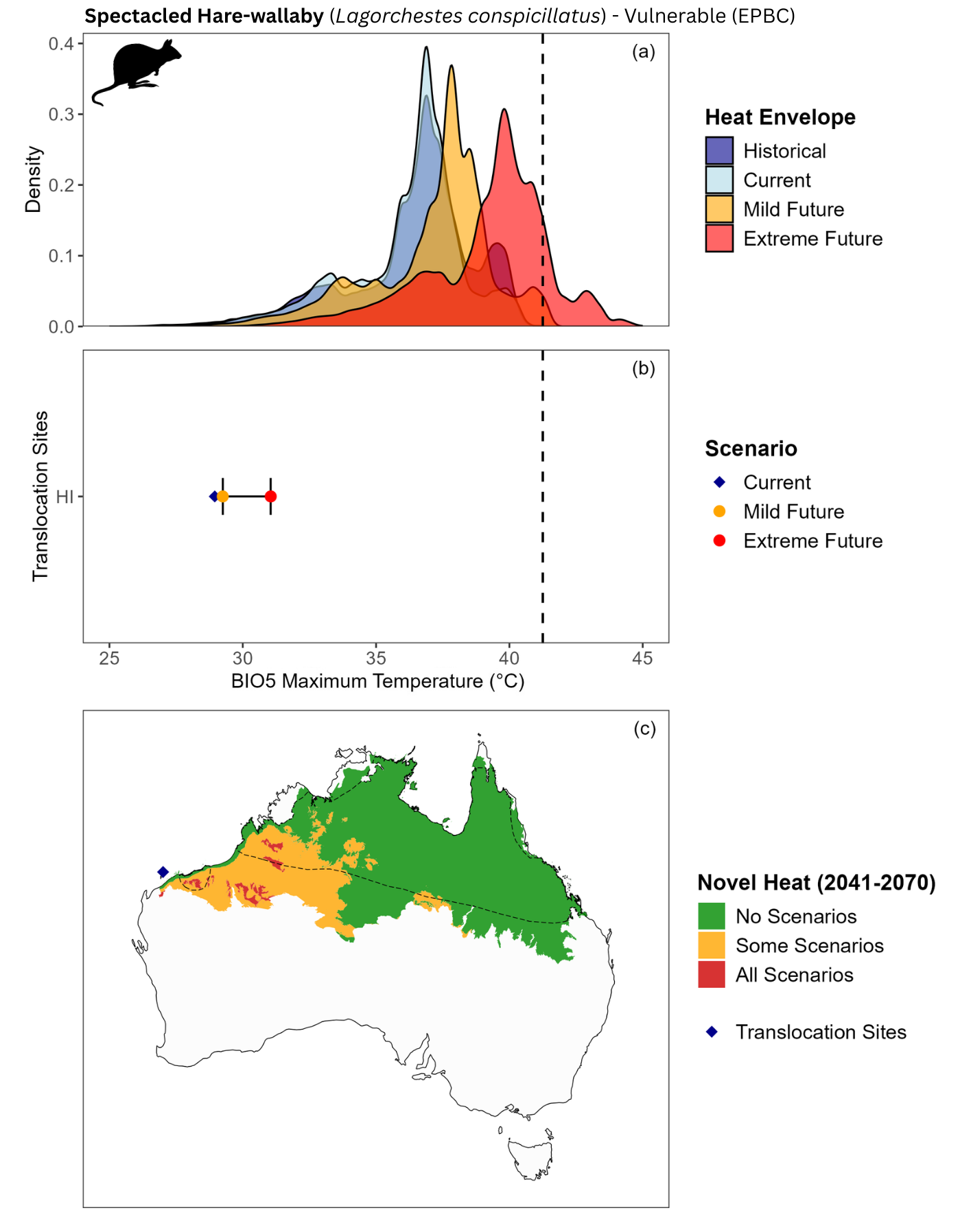

*Dotted line indicates current range.*

**Translocation sites:** HI = Hermite Island.


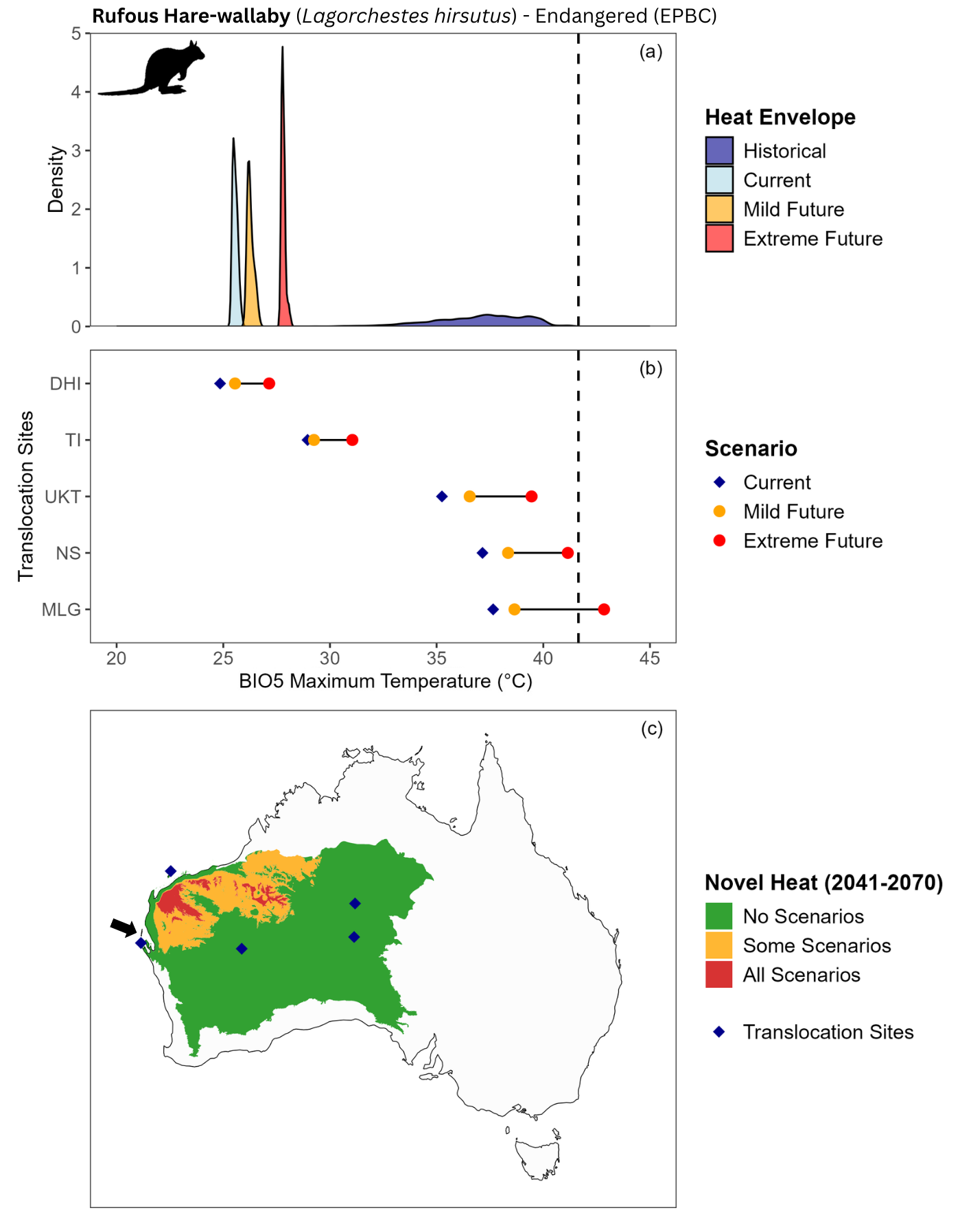

*Black arrows indicate current range.*

**Translocation sites:** DHI = Dirk Hartog Island, TI = Trimouille Island, UKT = Uluru-Kata Tjuta Pen, NS = Newhaven Sanctuary, and MLG = Matuwa (Lorna Glen).


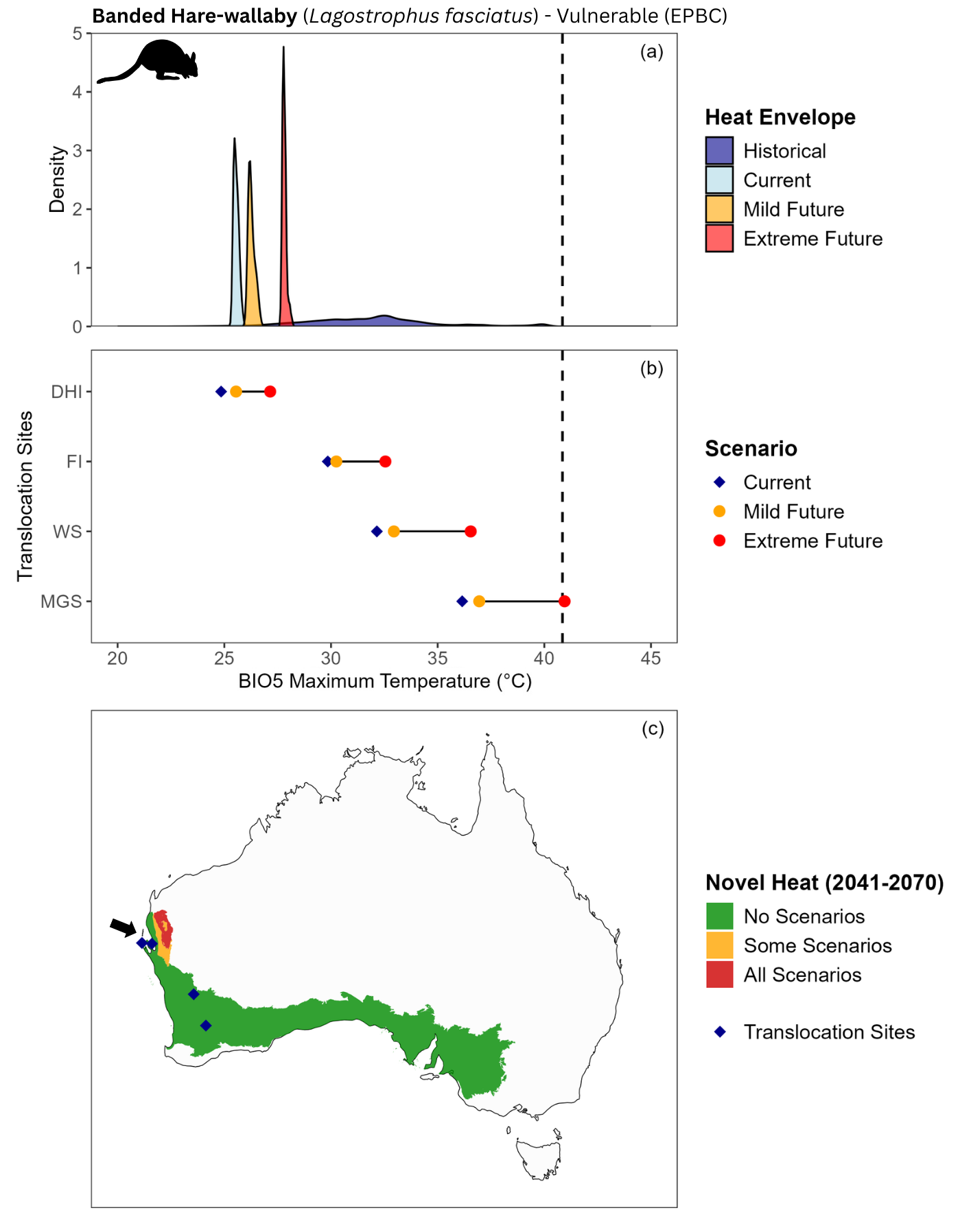

*Black arrows indicate current range.*

**Translocation sites:** DHI = Dirk Hartog Island, FI = Faure Island, WS = Wadderin Sanctuary, and MGS = Mt Gibson Sanctuary.


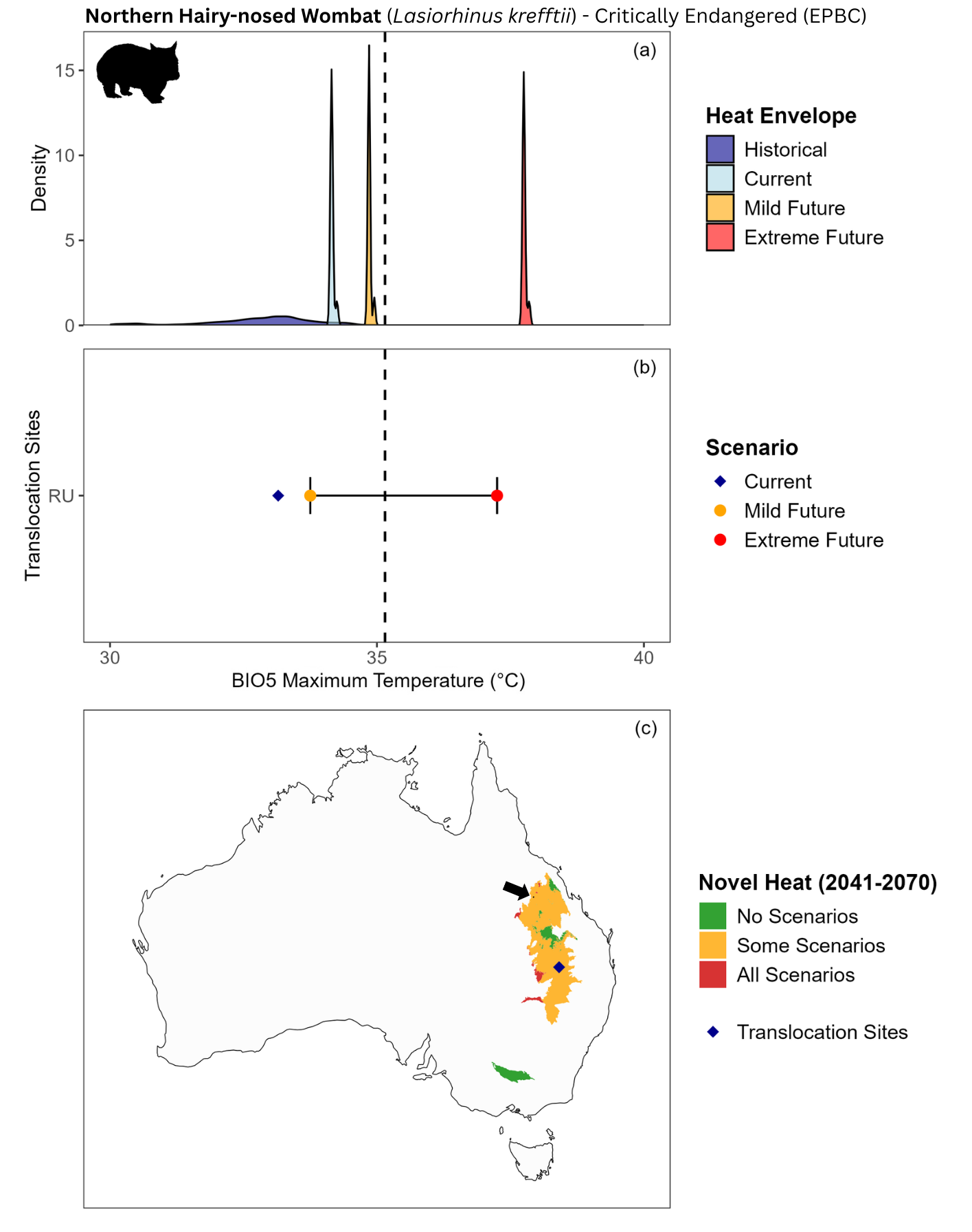

*Black arrows indicate current range.*

**Translocation sites:** Richard Underwood Nature Refuge = RU.


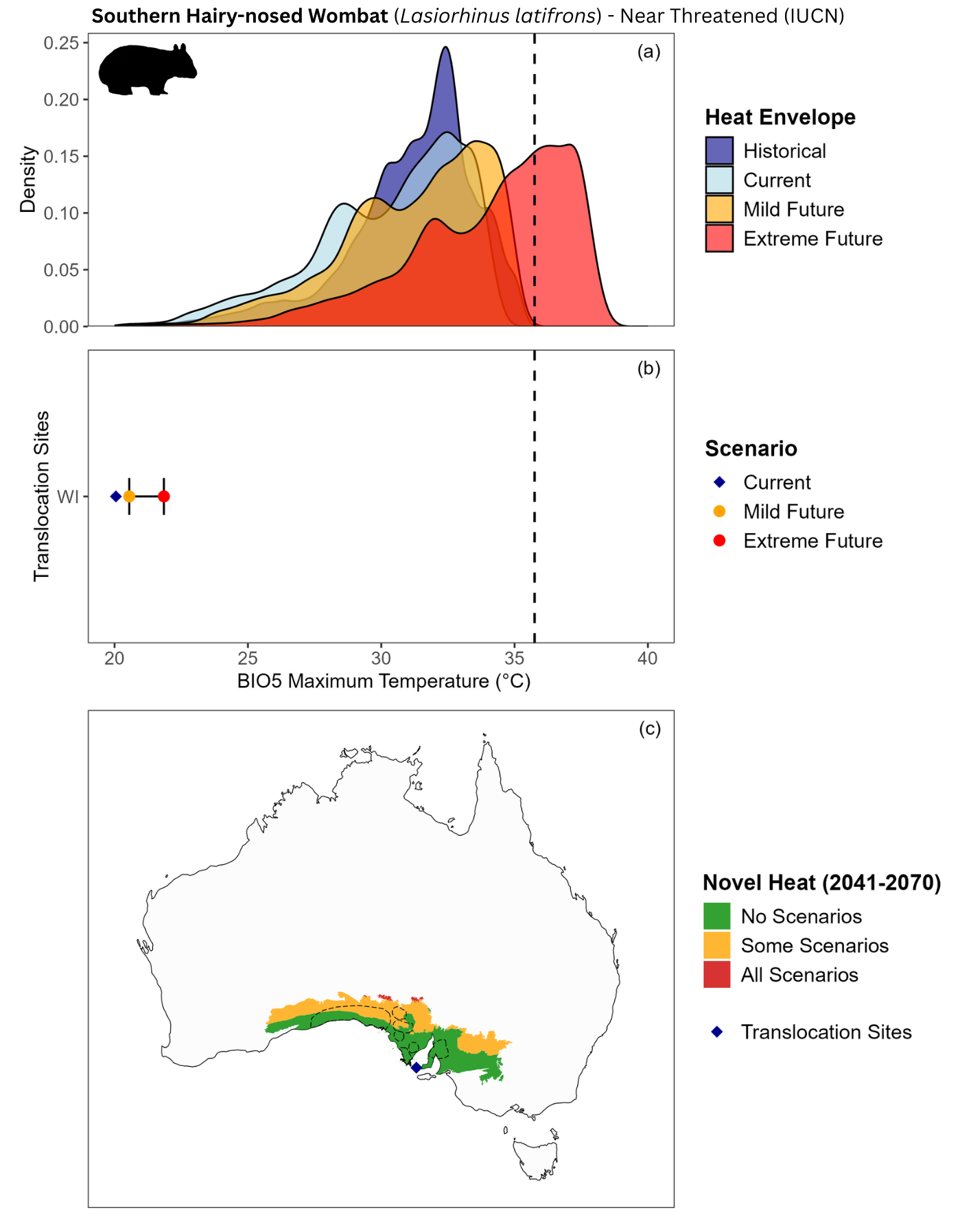

*Dotted line indicates current range.*

**Translocation sites:** WI = Wedge Island.


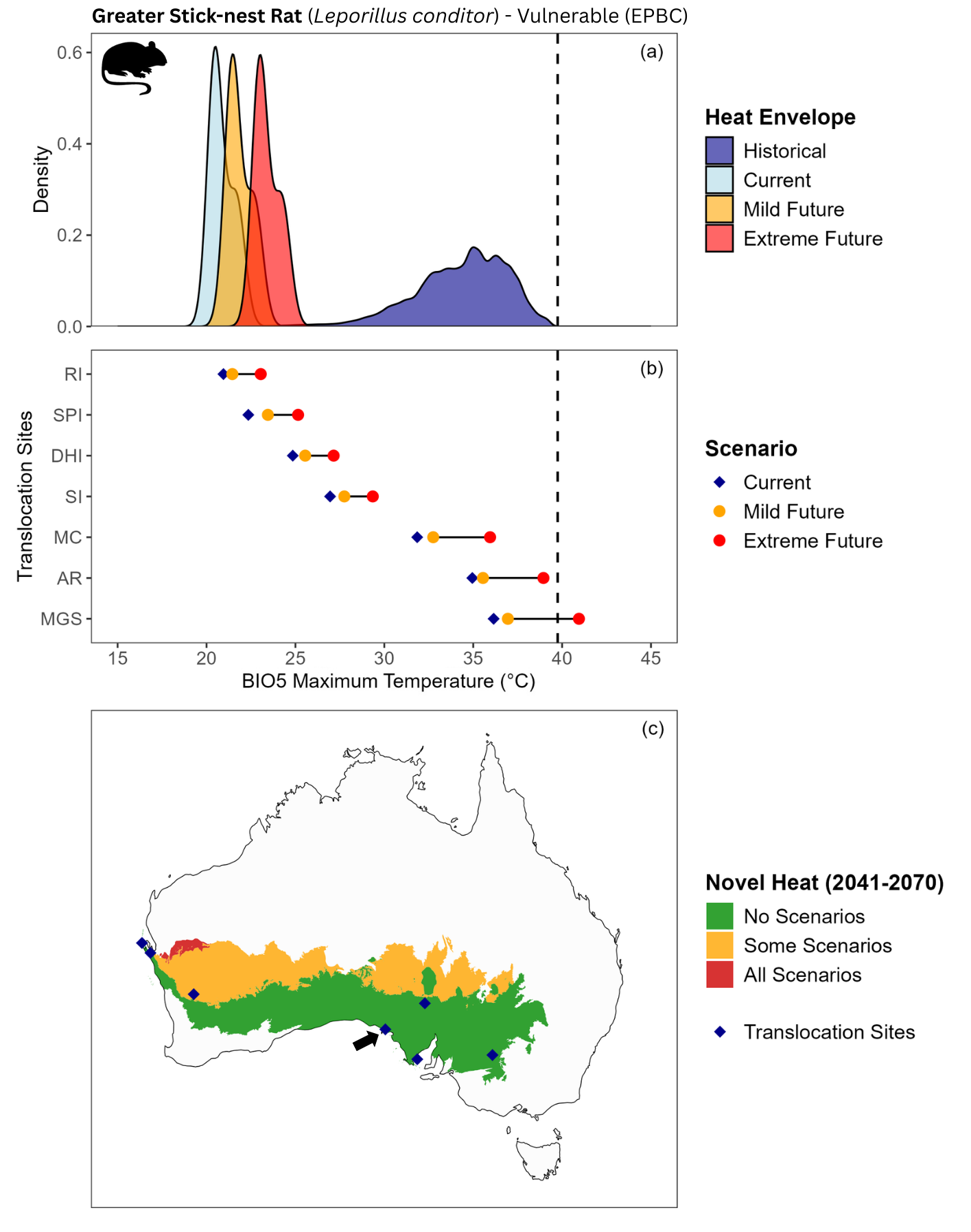
*Black arrow indicates current range. Arid Recovery was included as a translocation site despite the species’ recent extirpation from the site, given the former long-term success of the reintroduction.*

**Translocation sites:** RI = Reevesby Island, SPI = St Peter Island, DHI = Dirk Hartog Island, SI = Salutation Island, MC = Mallee Cliffs NP, AR = Arid Recovery, and MGS = Mt Gibson Sanctuary.


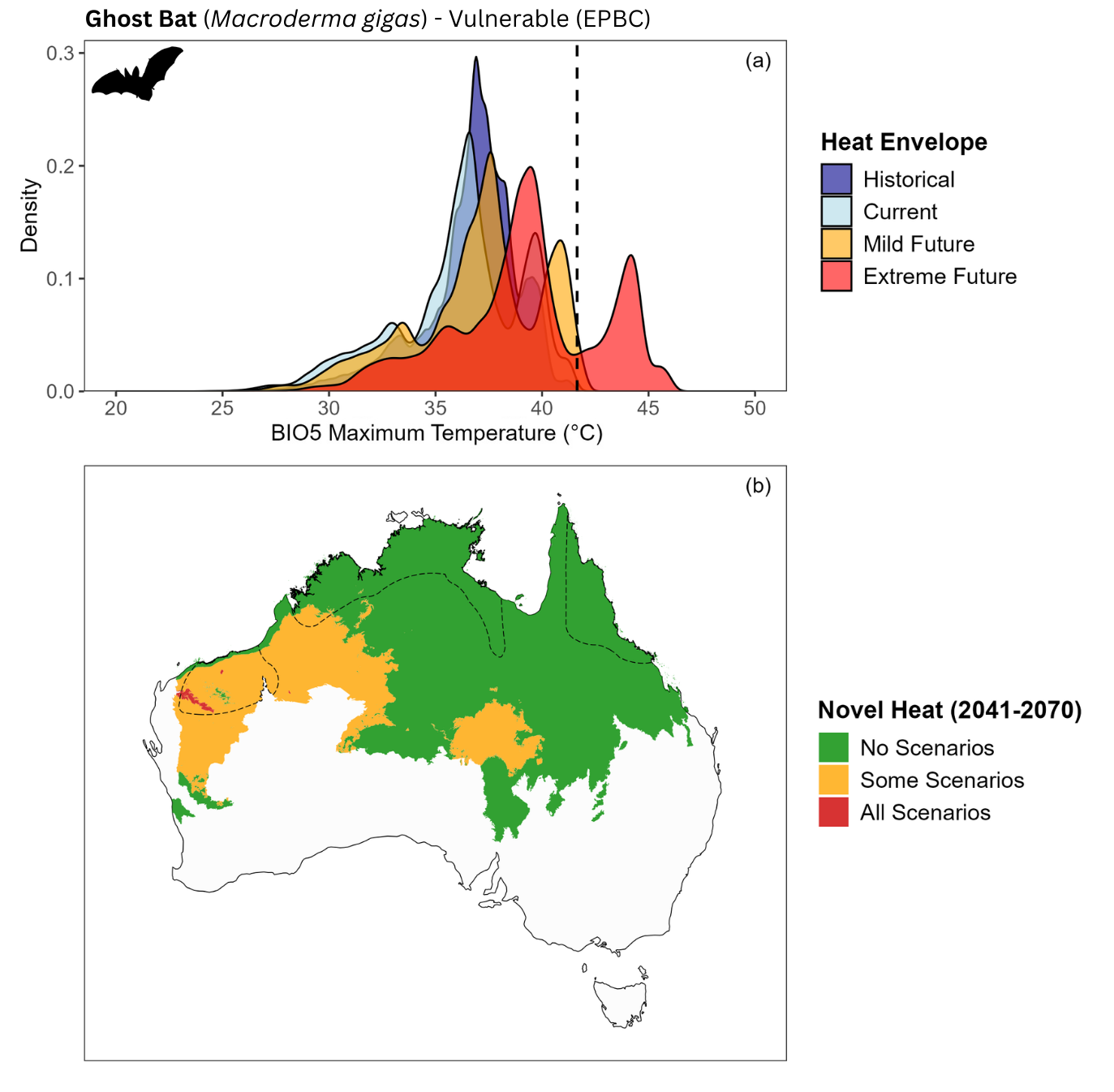
*Dotted line indicates current range.*


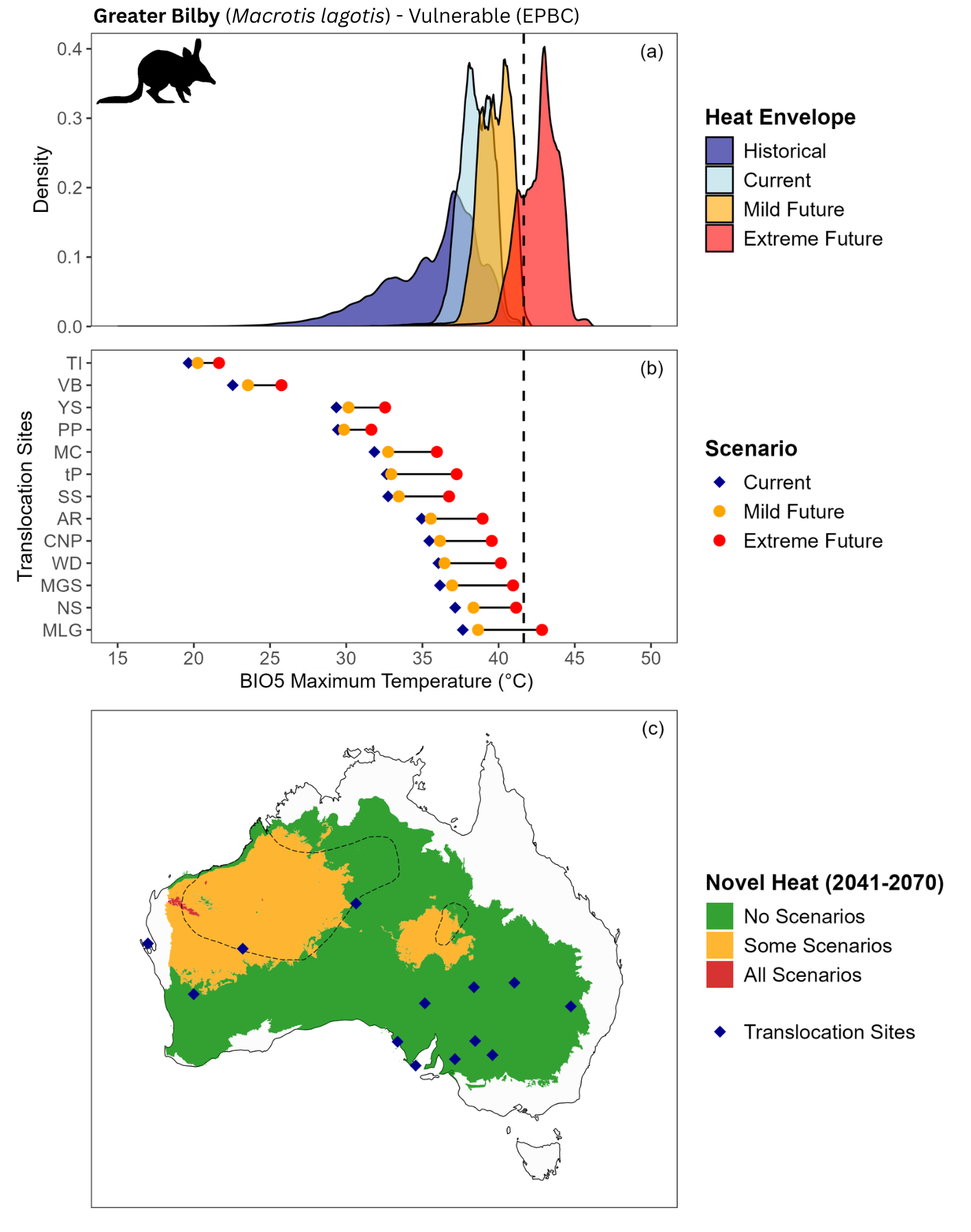

*Dotted line indicates current range.*

**Translocation sites:** TI = Thistle Island, VB = Venus Bay Conservation Park, YS = Yookamurra Sanctuary, PP = Peron Sanctuary, MC = Mallee Cliffs NP, tP = The Pilliga, SS = Scotia Sanctuary, AR = Arid Recovery, WD = Wild Deserts, MGS = Mt Gibson Sanctuary, NS = Newhaven Sanctuary, and MLG = Matuwa (Lorna Glen).


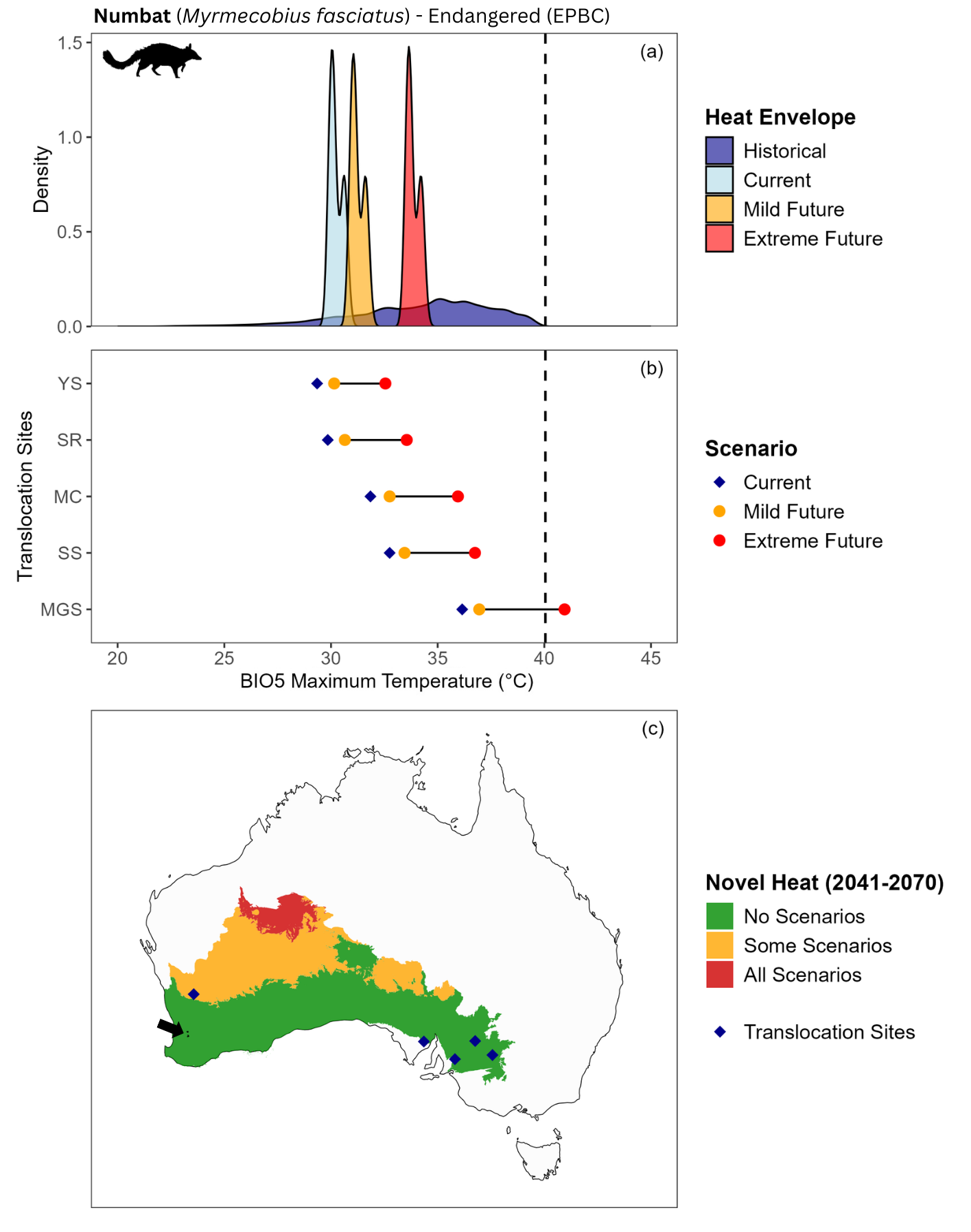

*Black arrow indicates current range.*

**Translocation sites:** YS = Yookamurra Sanctuary, SR = Secret Rocks Mallee Refuge, MC = Mallee Cliffs NP, SS = Scotia Sanctuary, and MGS = Mt Gibson Sanctuary.

*
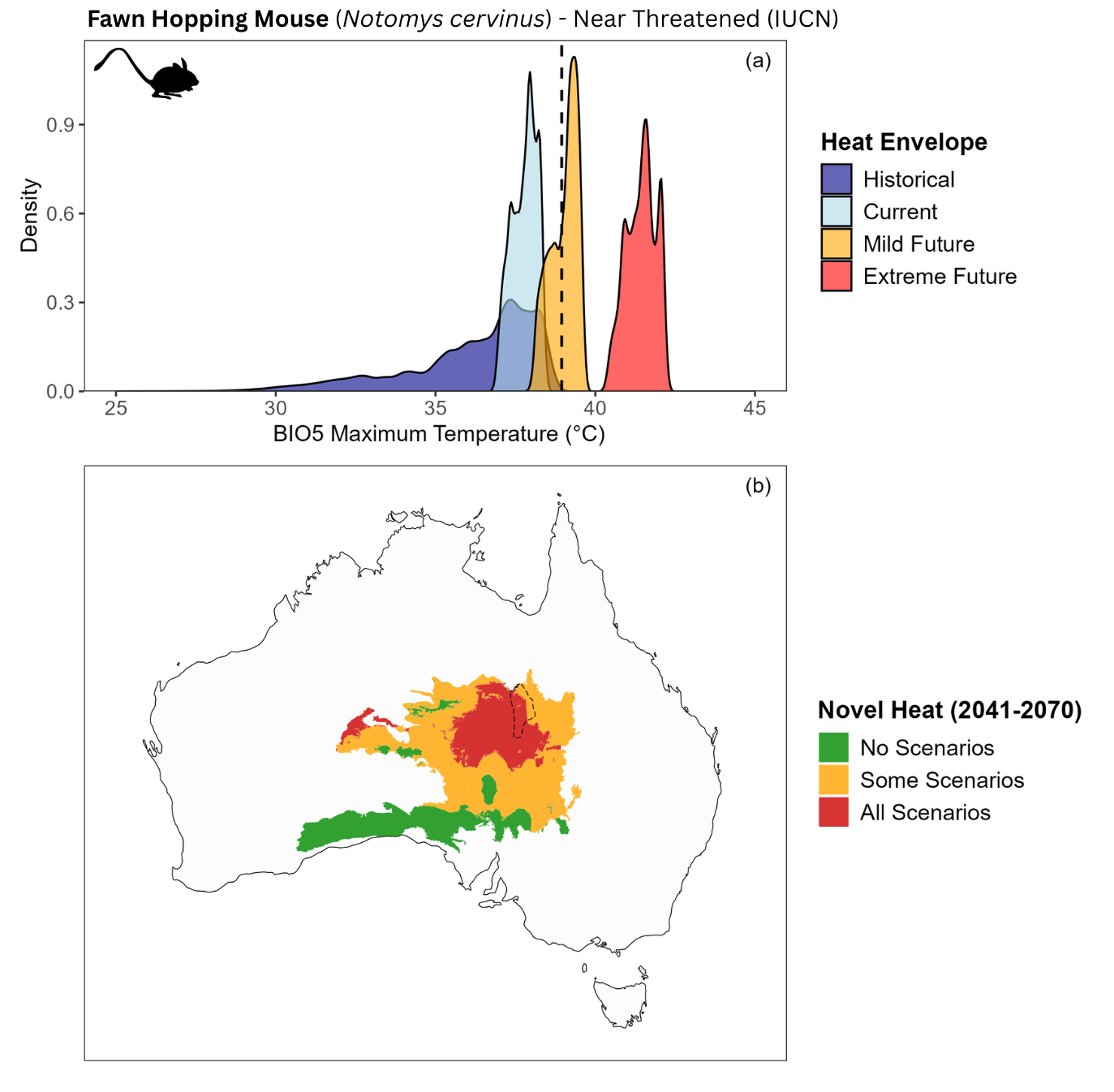
Dotted line indicates current range.*


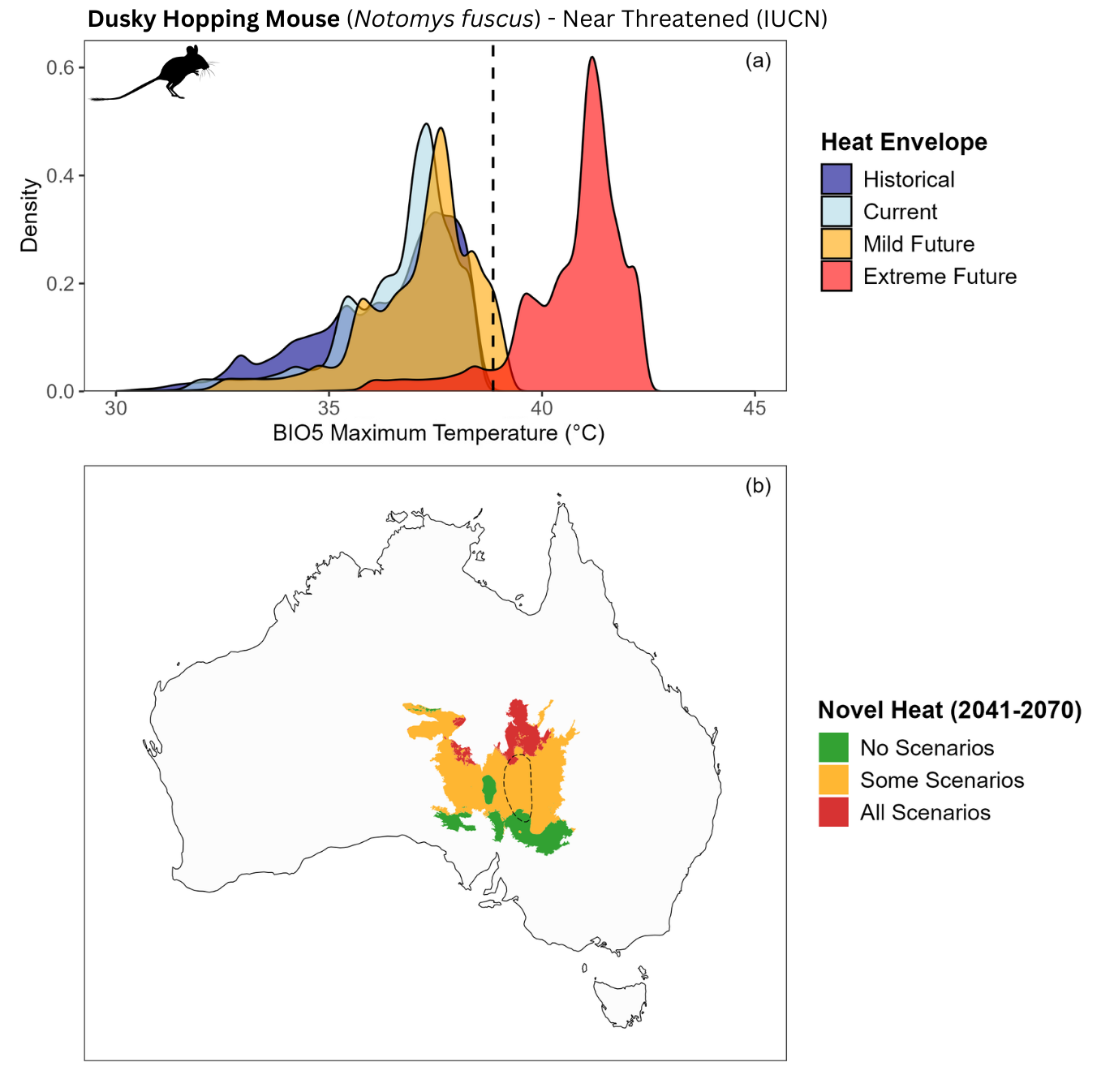
*Dotted line indicates current range.*


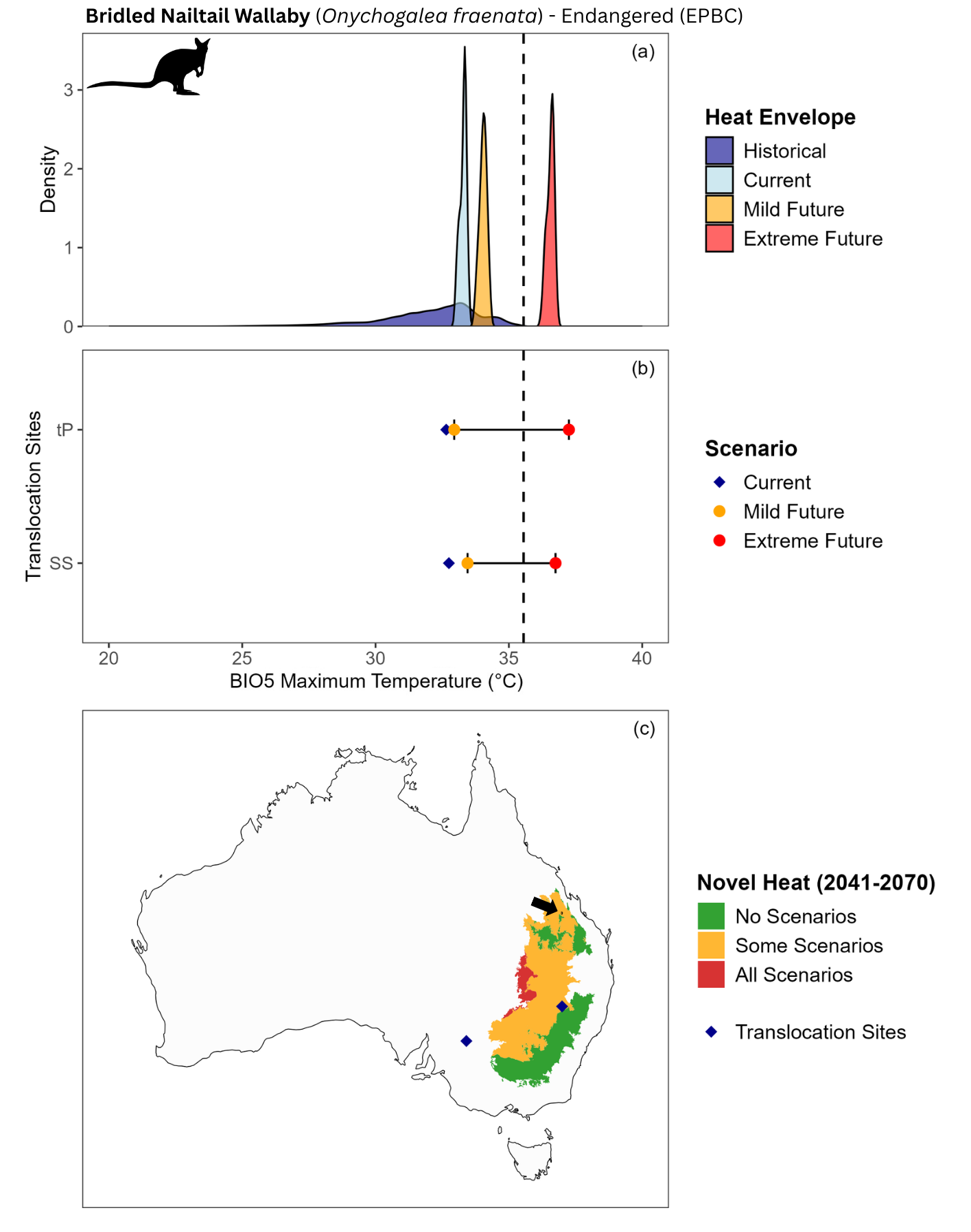

Black arrow indicates current range.

**Translocation sites:** tP = The Pilliga and SS = Scotia Sanctuary.


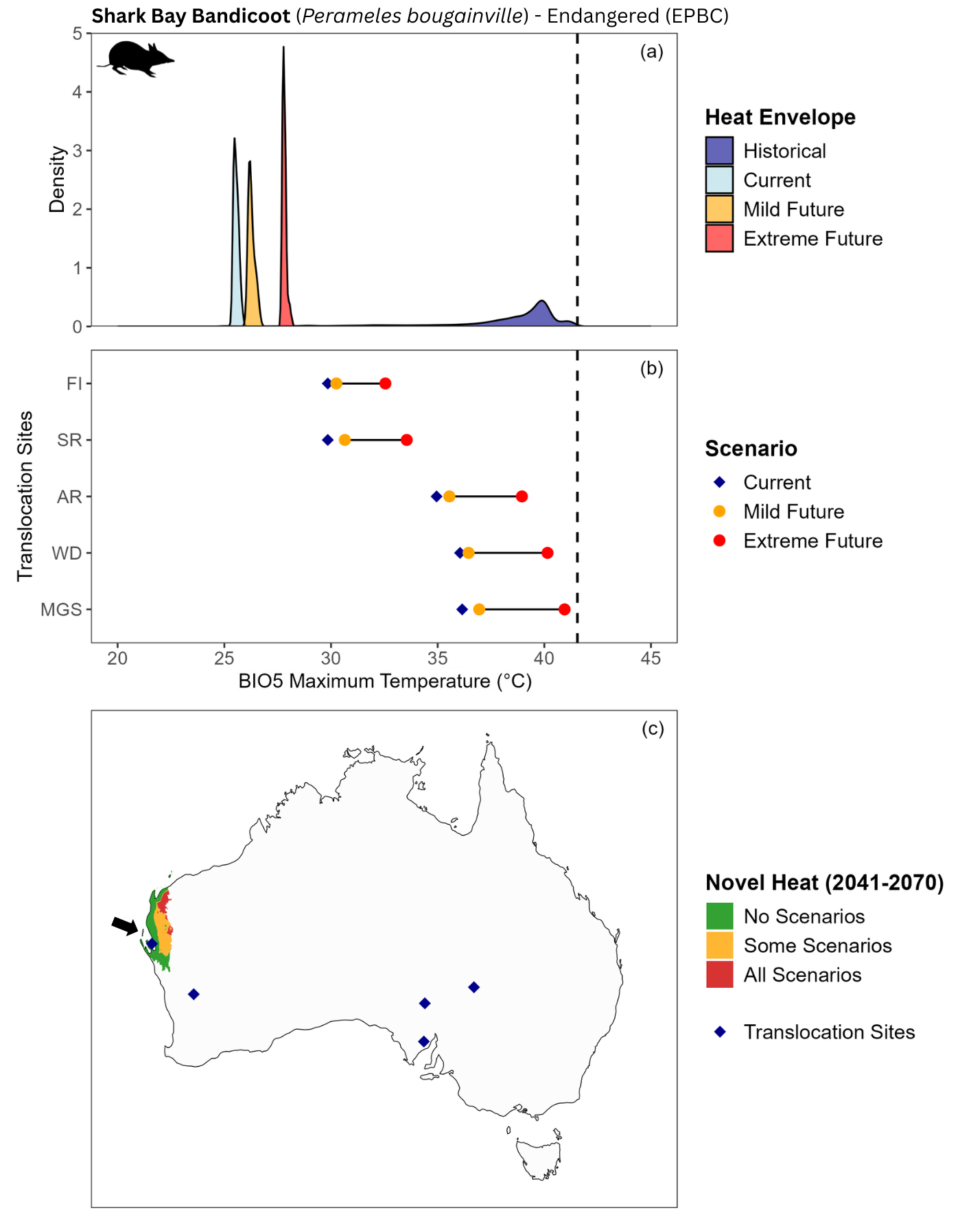

*Black arrow indicates current range.*

**Translocation sites:** FI = Faure Island, SR = Secret Rocks, AR = Arid Recovery, WD = Wild Deserts, and MGS = Mt Gibson Sanctuary.


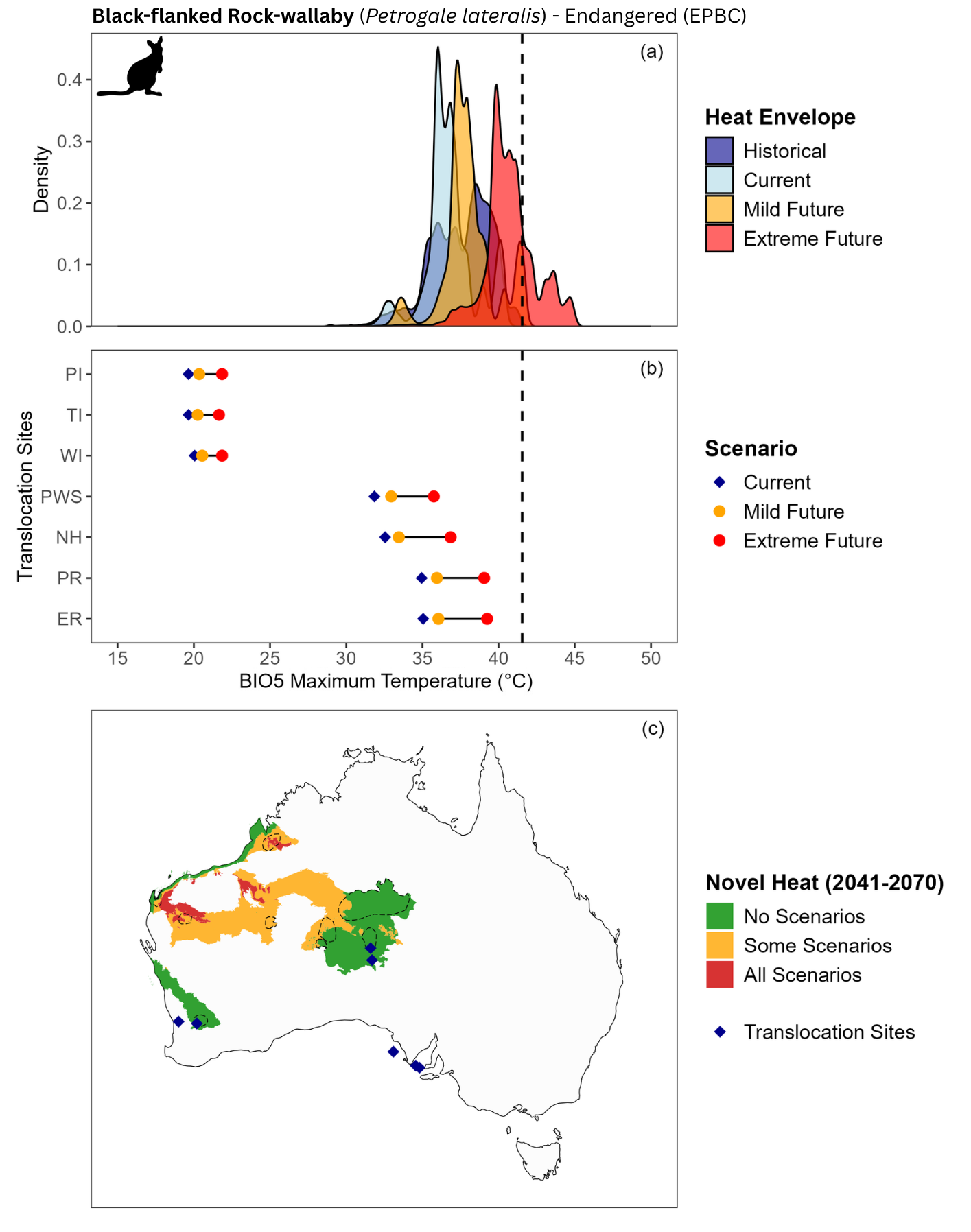

*Dotted line indicates current range*.

**Translocation sites:** PI = Pearson Island, TI = Thistle Island, WI = Wedge Island, PWS = Paruna Wildlife Sanctuary, NH = Nangeen Hill Nature Refuge, PR = Pintji Reserve, and ER = Everard Ranges.


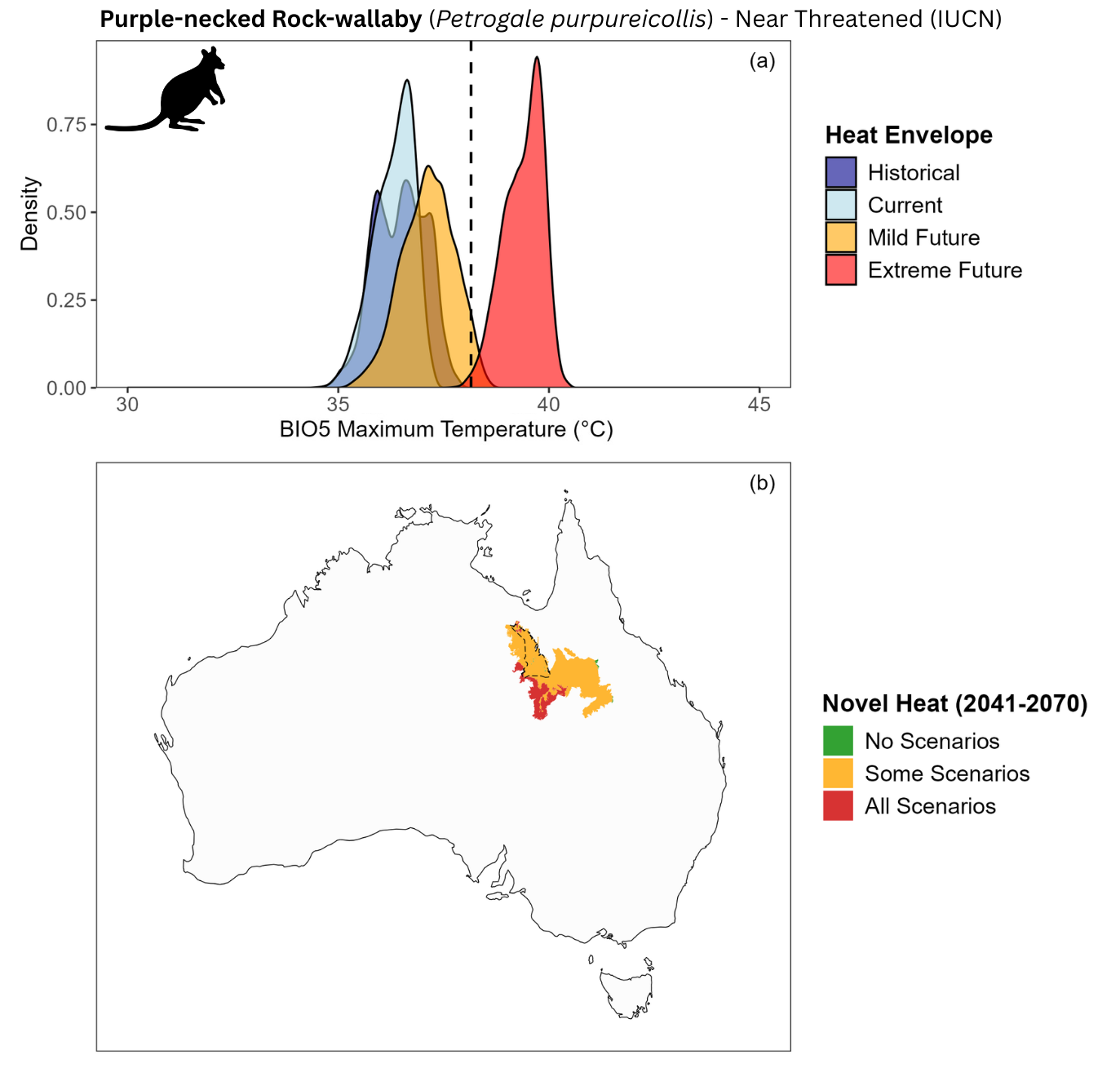
*Dotted line indicates current range.*


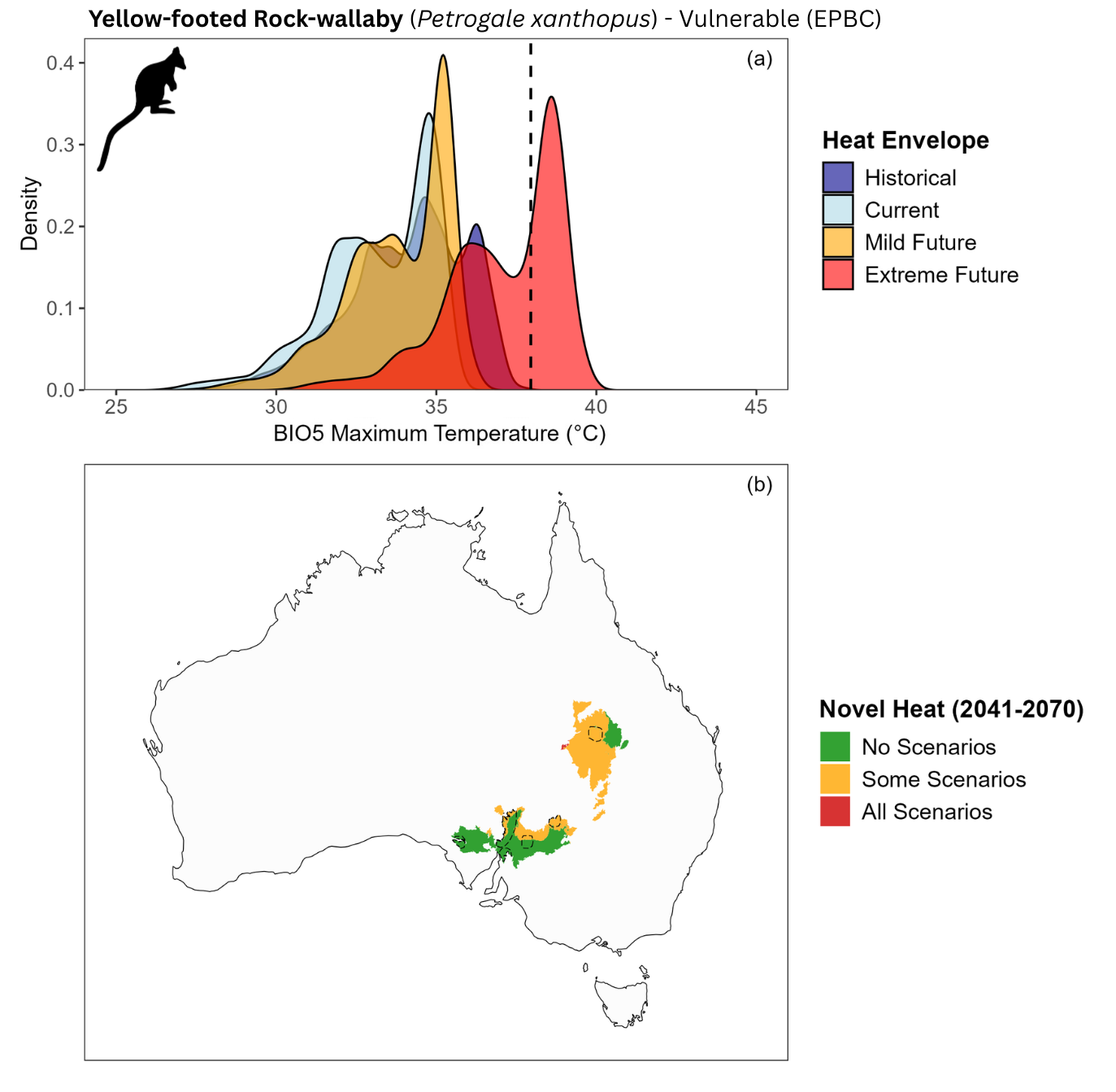

*Dotted line indicates current range.*


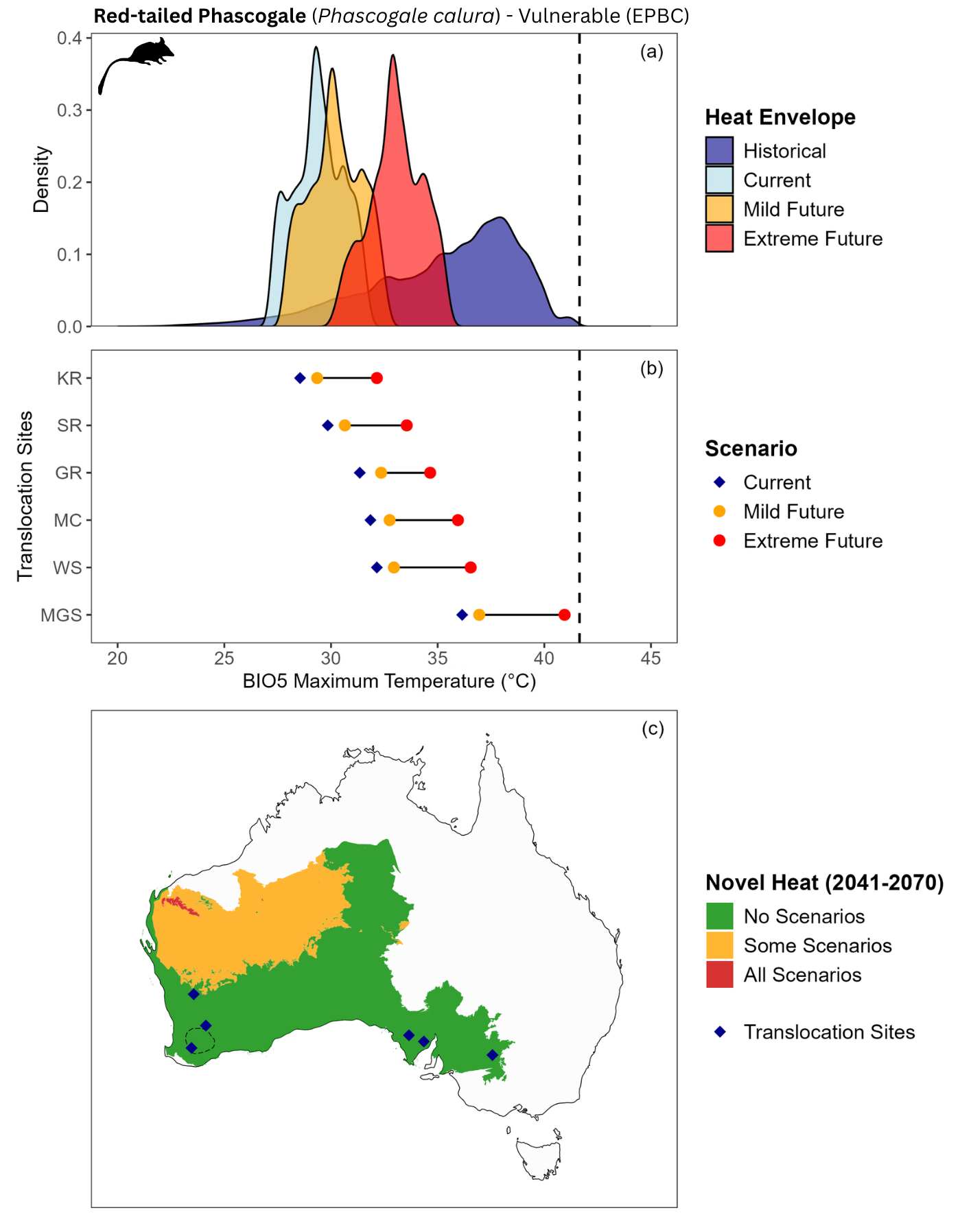

*Dotted line indicates current range.*

**Translocation sites:** KR = Kojonup Reserve, SR = Secret Rocks, GR = Gawler Ranges National Park, MCS = Mallee Cliffs Sanctuary, WS = Wadderin Sanctuary, and MGS = Mt Gibson Sanctuary.


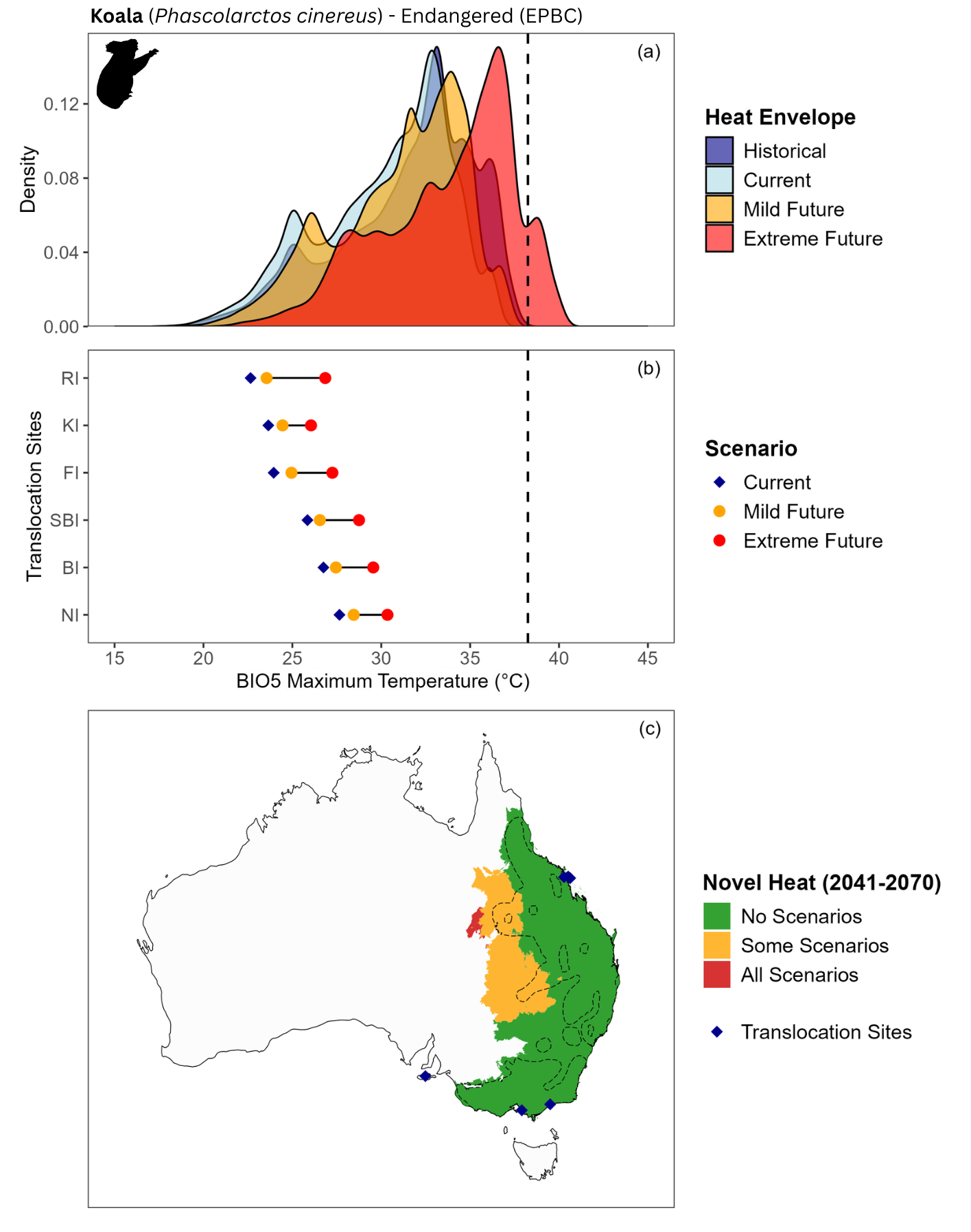

*Dotted line indicates current range. Introduced populations in mainland South Australia are excluded.*

**Translocation sites:** RI = Raymond Island, KI = Kangaroo Island, FI = French Island, SBI = Saint Bees Island, BI = Brampton Island, and NI = Newry Island.


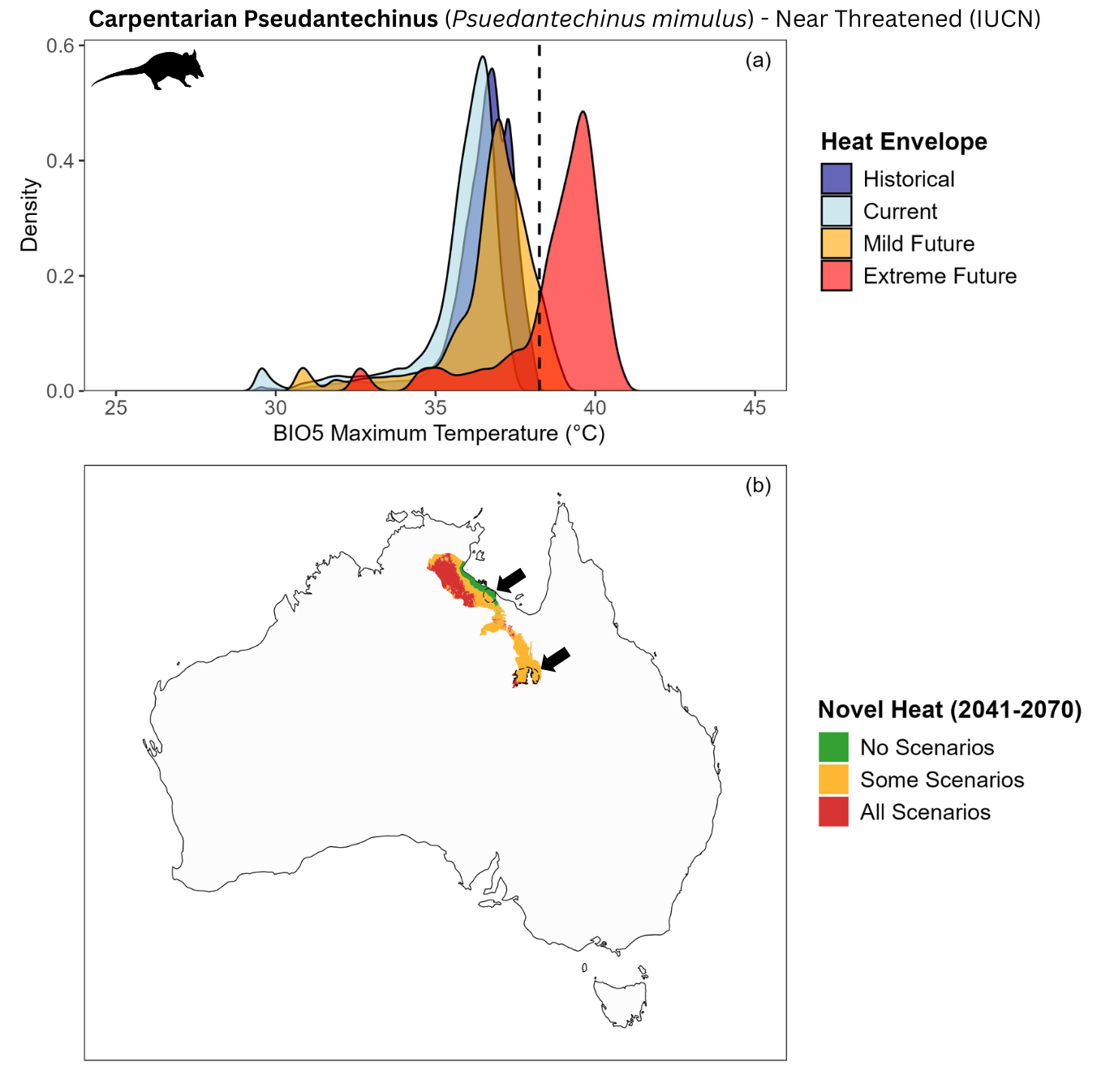

*Black arrows indicate current range.*


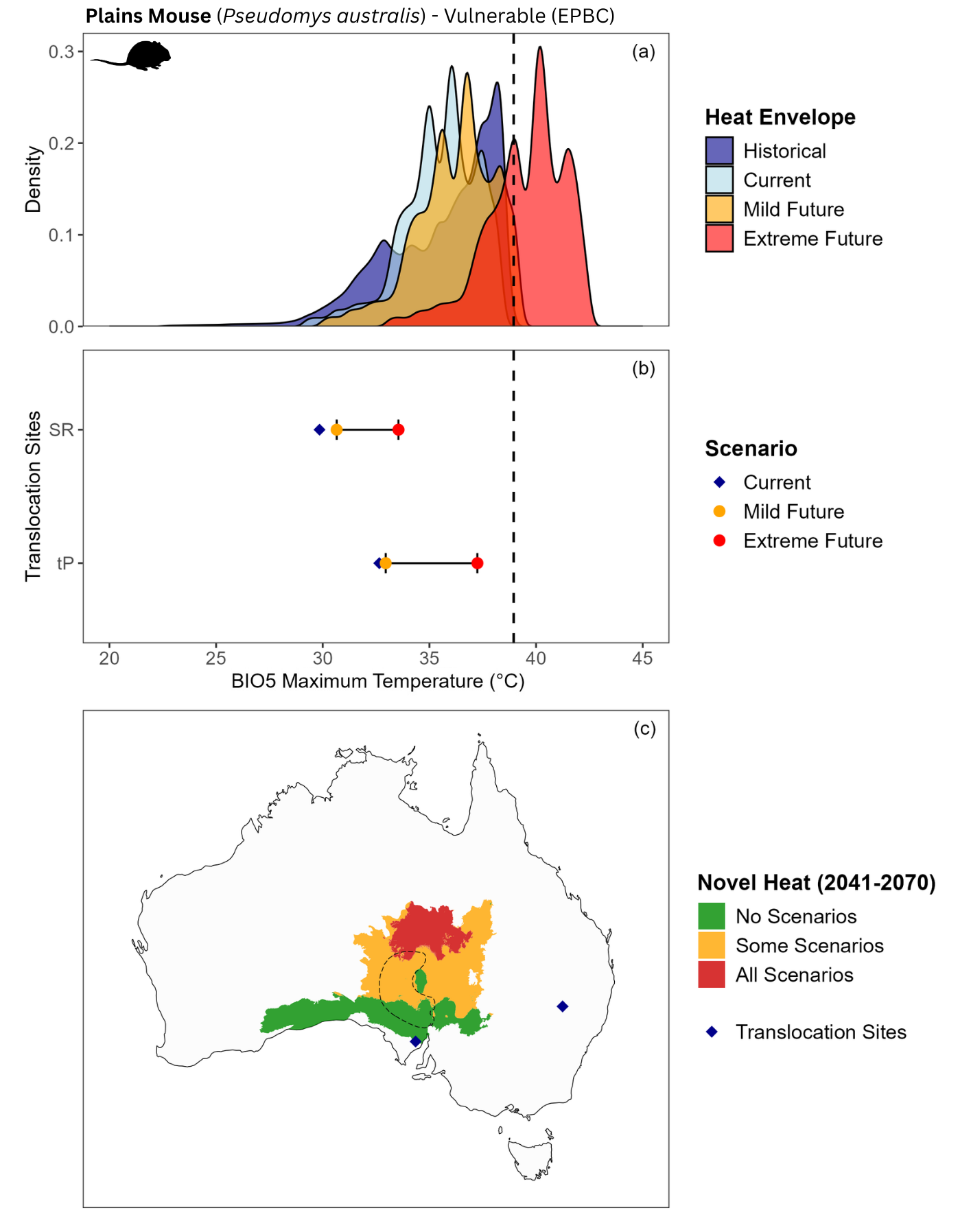

*Dotted line indicates current range.*

**Translocation sites:** SR = Secret Rocks and tP = The Pilliga.


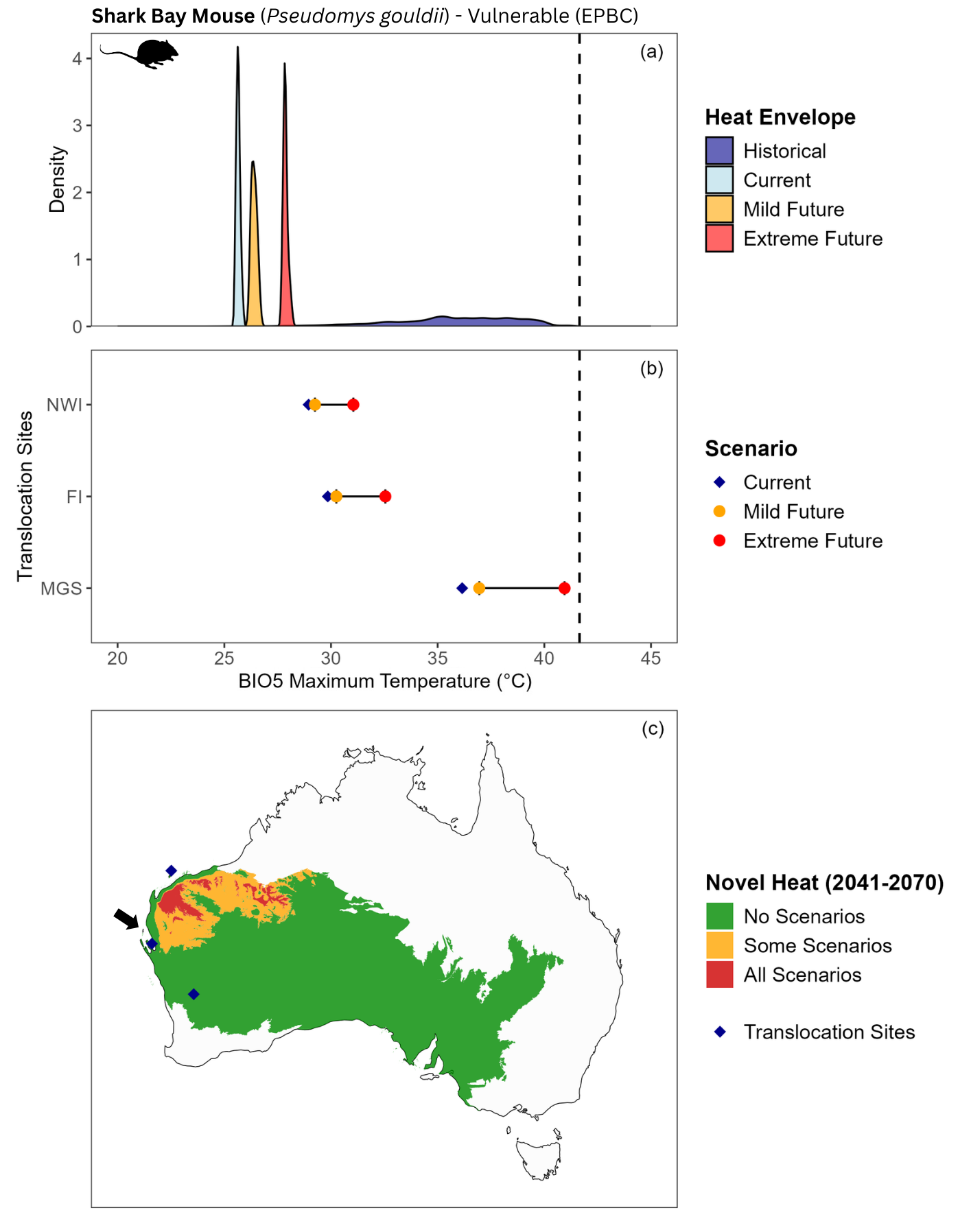

*Black arrow indicates current range.*

**Translocation sites:** NWI = Northwest Island, FI = Faure Island, and MGS = Mt Gibson Sanctuary.


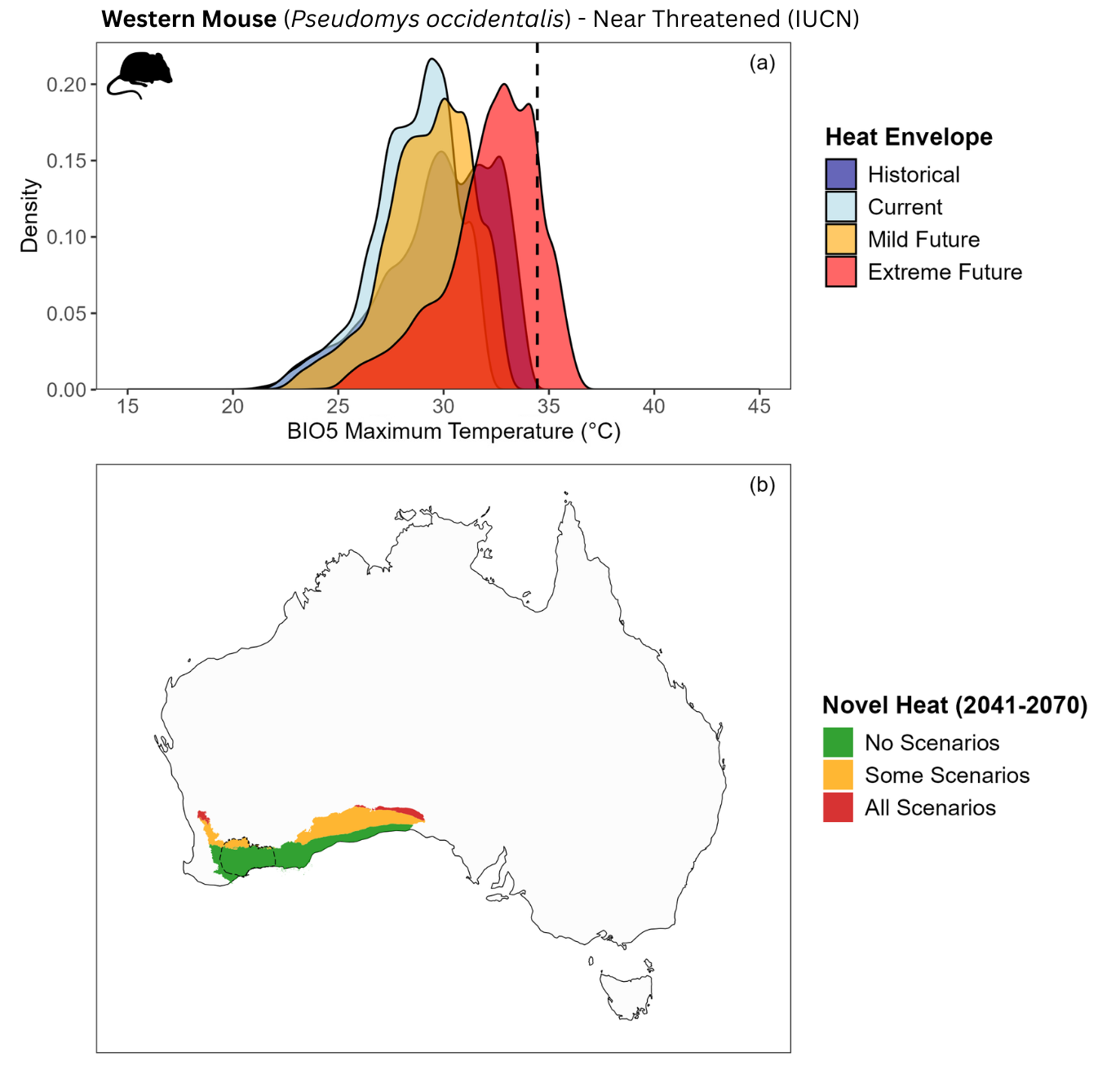

*Dotted line indicates current range.*


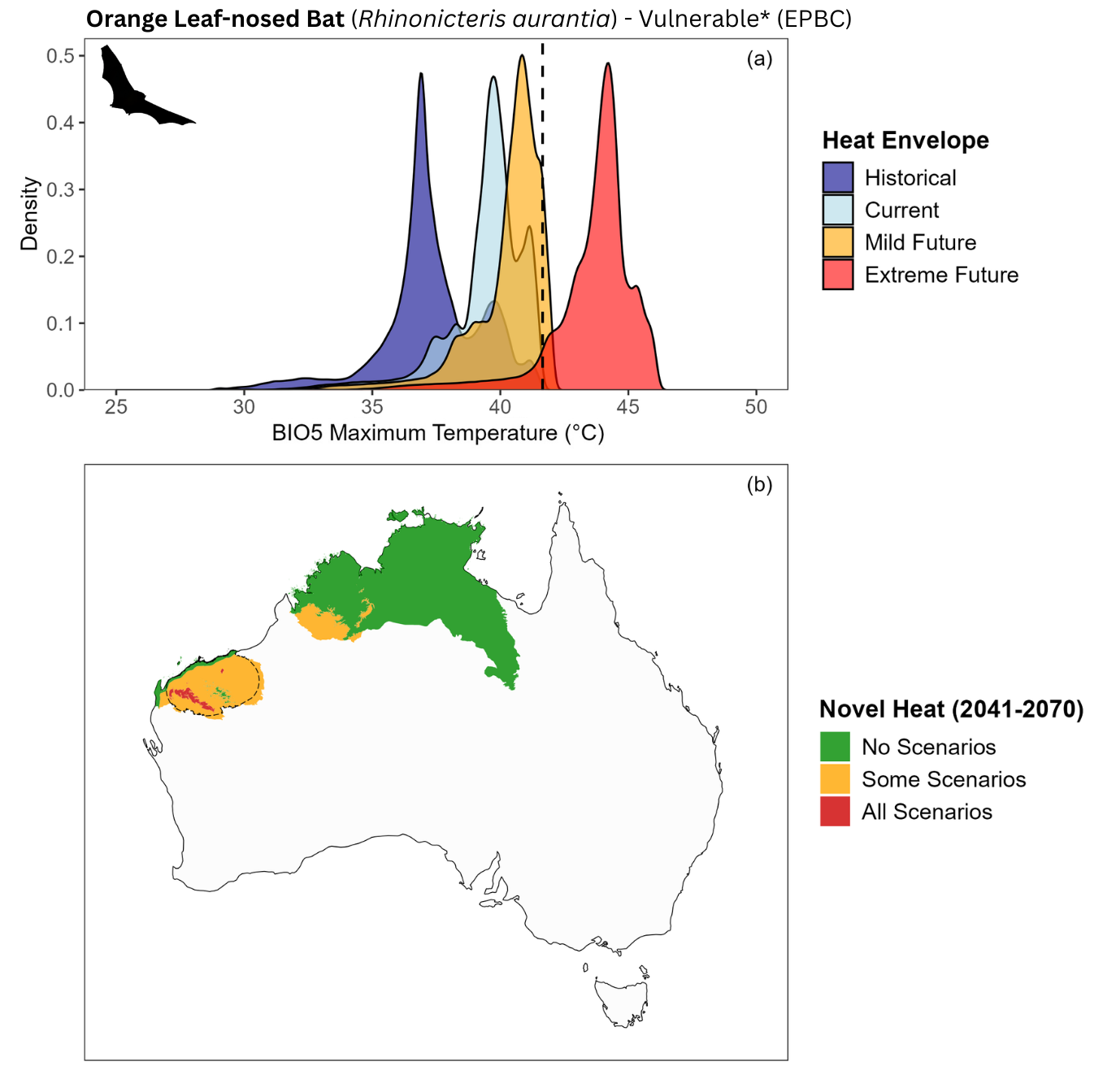

*Dotted line indicates current range of the Vulnerable Pilbara population. For this species, current envelope shows just the range of the Pilbara population while historic range describes the entire range of the species. The thermal maximum is determined from the entire range of the species.*


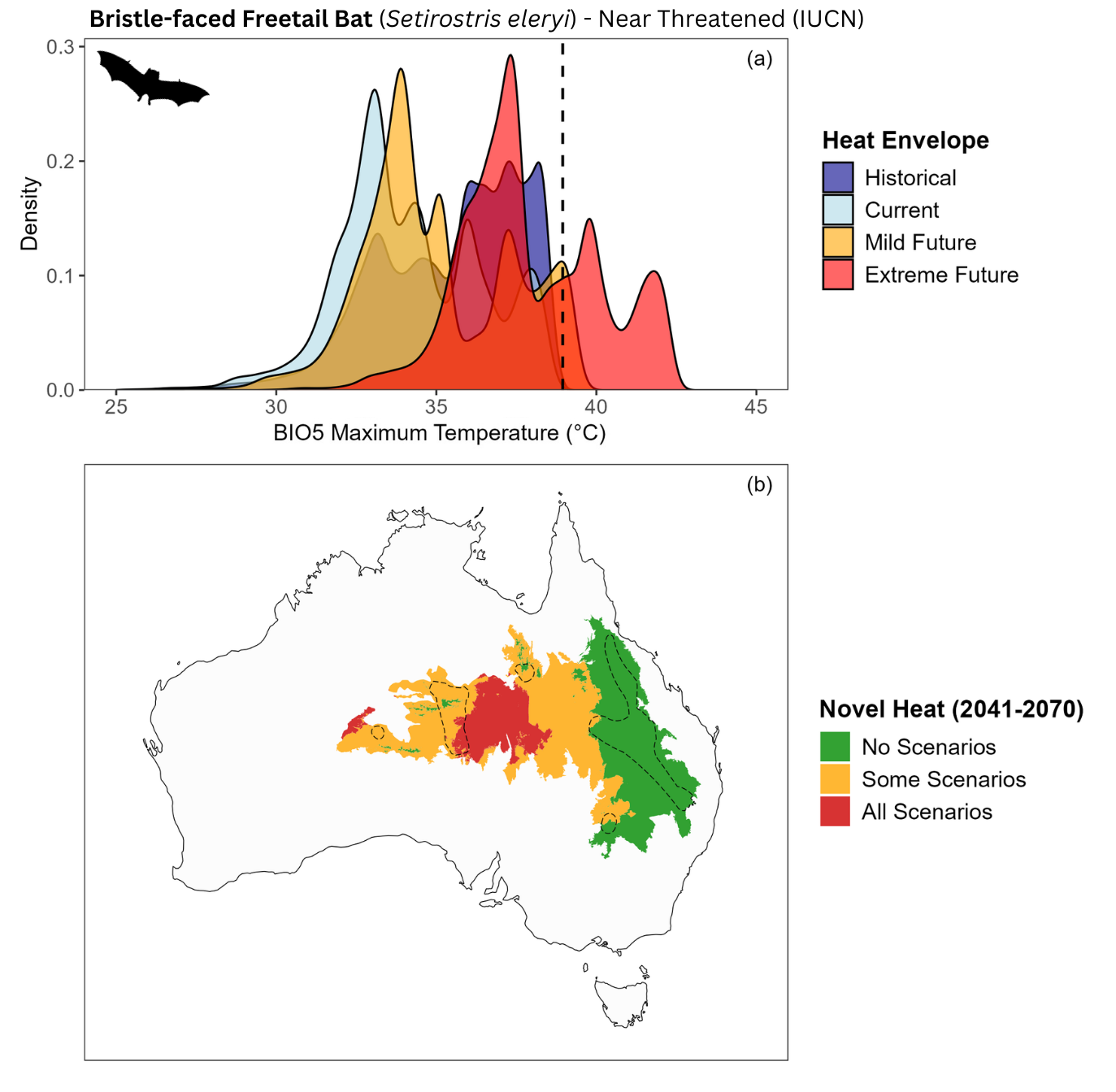

*Dotted line indicates current range.*


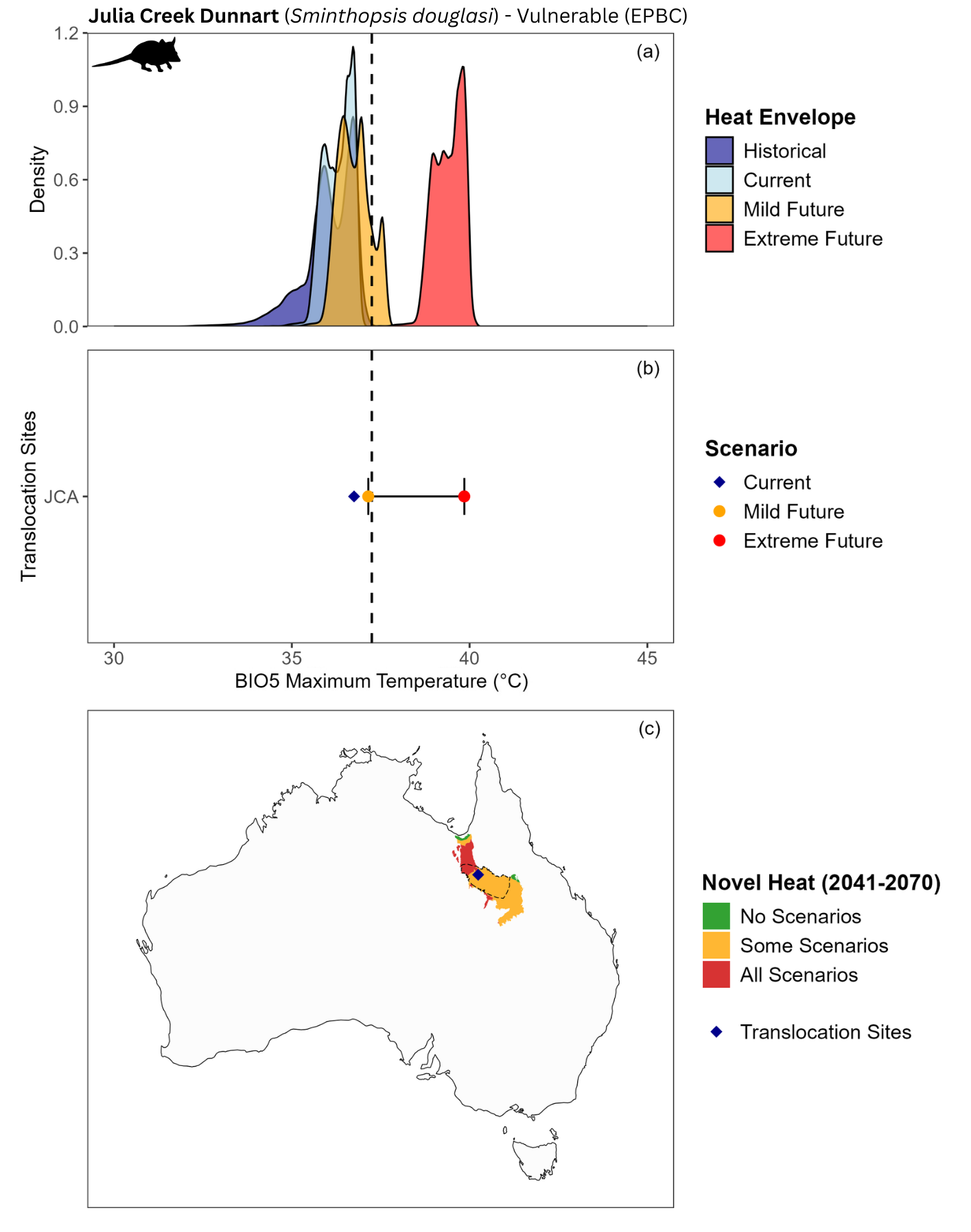

*Dotted line indicates current range.*

**Translocation sites:** JCA = Julia Creek Airport.


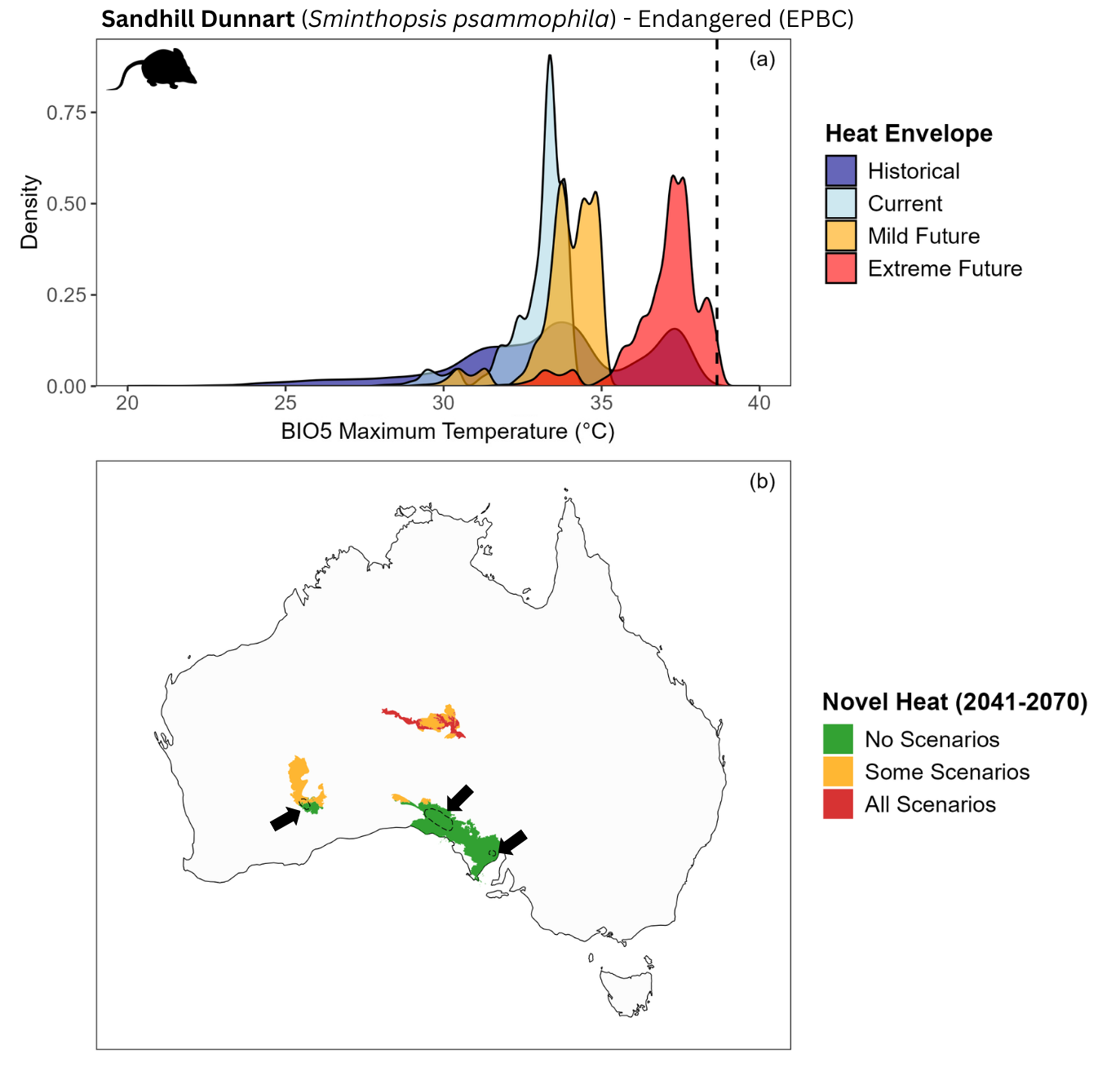

*Black arrows indicate current range.*


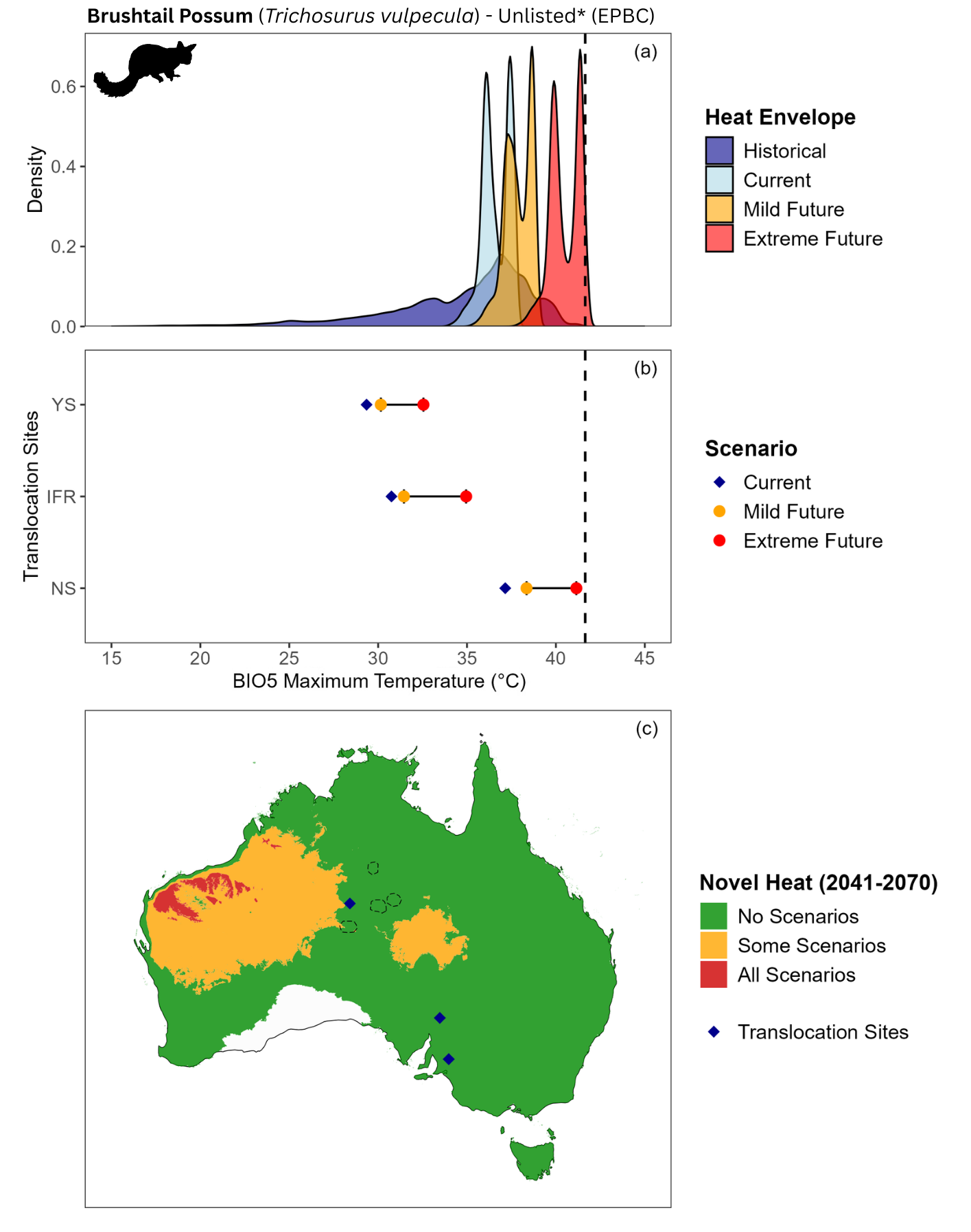

*Dotted line indicates current range of threatened population. Current envelope describes just the range of the central Australian populations while historic range describes the entire range of the species. The thermal maximum is derived from the entire historic range of the species.*

**Translocation sites:** YS = Yookamurra Sanctuary, IFR = Ikara-Flinders Ranges National Park, and NS = Newhaven Sanctuary.


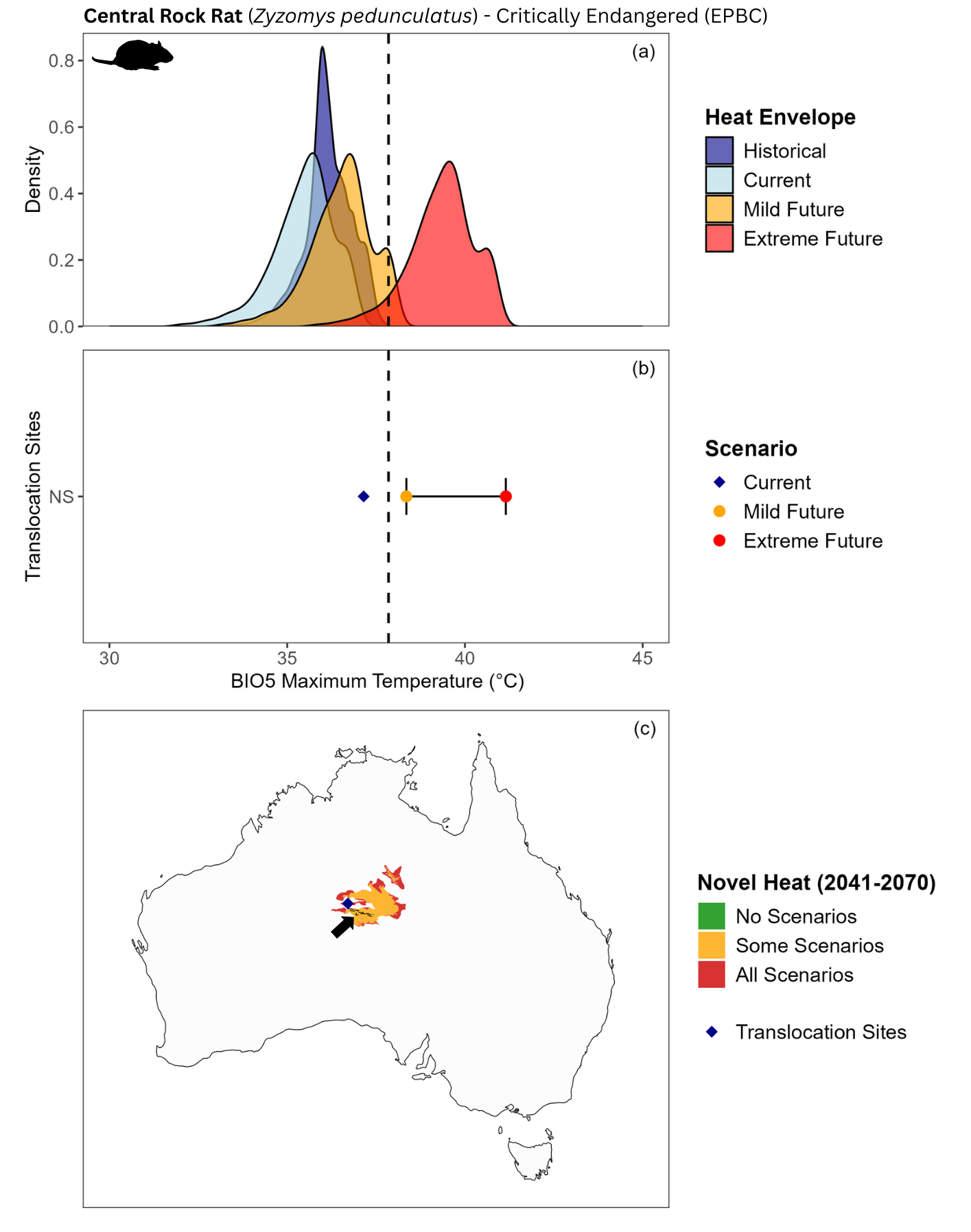

*Black arrow indicates current range.*

**Translocation sites:** NS = Newhaven Sanctuary.
