# Supplementary material for: Estimating the Impacts of Future Extreme Heat on Dryland Threatened Mammals: An Australian Case Study
Source: Glob Chang Biol. 2026 Apr 20;32(4):e70872. doi: 10.1111/gcb.70872 (PMC13094399; doi:10.1111/gcb.70872)

**Appendix S6:** Statistical associations between heat risk category and species-level traits. Chi-squared tests (χ²) were performed for taxonomic order and IUCN status. Taxa: Rodentia (Ro), Dasyuromorphia (Da), Diprontodontia (Di), Peramelemorphia (Pe), Chiroptera (Ch). IUCN: Least Concern (LC), Near Threatened (NT), Vulnerable (VUL), Endangered (END), Critically Endangered (CE). Kruskal-Wallis tests (*H*), with post-hoc Dunn’s tests (*z-*statistic), were performed for numeric predictors.

| **Trait Predictor** | **Chi-Squared Tests** | | |
| --- | --- | --- | --- |
|  | χ² | *df* | *p-value* |
| *Taxonomic Order* | 5.941 | 8 | 0.654 |
| *IUCN Status* | 5.607 | 8 | 0.691 |


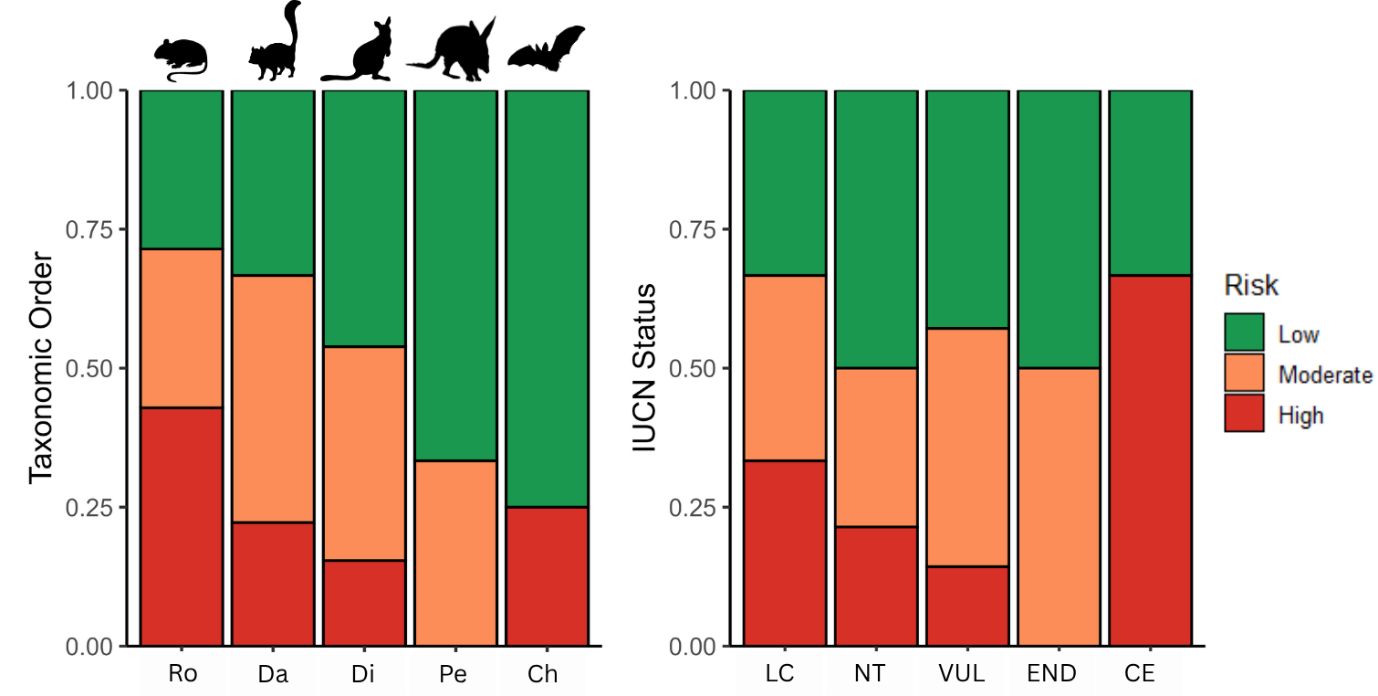


| **Trait Predictor** | **Kruskall-Wallis Test & post-hoc Dunn’s test** | | | |
| --- | --- | --- | --- | --- |
|  | *H* / *z*-values | *df* | *p-value* | *Significance* |
| *Body Size (log-grams)* | 0.848 | 2 | 0.654 | ns |
| High-Low | -0.911 |  | 1.000 | ns |
| High-Moderate | -0.680 |  | 1.000 | ns |
| Low-Moderate | 0.220 |  | 1.000 | ns |
| *Current Extant (log-km^2^)* | 18.082 | 2 | < .001 | *** |
| High-Low | -1.987 |  | 0.141 | ns |
| High-Moderate | 1.655 |  | 0.294 | ns |
| Low-Moderate | 4.232 |  | < .001 | *** |
| *Proportion in Arid* | 3.962 | 2 | 0.138 | ns |
| High-Low | 1.143 |  | 0.759 | ns |
| High-Moderate | -0.539 |  | 1.000 | ns |
| Low-Moderate | -1.941 |  | 0.157 | ns |
| *Historical Proportion in Arid* | 0.751 | 2 | 0.687 | ns |
| High-Low | 0.274 |  | 1.000 | ns |
| High-Moderate | -0.464 |  | 1.000 | ns |
| Low-Moderate | -0.865 |  | 1.000 | ns |
| *Total Decline (%)* | 15.271 | 2 | < .001 | *** |
| High-Low | 1.295 |  | 0.585 | ns |
| High-Moderate | -1.951 |  | 0.153 | ns |
| Low-Moderate | -3.907 |  | < .001 | *** |
| *TMax_Hist_ – Tmax_Current_* | 14.578 | 2 | < .001 | *** |
| High-Low | -1.238 |  | 0.647 | ns |
| High-Moderate | -3.546 |  | < 0.001 | *** |
| Low-Moderate | -2.945 |  | 0.010 | * |
| *Historical Envelope Breadth* | 13.350 | 2 | 0.001 | *** |
| High-Low | -3.319 |  | 0.003 | ** |
| High-Moderate | -3.323 |  | 0.003 | ** |
| Low-Moderate | -0.262 |  | 1.000 | ns |
| *Current Envelope Breadth* | 20.916 | 2 | < .001 | *** |
| High-Low | -3.070 |  | 0.006 | ** |
| High-Moderate | 0.685 |  | 1.00 | ns |
| Low-Moderate | 4.299 |  | < .001 | *** |

Significance indicators: ns p > 0.05, * p ≤ 0.05, ** p ≤ .01, *** p ≤ .001.


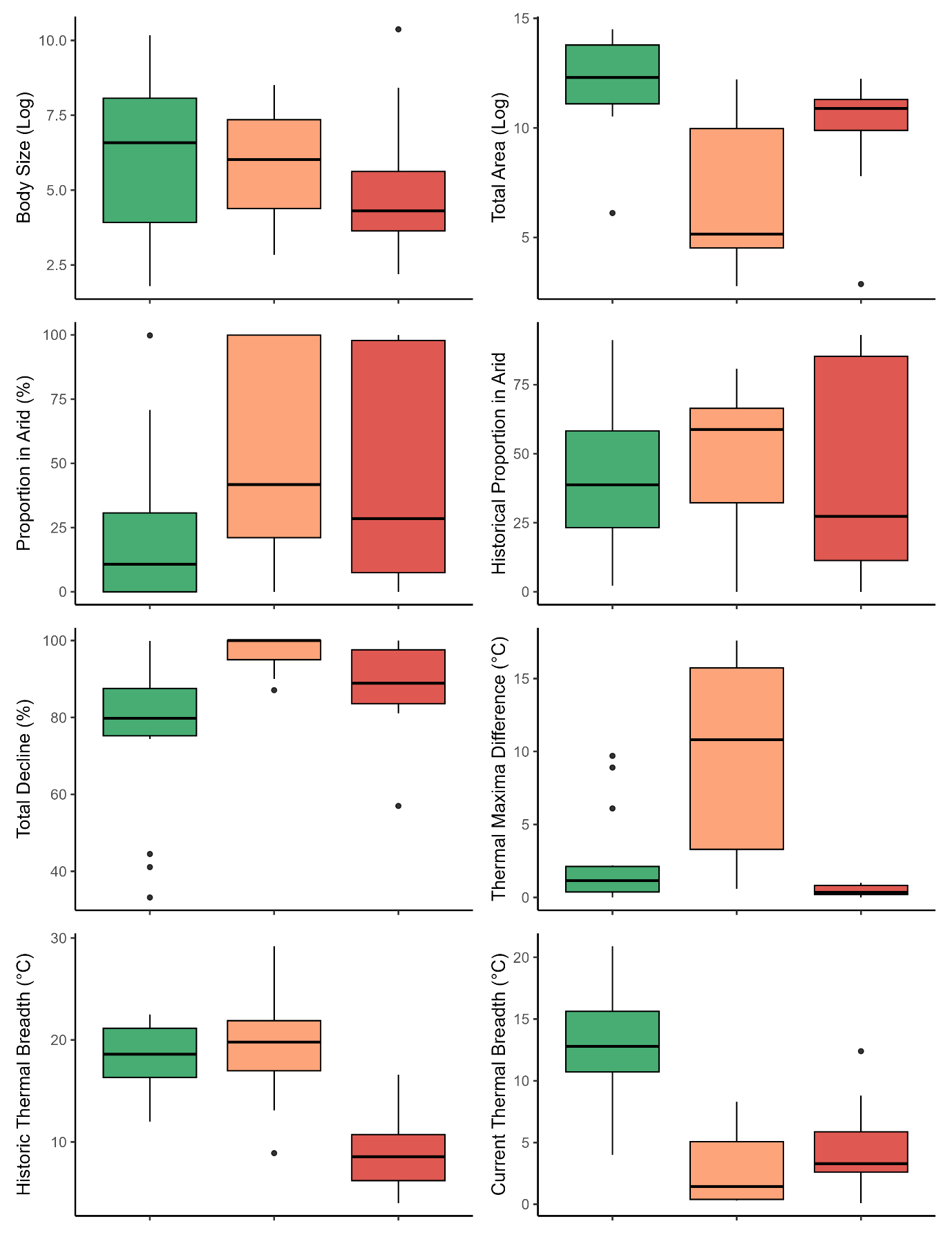

Supplement: Supplementary file 6 — Appendix S6: Statistical associations between heat risk category and species‐level traits. Chi‐squared tests (χ 2) were performed for taxonomic order and IUCN status. Taxa: Ch, Chiroptera; Da, Dasyuromorphia; Di, Diprotodontia; Pe, Peramelemorphia; Ro, Rodentia. IUCN: CE, Critically Endangered; END, Endangered; LC, Least Concern; NT, Near Threatened; VUL, Vulnerable. Kruskal‐Wallis tests (H), with post hoc Dunn's tests (z‐statistic), were performed for numeric predictors. [file GCB-32-e70872-s007.docx]
